# Prevalence of Naturally-Occurring NS5A and NS5B Resistance-Associated Substitutions in Iranian Patients With Chronic Hepatitis C Infection

**DOI:** 10.3389/fmicb.2020.617375

**Published:** 2021-01-28

**Authors:** Pooneh Rahimi, Heidar Sharafi, Golnaz Bahramali, FaridehSadat SajadianFard, Nafiseh Sadat Asadi, Seyed Moayed Alavian, Vahid Iranpur Mobarakeh, Seyedeh Zahra Moravej

**Affiliations:** ^1^Department of Hepatitis and AIDS, Pasteur Institute of Iran, Tehran, Iran; ^2^Middle East Liver Diseases Center, Tehran, Iran

**Keywords:** HCV, Direct-acting antiviral agents, NS5A, NS5B, Resistance-associated substitution

## Abstract

**Background:**

Hepatitis C virus (HCV), non-structural 5A (NS5A), and non-structural 5B (NS5B) resistance-associated substitutions (RASs) are the main causes of failure to direct-acting antiviral agents (DAAs). NS5A and NS5B RASs can occur in patients with HCV infection naturally and before exposure to DAAs.

**Objectives:**

This study aimed to evaluate naturally-occurring NS5A and NS5B RASs in Iranian patients with HCV genotype 1a (HCV-1a) and -3a infections.

**Methods:**

In this cross-sectional study, viral RNA was extracted from serum specimens. NS5A and NS5B regions were amplified using RT-PCR followed by DNA sequencing. The results of nucleotide sequences were aligned against reference sequences of HCV-1a and -3a and the amino acid substitutions were analyzed using geno2pheno [hcv] web application.

**Results:**

Among 135 patients with hepatitis C, NS5A amino acid substitutions/RASs were identified in 26.4% and 15.9% of patients with HCV-1a and -3a infections, respectively. The identified amino acid substitutions/RASs in the NS5A region of patients with HCV-1a infection were M28T/V/I 11.1%, Q30R/H 4.2%, L31M 1.4%, and H58Y/P/C/D/Q/S/T 16.7%. Y93H substitution was not found in HCV-1a sequences. In patients with HCV-3a infection, NS5A amino acid substitutions/RASs were A30T/K 9.5%, L31F 1.6%, P58S/T/C 3.2%, Y93H 3.2%, and Y93N 3.2%. No resistance substitutions were identified in NS5B sequences from patients with HCV-1a and -3a infections.

**Conclusion:**

In this study, baseline amino acid substitutions/RASs were only identified in the NS5A region in Iranian patients with HCV-1a and -3a infections, and the prevalence of these amino acid substitutions/RASs were in accordance with similar studies. There were no RASs in the HCV-1a and -3a NS5B region.

## Introduction

It has been estimated that approximately 71 million people worldwide are chronically infected with the hepatitis C virus (HCV). This viral infection is the leading cause of acute and chronic hepatitis with a spectrum of mild illness to end-stage liver disease such as hepatocellular carcinoma (HCC) with about 399,000 deaths each year (Bittar et al., 2010; Paolucci et al., 2013; Blach et al., 2017). The virus belongs to the Hepacivirus genus in the Flaviviridae family with a genome of positive-sense ssRNA with approximately 9.6 Kb encoding for a polyprotein precursor which is cleaved by the viral and host proteinases into the structural (core, E1, E2, and P7) and the non-structural proteins (NS2, NS3, NS4A, NS4B, NS5A, and NS5B) (Bittar et al., 2010; Paolucci et al., 2013; Blach et al., 2017). HCV is one of the blood-borne viruses; so, the most common modes of transmission are through exposure to small quantities of blood or its derivatives that make this infection one the worldwide public health problems (Paolucci et al., 2013; Khodabandehloo and Roshani, 2014; Taherkhani and Farshadpour, 2015; Blach et al., 2017; Mahmud et al., 2018). The prevalence of HCV infection in Iran is estimated between 0.3 and 0.5% among the general population to 32.1% among high-risk populations (Khodabandehloo and Roshani, 2014; Taherkhani and Farshadpour, 2015; Mahmud et al., 2018).

Up to now, seven genotypes with many subtypes have been identified for HCV and the distribution of these genotypes is varying between different populations (Murphy et al., 2007, 2015; Kau et al., 2008; Sarrazin and Zeuzem, 2010; Nakano et al., 2012; Smith et al., 2014). According to the molecular epidemiology studies, HCV genotype 1a (HCV-1a), HCV-3a, and with the lower frequency HCV-1b are the most prevalent HCV genotypes in Iran (Khodabandehloo and Roshani, 2014; Taherkhani and Farshadpour, 2015; Mahmud et al., 2018). Viral RNA-dependent RNA polymerase of HCV which lacks 3′ to 5′ exonuclease proofreading activity leads to a high genome replication rate with no fidelity, and enables HCV to escape from selective pressures of the immune response and antiviral therapies (Kau et al., 2008; Paolucci et al., 2013; Murphy et al., 2015; de Rueda et al., 2017). All these characteristics make HCV a challenging target for designing effective vaccine and antiviral drugs (Murphy et al., 2007; Gottwein et al., 2009; Nakano et al., 2012; Smith et al., 2014; Walker et al., 2015; Alavian and Sharafi, 2017; de Rueda et al., 2017; Mahmud et al., 2018). For many years, pegylated interferon (PegIFN) plus ribavirin (RBV) has been used for the treatment of chronic HCV infection. However, treatment with PegIFN and RBV had undesirable side-effects and suboptimal responses or even failure to treatment especially in patients with HCV-1 and -4 and those with cirrhosis (Kau et al., 2008; Sarrazin and Zeuzem, 2010). Recently, direct-acting antiviral agents (DAAs) have been developed against different functional proteins of HCV, which seem to be promising. However, the efficiency of these drugs could be impacted negatively by the existence of resistance-associated substitutions (RASs) both at the baseline before initiation of treatment or re-treatment following the previous failure to DAA-based regimens, infection with HCV genotypes that used to be known as hard-to-treat genotypes and cirrhosis (Mahmud et al., 2018; Ghany et al., 2019). Although using a combination of DAAs results in inhibition of HCV replication efficiently, the mutable nature of the HCV genome makes it necessary to put the occurrence of emerging substitutions associated with DAAs resistance in patients with HCV infection under precise surveillance. Herein, we report the naturally-occurring NS5A and NS5B RASs causing resistance to DAAs in a cohort of DAA-naïve patients with chronic HCV-1a and -3a infections.

## Materials and Methods

### Study Population and Sample Collection

This cross-sectional study recruited patients managed at the hepatitis clinic of Digestive Disease Research Institute (DDRI), Tehran, Iran from 2015 to 2017. In this study, adults (>18 years old) with chronic HCV-1a and -3a infections were included. Hepatitis C chronicity was defined as being positive for HCVAb and HCV RNA for more than 6 months. Co-infection with HIV and/or HBV or any condition that leads to being immunocompromised, being diagnosed with HCC, and having a previous history of treatment with DAAs were considered as exclusion criteria. Cirrhosis was diagnosed based on clinical or histological measurements or with non-invasive assessment by transient elastography. The baseline HCV RNA level and HCV genotype were assessed before the initiation of HCV antiviral therapy and the results were available as the routine workup for diagnosis and management of HCV infection in patients’ records. These tests were carried out in the diagnostic laboratories as commercial accredited services. The blood sampling procedures were explained to the patients clearly and the consent form for using their blood samples for further analysis was signed by each volunteer or their official custodian. This study was approved by the Ethics Committee of the Pasteur Institute of Iran (no: IR.PII.REC.1395.81) according to the standard biosecurity and institutional safety procedures. The current study was conducted according to the Helsinki Declaration of 1975, as revised in 2008.

### HCV Genome Extraction and Amplification

Viral RNA was extracted from 140 μL of baseline serum samples using a commercially available kit (HighPure Viral Nucleic Acid kit, Roche, Germany). cDNA synthesis and first-round PCR amplifications were done in a 25 μL reaction mixture using PrimeScript^TM^ One-Step RT-PCR Kit (TaKaRa, Clontech, Japan). The following primers were used for the amplification of the HCV-1a NS5A region: FO1ans5a and RO1ans5a (Supplementary Table 1). NS5B region is a long region with approximately 1776 nucleotides. To study the most possible complete sequences of this region, we divided it into two sub-regions as NS5B1 and NS5B2 by designing specific primers for each region: FO1ans5b1 and RO1ans5b1 (NS5B1), and FO1ans5b2 and RO1ans5b2 (NS5B2; Supplementary Table 1). The amplification reaction was performed according to the manufacturer’s instruction following the program: incubation at 50°C for 30 min and initial denaturation at 94°C for 2 min, then 35 cycles of denaturation at 94°C for 30 s, annealing at 59°C (for NS5A outer primers), 61°C (for NS5B1 outer primers), and 59°C (for NS5B2 outer primers) for 30 s, extension at 72°C for 1 min and a final extension at 72°C for 10 min. Then, the second round of PCR was performed using TaKaRa Ex Taq^TM^ kit (TaKaRa, Clontech, Japan) and specific inner primers for each region resulted in specific PCR products as a 510 bp fragment for NS5A, and a 797 bp and a 928 bp PCR product for NS5B1 and NS5B2, respectively, (Supplementary Table 1). This amplification was performed in a 50 μL reaction mixture according to the manufacturer’s instruction and the program was initiated with denaturation at 94°C for 5 min and 35 cycles including denaturation at 94°C for 1 min, annealing at 60°C (for NS5A inner primers), 59°C (for NS5B1 inner primers), and 58°C (for NS5B2 inner primers) for 30 s, extension at 72°C for 7 min and a final extension at 72°C for 10 min.

Similar procedures were executed for the amplification of NS5A and NS5B regions from HCV-3a using specific primers. Briefly, specific outer primers were used for the first round of NS5A and NS5B including FO3ans5a and RO3ans5a (NS5A), FO3ans5b1 and RO3ans5b1 (NS5B1), and FO3ans5b2 and RO3ans5b2 (NS5B2; Supplementary Table 1). The PCR program was performed in the same way according to the manufacturer’s instruction as it was done for HCV-1a except for the annealing temperatures which were 61°C for NS5A, 59°C for NS5B1, and 63°C for NS5B2 amplification. Those first-round PCR products were used as templates for the nested round of PCR by using inner primers including FI3ans5a and RI3ans5a (NS5A), FI3ans5b1 and RI3ans5b1 (NS5B1), and FI3ans5b2 and RI3ans5b2 (NS5B2; Supplementary Table 1). The amplification procedure for the nested round of PCR was the same as the nested round of PCR for the amplification of these regions of HCV-1a. The annealing temperature for NS5A was 60°C and 58°C was used to amplify inner parts of both NS5B1 and NS5B2. The specific bands of HCV-3a NS5A, NS5B1, and NS5B2 were identified with 348, 691, and 768 bp of PCR products, respectively. The PCR products underwent electrophoresis on a 1.5% agarose gel containing safe stain (YTA, Iran) and the specific bands were purified using the Yekta Tajhiz Azma Gel and PCR purification kit (YTA, Iran). Then the purified PCR fragments accompanied with the related specific inner primers for each region were sent to GenFanavaran company to be sequenced (Sanger sequencing) in South Korea in both directions using a BigDye Terminator cycle sequencing kit (Perkin Elmer–Applied Biosystems Inc., CA, United States). The chromatogram was used to assess the nucleotide substitutions in whole sequences, and the nucleotide redundancy was considered when representing ≥15% of the sequence population.

Nucleotide sequences were first analyzed by Bioedit software (v.7.9.5). Then they were aligned against the HCV-1a reference sequences (GenBank accession numbers: EU255982.1, KX621456.1, JX463555.1, KJ747896.1, and HQ891277.1), and the HCV-3a reference sequences (GenBank accession numbers: GQ275355.1, KJ470615.1, HW121730.1, JN689927.1, and KF944665.1) separately using ClustalW integrated into MEGA 6 software. Amino acid positions, RASs, and RASs > 100X were selected using the geno2pheno [HCV] rules (GENAFOR, 2019; Table 1). Geno2pheno [HCV] (publically available at http://hcv.geno2pheno.org) is a basic tool for interpretation and evaluation of viral sequences for susceptibility to anti-HCV DAAs. The same analysis was done to investigate the RASs in the NS5B region for each HCV-1a and -3a, separately (Hernandez et al., 2013; Ghany et al., 2019, 2020). The HCV-1a and -3a sequences obtained by Sanger sequencing in this study have been deposited in the GenBank under accession numbers; HCV-1a NS5A; MT259593-MT259659, HCV-1a NS5B: MT454922-MT455006, HCV-3a NS5A: MT347782-MT347803, and HCV-3a NS5B: MT502204-MT502244, respectively.

**TABLE 1:** The amino acid positions, consensus amino acids, amino acid substitutions, resistance-associated substitutions (RASs), and the RASs with >100 resistance fold change.

	Amino acid position	HCV-1a			HCV-3a		
		Cons.^a^	RAS^b^	RAS > 100X^c^	Cons.^a^	RAS^b^	RAS > 100X^c^
NS5A RASs	28	M	A, G, T	A, G, T	M	–	–
	30	Q	D, E, G, H, K, N, R, T, Y	D, E, G, H, K, N, R, Y	A	K	–
	31	L	F, I, M, V	F, I, M, V	L	F, I, M, V	F, I, M, V
	32	P	L	L	P	–	–
	58	H	D	D	P	–	–
	93	Y	C, H, N, R, S, T, W	C, H, N, R, S, W	Y	H	H
NS5B RASs	282	S	T	–	S	T	–

*^*a*^Consensus amino acid was selected based on the alignment of the included sequences by each HCV genotype. ^*b*^Amino acid substitutions conferring > 2 resistance fold change (GENAFOR, 2019). ^*c*^Amino acid substitutions conferring > 100 resistance fold change (Sorbo et al., 2018). Abbreviations: Cons., consensus; RAS, resistance-associated substitution; A, alanine; G, glycine; T, threonine; V, valine; C, cysteine; E, glutamate; H, histidine; I, isoleucine; K, lysine; R, arginine; S, serine; Y, tyrosine; F, phenylalanine; M, methionine; D, aspartate; N, asparagine; W, tryptophan; L, leucine; Q, glutamine; and P, proline.*

### Phylogenetic Analyses

The sequences of HCV-1a and -3a obtained from patients with HCV infection, as well as HCV-1a and -3a reference sequences from the NCBI nucleotide database, were used to build phylogenetic trees for each HCV genotype, separately. Phylogenetic trees have been constructed automatically using the Kimura 2-parameter approach implemented in the statistical method Neighbor-Joining and bootstrap values were calculated using 1000 bootstrap iterations.

### Statistical Analysis

Data were analyzed using SPSS version (25.0.0.0. Chicago, IL, United States). The *P* < 0.05 was considered statistically significant.

## Results

### Baseline Characteristics of Study Population

Both target regions (NS5A and NS5B) of HCV were successfully amplified in 72/72 and 63/63 patients who were infected with HCV-1a and -3a, respectively. Baseline demographic characteristics of 72 patients with HCV-1a infection and 63 with HCV-3a infection have been summarized in Table 2. The mean age was 41.6 ± 8.7 and 43.4 ± 9.2 years for HCV-1a, and -3a groups, respectively. In the HCV-1a group, 42/72 (58.3%) were males, and 30/72 (41.7%) patients were females. There were 60.3% (38/63) males, and 39.7% (25/63) females in patients with HCV-3a infection. Their mean HCV RNA level was 5.2 ± 3.8 Log IU/ml, and 5.8 ± 4.1 Log IU/mL for HCV-1a and -3a, respectively. The majority (56.9%) of patients in the HCV-1a group had cirrhosis, and 43.1% of patients had no evidence of cirrhosis. In patients with HCV-3a infection, cirrhosis had been diagnosed in 63.5% of them, and 36.5% were non-cirrhotic. All patients were DAA-naïve, however, 44.4% of patients with HCV-1a and 30.2% of patients with HCV-3a had a previous history of IFN-based treatment. Among HCV-1a and -3a patients, 12 (16.7%), and 17 (27%) had cirrhosis and also, a previous history of IFN-based treatment. Data has been depicted in detail in Table 2.

**TABLE 2:** Characteristics of direct-acting antiviral agent-naïve patients with hepatitis C virus infection (genotypes 1a and 3a).

		HCV-1a (*n* = 72)	HCV-3a (*n* = 63)
Sex, *n* (%)	Male	42 (58.3)	38 (60.3)
	Female	30 (41.7)	25 (39.7)
Age (years)	mean ± SD	41.6 ± 8.7	43.4 ± 9.2
HCV RNA level (log IU/mL)	mean ± SD	5.2 ± 3.8	5.8 ± 4.1
Cirrhosis condition, *n* (%)	Non-cirrhotic	31 (43.1)	23 (36.5)
	Cirrhotic	41 (56.9)	40 (63.5)
History of treatment with IFN-based regimens	Naïve	40 (55.6)	44 (69.8)
	IFN-experienced	32 (44.4)	19 (30.2)

*Abbreviations: HCV, hepatitis C virus; IU/mL, international units per milliliter; and IFN, interferon.*

### Prevalence of NS5A Amino Acid Substitutions and RASs

The frequency of identified NS5A amino acid substitutions/RASs in males was higher than in females, however, the difference in both HCV genotypes (1a and 3a) was not statistically significant (*P* > 0.05). In the patients with HCV-1a infection, the prevalence of NS5A amino acid substitutions/RASs was 26.4% (19/72) from which, 12 patients had cirrhosis and 7 patients without cirrhosis. In these patients, the identified NS5A amino acid substitutions/RASs were 11.1% (8/72) in amino acid (aa) 28 (M28T = 2, M28V = 5, and M28I = 1), 4.2% (3/72) of aa 30 (Q30R = 2 and Q30H = 1), 1.4% (1/72) of aa 31 (L31M), and 16.7% (12/72) of aa 58 (H58Y = 2, H58C = 2, H58D = 2, H58P = 2, H58Q = 1, and H58S/T/C = 3). In HCV-1a sequences, the RASs > 100X were identified in 8.3% (6/72) patients including M28T (2 patients), Q30R and Q30H (2 and 1 patients, respectively), and L31M (1 patient). From these six clinically relevant RASs > 100X, five (M28T in 1 patient, Q30R in 2, Q30H in 1, and L31M in 1) were identified in patients with cirrhosis that had a previous history of treatment with IFN-based regimens, and one (M28T) was detected in a non-cirrhotic patient (*P* < 0.05). None of the HCV-1a NS5A sequences harbored the Y93H substitution. Paired NS5A amino acid substitutions were identified in 5.6% (4/72) patients (each in 1 patient) including M28V+H58S/T/C, L31M+H58C, M28V+Q30R, and M28T+Q30H. The results of detected RASs in HCV-1a NS5A have been shown in Table 3.

**TABLE 3:** Baseline NS5A amino acid substitutions/resistance-associated substitutions in patients with HCV infection.

HCV genotype	RASs, Position/amino acid	Number and proportion	Paired RASs, Position/amino acid	Total number and proportion of paired RASs
1a	M28T/V/I	8/72 (11.1%)	M28V+H58S/T/C	1/72 (1.4%)
	Q30R/H	3/72 (4.2%)	M28V+Q30R	1/72 (1.4%)
	L31M	1/72 (1.4%)	M28T+Q30H	1/72 (1.4%)
	H58Y/P/C/D/Q/S/T	12/72 (16.7%)	L31M+H58C	1/72 (1.4%)
3a	A30T/K	6/63 (9.5%)	L31F+P58S/T/C+Y93H	1/63 (1.6%)
	L31F	1/63 (1.6%)	A30T/K+P58S/T/C	1/63 (1.6%)
	P58S/T/C	2/63 (3.2%)		
	Y93H/N	4/63 (6.3%)		

In patients with HCV-3a infection, the number of patients harboring known NS5A amino acid substitutions/RASs at baseline was 10 out of 63 patients (15.9%) which 7 patients had cirrhosis and previous history of IFN-based treatment, and the remaining 3 patients were non-cirrhotic with previous history IFN-based treatment. Six (9.5%) amino acid substitutions/RASs were identified in aa 30 (A30T/K), 1 (1.6%) in aa 31 (L31F), 2 (3.2%) in aa 58 (P58S/T/C), 4 (6.3%) in aa 93 including 2 (3.2%) Y93H, and 2 (3.2%) Y93N. From these amino acid substitutions, 3/63 (4.8%) were identified as RASs; 1 as RAS L31F and 2 as RAS Y93H. In patients with HCV-3a infection, multiple amino acid variants in a single position were detected totally in 6.3% (4/63) of patients including A30T/K in 2 patients, and P58S/T/C in 2 patients. The clinically important substitution (RAS > 100X) Y93H was detected in 2 (3.2%) patients with cirrhosis and previous history of IFN-based treatment. Paired NS5A amino acid substitutions/RASs were identified in 2 (3.2%) patients with cirrhosis: L31F+P58S/T/C+Y93H and A30T/K+P58S/T/C each in one patient. Table 3 has presented the results of identified NS5A RASs in HCV-3a in this study.

### Prevalence of NS5B RASs

In this study, those NS5B RASs conferring resistance to nucleotide analog inhibitors (Sofosbuvir) were not identified.

### Phylogenetic Analysis

**FIGURE 1:**
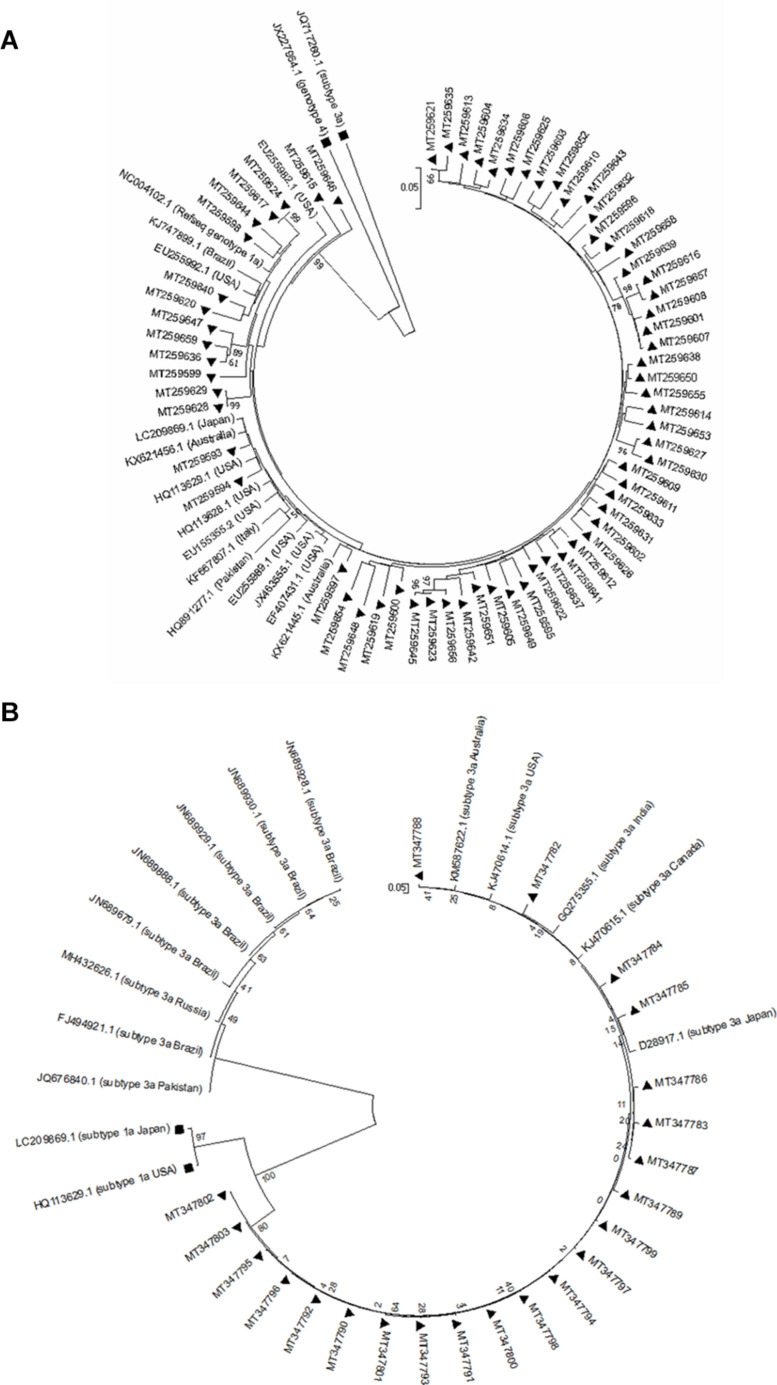
Phylogenetic trees of NS5A sequences of HCV genotype 1a **(A)** and 3a **(B)**. The phylogenetic tree was constructed automatically by applying the Kimura 2 parameter approach implemented in Neighbor-Joining, and bootstrap values were calculated using 1,000 bootstrap iterations. The vertical scale bar represents 0.05 nucleotide substitutions per site. Reference sequences of HCV genotypes 3a and 4 were used as outgroups in **(A)** that have been shown with black squares, and reference sequences of HCV genotypes 1a were used as outgroups in **(B)** that have been indicated by black squares. Sequences from the study are presented by their GenBank accession numbers indicated by black triangles.

**FIGURE 2:**
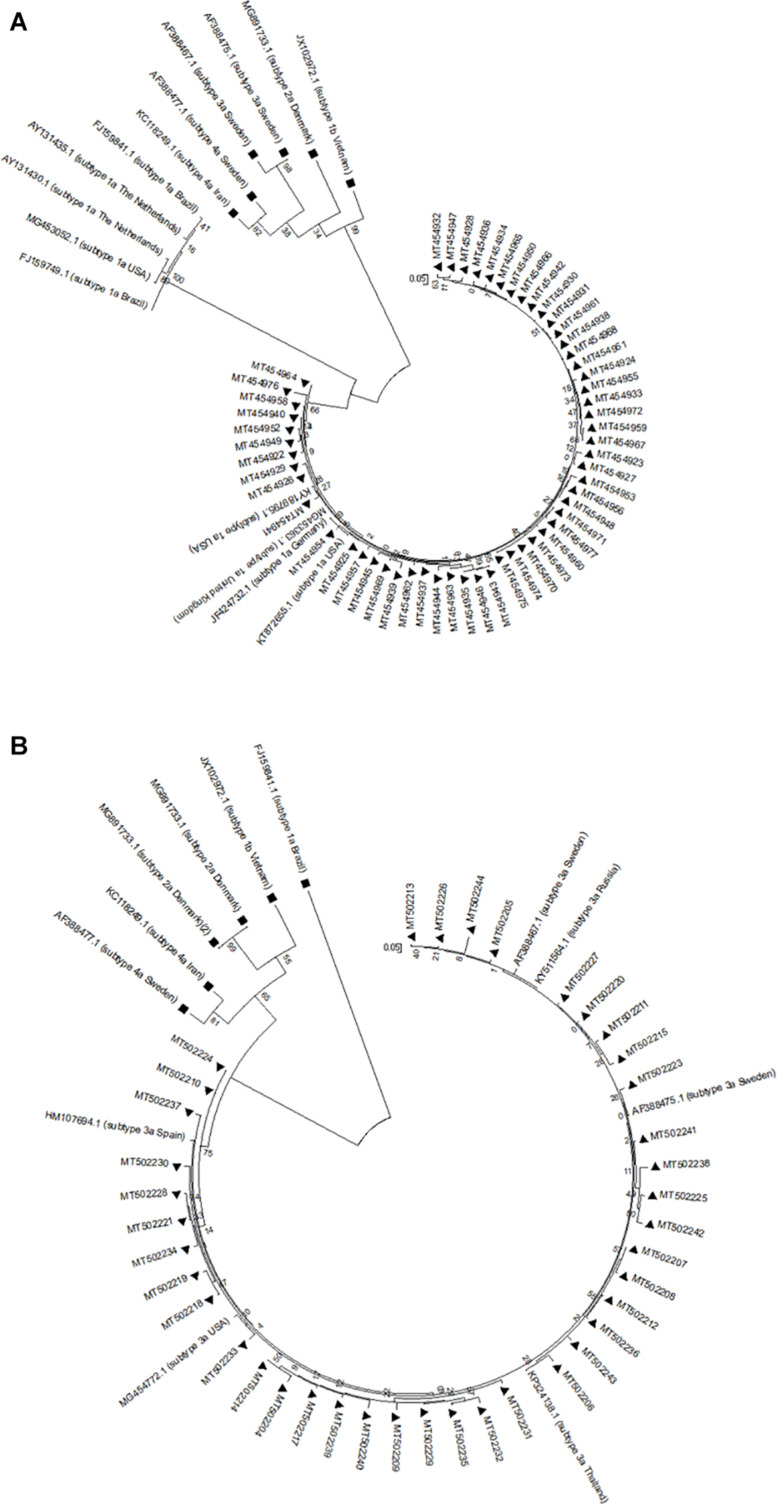
Phylogenetic trees of NS5B sequences of HCV genotype 1a **(A)** and 3a **(B)**. The phylogenetic tree was constructed automatically by applying the Kimura 2 parameter approach implemented in Neighbor-Joining, and bootstrap values were calculated using 1,000 bootstrap iterations. The vertical scale bar represents 0.05 nucleotide substitutions per site. Reference sequences of HCV genotypes 1b, 2a, 3a, and 4a were used as outgroups in **(A)** that have been shown with black squares, and reference sequences of HCV genotypes 1a, 1b, 2a, and 4a were used as outgroups in **(B)** that have been indicated by black squares. Sequences from this study are presented by their GenBank accession numbers indicated by black triangles.

The genetic relationship among NS5A and NS5B sequences from each HCV-1a and -3a was investigated through the phylogenetic analysis. The results for NS5A phylogenetic analysis of HCV-1a and -3a have been shown in Figures 1A,B, respectively. The results for NS5B phylogenetic analysis of HCV-1a and -3a have been shown in Figures 2A,B, respectively. In all four phylogenetic trees, sequences of the same region and the same subtype were grouped closely, while considerable distance was found between those sequences and outgroups in each tree that confirm the accuracy of sequences from NS5A and NS5B regions of each HCV-1a and -3a patients. According to the phylogenetic trees, the HCV subtypes were the same as the HCV genotyping results included in the patients’ clinical records. Briefly, the consistency between the HCV genotypes (1a and 3a) and the results of NS5A and NS5B phylogenetic analyses confirmed that no recombination event occurred between these regions.

## Discussion

Treatment of HCV has been developed substantially after approval of the first generation of DAAs by the United States Food and Drug Administration in 2011 (Ghany et al., 2019, 2020). These years, combinations of different DAAs have been used to treat HCV infection. They are highly efficacious and have fewer side effects with shorter treatment duration (usually 12 weeks), and are better tolerated than previous therapies (Hernandez et al., 2013; Kalaghatgi et al., 2016; Alavian and Sharafi, 2017; GENAFOR, 2019; Ghany et al., 2019, 2020). Although expectancy to achieve SVR even in patients with chronic HCV infection with failure to previous HCV antiviral therapy by using these new DAAs has been dramatically increased, RASs may emerge either before treatment with DAAs or following drug exposure (Fridell et al., 2011; Hernandez et al., 2013; Walker et al., 2015; Paolucci et al., 2017; Zeuzem et al., 2017; World Health Organization, 2017; Grandal et al., 2018; Sharafi et al., 2019). These drug resistance substitutions especially those to NS5A inhibitors lead to treatment failure so, they are considered a major challenge for HCV treatment and elimination (Fridell et al., 2011; Hernandez et al., 2013; Walker et al., 2015; Paolucci et al., 2017; Zeuzem et al., 2017; World Health Organization, 2017; Grandal et al., 2018; Sharafi et al., 2019).

Combinations of DCV/SOF and LDV/SOF are the most preferred regimens with above 95% cure rates and are strongly recommended by WHO (World Health Organization, 2017). Although NS5A inhibitors are effective in low concentrations, there are concerns about the existence of a low barrier to the selection of resistance mutations that decrease susceptibility to NS5A inhibitors. Generally, the NS5A RASs could result in >2 resistance fold-change, and some of them which cause >100 resistance fold-change are known as RASs > 100X (Issur and Götte, 2014; Sharafi and Alavian, 2018). For instance, just a single nucleotide substitution in codon 93 can change its related amino acid (from Y to H, C, and N) in NS5A protein and leads to resistance to most of the NS5A inhibitors (Issur and Götte, 2014; Nakamoto et al., 2014; Sharafi and Alavian, 2018). However, the viral genotype and subtype should be considered as an important factor to define the fold-change (Wang et al., 2013; Issur and Götte, 2014; Nakamoto et al., 2014; World Health Organization, 2017; Brandão et al., 2018; Hezode et al., 2018; Sharafi and Alavian, 2018; Ghany et al., 2019, 2020). In this study, baseline RASs were investigated in 72 HCV-1a patients, and 63 HCV-3a patients, which are the most prevalent HCV genotypes in Iran and were used to be known as “difficult-to-treat genotypes” (Bittar et al., 2010; Fridell et al., 2011; Hernandez et al., 2013; Walker et al., 2015; de Rueda et al., 2017; Paolucci et al., 2017; Zeuzem et al., 2017; Grandal et al., 2018). In our study, all of the patients were DAA-naïve. However, 44.4% of patients with HCV-1a infection, and 30.2% of patients with HCV-3a infection had a history of treatment with IFN-based regimens. There was a total of 41 (56.9%) and 40 (63.5%) cirrhotic patients infected with HCV-1a and HCV-3a, respectively. In cirrhotic patients with HCV-1a infection, 12 (16.7%) had a previous history of IFN-based treatment while it was 17 (27%) in cirrhotic patients with HCV-3a infection. Although the high rate of SVR (>90%) has led to a reduction in the number of studies on baseline resistance before initiating DAA-based treatments, many investigations of baseline RASs showed the impact of NS5A RASs on the virologic outcome for NS5A inhibitor-containing regimens such as DCV/SOF, and LDV/SOF (Wang et al., 2013; Brandão et al., 2018; Sharafi and Alavian, 2018; Sharafi et al., 2019). The European Association for the Study of Liver Diseases (EASL) recommended that the identification of baseline RASs to DAAs including LDV/SOF and DCV/SOF for HCV genotypes such as 1a could be useful to decide on extending treatment duration or if needed adding RBV (Pawlotsky et al., 2020). The techniques which have been used for sequencings such as Sanger sequencing or deep sequencing are an important factor that affects the detection rate of RASs (Ghany et al., 2019, 2020; Pawlotsky et al., 2020). In this study, we could not perform/order deep sequencing because of budget limitations that we were challenged with during this project. Despite that limitation, both NS5A and NS5B regions of HCV-1a and -3a were successfully amplified and Sanger sequencing of 270 PCR products resulted in reliable and accurate detection of amino acid substitutions/RASs as were presented in details in the “Results” section. Many investigators suggested that both methods can be considered equivalent if a ≥15% cut point is used for determination of RASs by NGS due to recent studies that have shown the results of NGS at a 1% level of sensitivity often lead to the identification of additional RASs that are not associated with clinical failure (Zeuzem et al., 2017; Perales et al., 2018; GENAFOR, 2019; Ghany et al., 2019, 2020; Chen et al., 2020). Herein, all the amplified NS5A and NS5B fragments were sequenced using the Sanger method, and NS5A amino acid substitutions/RASs were identified in 26.4%, and 15.9% of patients infected with HCV-1a and -3a, respectively. For instance, in one study, NS5A RASs were detected in 28.4% of Portuguese patients (Brandão et al., 2018; Hezode et al., 2018). Besides, several other studies showed that the overall proportion of NS5A RASs in baseline varies between <10% to >50% (Issur and Götte, 2014). Therefore, the detection rate of NS5A amino acid substitutions/RASs in this study is in line with those studies (Fridell et al., 2011; Hernandez et al., 2013; Paolucci et al., 2013, 2017; Dietz et al., 2015; de Rueda et al., 2017; Wyles and Luetkemeyer, 2017; Brandão et al., 2018; Grandal et al., 2018; Hezode et al., 2018; Ghany et al., 2019, 2020; Sharafi et al., 2019).

The genotype/subtype of HCV is one of the most effective factors, which should be considered in studying baseline RASs (Fridell et al., 2011; Paolucci et al., 2013, 2017; Wyles and Luetkemeyer, 2017). In addition, according to several studies the prevalence of NS5A amino acid substitutions/RASs in patients with HCV-1a was found at a higher rate than in those with HCV-3a infection, and in this study, the prevalence of NS5A amino acid substitutions and also the RASs > 100X in patients infected with HCV-1a was higher than patients with HCV-3a infection (26.4% vs. 15.9% and 8.3% vs. 4.8%) which was concordant with other studies (Sarrazin and Zeuzem, 2010; Nakamoto et al., 2014; Walker et al., 2015; Bagaglio et al., 2016; Kliemann et al., 2016; Pawlotsky, 2016; Malta et al., 2017; McPhee et al., 2017; Zeuzem et al., 2017; Grandal et al., 2018; Lu et al., 2019). Cirrhosis is considered a complicated condition in patients with chronic HCV infection as it indicates persistent viral infection accompanied by virus replication for a long time that could result in viral fitness (Nakamoto et al., 2014; Sarrazin, 2016; Bartolini et al., 2017; Pérez et al., 2017; Welzel et al., 2017; Zeuzem et al., 2017; Smith et al., 2019). In this study, five clinically relevant RASs > 100X, out of six identified RASs > 100X in HCV-1a infected patients were detected in patients with cirrhosis and previous history of IFN-based treatment. In patients with HCV-3a infection, the amino acid substitutions/RASs have detected in 10 patients, seven were cirrhotic with a previous history of IFN-based treatment, and the RAS > 100X Y93H was identified in 2 patients with cirrhosis. Our findings are in accordance with other studies that amino acid substitutions/RASs and especially the clinically relevant RASs (RASs > 100X) could be identified more frequently in cirrhotic patients than in non-cirrhotic patients (Nakamoto et al., 2014; Walker et al., 2015; Sarrazin, 2016; Malta et al., 2017; Paolucci et al., 2017; Welzel et al., 2017; Zeuzem et al., 2017; Brandão et al., 2018; Ghany et al., 2019, 2020).

In patients with HCV-1a infection, substitutions in aa 58 were the most commonly identified substitutions (16.7%) while; substitutions in aa 30 were the most commonly detected amino acid changes in HCV-3a (9.5%) infection and this was consistent with other studies (Sarrazin and Zeuzem, 2010; Hernandez et al., 2013; de Rueda et al., 2017; Ghany et al., 2019, 2020; Smith et al., 2019). Here, we detected substitutions of aa 58 in 12 patients with HCV-1a infection. Although the impact of substitutions in aa 58 is not clear, H58D is known as a resistance substitution and some studies reported that H58P confers the resistance to DCV (Bagaglio et al., 2016; Pawlotsky, 2016; Bartolini et al., 2017; Malta et al., 2017; Lu et al., 2019). Several studies found that when substitution in aa 58 was the only detected RAS, a very low inhibitory effect on resistance to NS5A inhibitors would be expected (Nakamoto et al., 2014; Bagaglio et al., 2016; Pawlotsky, 2016; Bartolini et al., 2017; Malta et al., 2017; Lu et al., 2019). In contrast, a combination of substitutions in aa 58 with other certain NS5A RASs (e.g., L31M, Q30R) could increase the resistance to NS5A inhibitors in patients with HCV-1a infection (Nakamoto et al., 2014; Bagaglio et al., 2016; Bartolini et al., 2017; Malta et al., 2017; Pérez et al., 2017; Welzel et al., 2017). In this investigation, only one combination of substitution in aa 58 with substitution in aa 28 was identified in HCV-1a; (M28V+H58S/T/C). A similar combination of substitutions in aa 58 was found in two patients with HCV-3a infection: (A30T/K+P58S/T/C) and (L31F+P58S/T/C+Y93H).

Multiple amino acid variations in specific locations such as aa 58 and aa 30 in NS5A of HCV-1a and HCV-3a have been identified with different frequencies in many studies (Bittar et al., 2010; Paolucci et al., 2013; Issur and Götte, 2014; Bagaglio et al., 2016; Kliemann et al., 2016; Pawlotsky, 2016; Malta et al., 2017; McPhee et al., 2017; Lu et al., 2019). Herein, the multiple amino acid changes were found in 4.2% of HCV-1a patients in aa 58 (H58S/T/C), and in 6.3% of HCV-3a sequences in aa 30 (A30T/K), and aa 58 (P58S/T/C). The presence of NS5A RASs at baseline has been known effective on the outcome of DAA-based treatments while each RAS has a specific impact on its DAA target. For example, Q30R/H and M28T have been considered as low-level resistance substitutions for HCV-1a by some researchers (Nakamoto et al., 2014; Bagaglio et al., 2016; Pawlotsky, 2016; Bartolini et al., 2017; Malta et al., 2017). In this study, Q30R/H and M28T/V/I substitutions were identified in 4.2% and 11.1% of patients with HCV-1a infection, respectively.

Generally, the presence of pre-treatment NS5A RASs has a negative effect on achieving SVR in comparison to those without baseline NS5A RASs (Bagaglio et al., 2016; Welzel et al., 2017). In one study, SVR in patients with baseline NS5A RASs has been estimated at 93.5%, while it was 98.4% in patients without baseline NS5A RASs (Sarrazin et al., 2016). Moreover, an increase in SVR failure could be observed when the NS5A RASs > 100-fold resistance exist (Bagaglio et al., 2016; de Rueda et al., 2017; Welzel et al., 2017; Sharafi and Alavian, 2018; Smith et al., 2019). In our study, the clinically significant NS5A RASs > 100X of genotype 1a were detected in 8.3% of patients (Q30R/H:3 patients, L31M:1 patient, and M28T:2 patients), without any NS5A RASs in aa 93 while, 3.2% of patients with HCV-3a infection harbored this substitution (Y93H:2 patients). Several studies reported that only Y93H should be considered as clinically significant NS5A RAS > 100X in HCV-3a (Nakamoto et al., 2014; Bagaglio et al., 2016; Kalaghatgi et al., 2016; Paolucci et al., 2017; Dietz et al., 2018; Perales et al., 2018; Smith et al., 2019). Herein, one of the identified substitution Y93H was paired with RASs in aa 31, and aa 58 (L31F+P58S/T/C+Y93H). According to some studies, RASs in aa 93 (Y93H) is considered a > 100 resistance fold-change NS5A RAS, especially when it appeared as paired substitution with RASs in aa 31 in HCV-3a (Wang et al., 2013; Nakamoto et al., 2014; Paolucci et al., 2017; Dietz et al., 2018; Smith et al., 2019).

Although in some reports the rate of naturally-occurring > 100 resistance fold-change NS5A RASs in treatment-naïve patients has been estimated as less than 5%, other studies have shown the contrary results as in one study there were 14.6% NS5A RASs > 100X for HCV-1a, and 22.6% for HCV-3a (Sarrazin et al., 2016; Malta et al., 2017; Welzel et al., 2017; Zeuzem et al., 2017; Sharafi and Alavian, 2018). In another study, the rate of baseline NS5A RASs > 100X was reported as 9.9% (Hezode et al., 2018). This controversy might result from variation in the number of studied patients, genetic factors such as *IFNL3/4* polymorphisms, the difference in the prevalence of HCV genotypes in the area where the study was done, and the viral load (Nakamoto et al., 2014; Bagaglio et al., 2016; Pawlotsky, 2016; Bartolini et al., 2017; Malta et al., 2017).

Liver status such as cirrhosis is another important predictor of treatment response (Pawlotsky, 2016; Bartolini et al., 2017; Malta et al., 2017; Pérez et al., 2017; Welzel et al., 2017; Smith et al., 2019). Herein, 56.9% of patients with HCV-1a infection, and 63.9% of patients with HCV-3a infection were diagnosed with cirrhosis. In patients infected with HCV-1a, the clinically relevant substitutions (RASs > 100X) were detected in 6/72 patients, from them five substitutions were identified in cirrhosis: M28T in one patient, Q30R/H in 3 patients, and L31M in 1 patient. While one of the RAS > 100X (M28T) was detected in one non-cirrhotic HCV-1a patient. The clinically important substitution (RAS > 100X) Y93H was detected in 3.2% (2/63) of patients with HCV-3a infection, both diagnosed with cirrhosis. In addition, the identified two paired NS5A amino acid substitutions/RASs (L31F+P58S/T/C+Y93H) and (A30T/K+P58S/T/C) were detected each in one cirrhotic HCV-3 patient. In both HCV-1a and HCV-3a infected patients in this study, the high prevalence of RASs > 100X in cirrhotic patients in comparison with non-cirrhotic patients has been reported in many studies, and here our results conformed with them (Sulkowski et al., 2014; Bagaglio et al., 2016; Sarrazin et al., 2016; Bartolini et al., 2017; Malta et al., 2017; McPhee et al., 2017; Paolucci et al., 2017; Lu et al., 2019; Sayan et al., 2020).

Some investigators proposed that the SVR rate in patients infected with HCV-1a or -3a, could be strongly affected by the presence of RASs in companion with a history of previous IFN-based treatment, and this negative effect was irrespective of the cirrhosis status (Walker et al., 2015; Sarrazin, 2016; Sarrazin et al., 2016; Welzel et al., 2017; Zeuzem et al., 2017; Dietz et al., 2018; Esposito et al., 2019; Smith et al., 2019). However, many other researchers believed that achieving SVR is a multifactorial event that could be affected by host factors such as liver conditions (cirrhosis), the previous history of IFN-based treatment, the existence of some *IFNL3/4* polymorphisms, and consuming alcohol. Also, viral factors including infection with some genotypes (1a and 3), the presence of RASs, HIV/HCV co-infection, and viral load could affect the treatment response in a negative way. However, treatment strategy including its antiviral drugs and treatment duration could overcome other factors causing poor prognosis (Sarrazin and Zeuzem, 2010; Sarrazin, 2016; Sarrazin et al., 2016; Bartolini et al., 2017; Paolucci et al., 2017; Pérez et al., 2017; Zeuzem et al., 2017; Aldunate et al., 2018; Dietz et al., 2018; Esposito et al., 2019; Sayan et al., 2020). Sofosbuvir (PSI-7977 and GS-7977), is a very potent viral RdRp (NS5B) inhibitor with a pan-genotypic effect on HCV, without issues of viral resistance (Murakami et al., 2010; Sofia et al., 2010; Poordad and Dieterich, 2012; Stedman, 2014; Tong et al., 2014; Costantino et al., 2015; Gallego et al., 2016; Gane et al., 2017; Cory et al., 2018; Sorbo et al., 2018). It is a prodrug of 2′-deoxy-2′-fluoro-2′-C-methyluridine monophosphate which should be changed to its activated form (2‘- C-methyl group) in the liver and acts as a chain terminator (Murakami et al., 2010; Sofia et al., 2010; Poordad and Dieterich, 2012; Lawitz et al., 2014; Stedman, 2014; Tong et al., 2014; Costantino et al., 2015; Gallego et al., 2016; Bagaglio et al., 2018; Cory et al., 2018; Sorbo et al., 2018). Since its discovery, SOF has dramatically changed the treatment outcome of patients with HCV infection who were infected with hard-to-treat genotypes (1 and 3), cirrhosis, and previous history of treatment (Sofia et al., 2010; Lawitz et al., 2014; Stedman, 2014; Gallego et al., 2016; Gane et al., 2017). Despite being mutation prone which results in the selection of most fitted variants, especially when virus replication is under pressure due to anti-HCV drugs, SOF has shown the exception results as SOF-resistant variants may not be selected or even selectable (Sofia et al., 2010; Poordad and Dieterich, 2012; Lawitz et al., 2014; Tong et al., 2014; Costantino et al., 2015; Gallego et al., 2016; Gane et al., 2017; McPhee et al., 2017; Bagaglio et al., 2018; Sorbo et al., 2018).

## Conclusion

In this study, NS5A RASs were identified at baseline in Iranian patients with HCV-1a and -3a infection. The prevalence of baseline RASs was in line with similar reports from other countries. Recently, new drugs with a high genetic barrier to resistance have been introduced to the pipeline as a combination of pan-genotypic regimens. As a result, promising anti-HCV treatment and even, elimination of HCV infection could be achieved.

## Data Availability Statement

The dataset is available upon request from PR (pooneh5376@yahoo.com). The submitted sequences in GenBank are available as a Supplementary Material.

## Ethics Statement

The studies involving human participants were reviewed and approved by Ethics Committee of the Pasteur Institute of Iran (no: IR.PII.REC.1395.81). The patients/participants provided written informed consent to participate in this study.

## Author Contributions

PR designed the research. PR, HS, GB, and SA analyzed results. PR and HS drafted the manuscript. FS, NA, and VI contributed to the clinical data collection and laboratory detection methods. All authors read and approved the submitted version.

## Conflict of Interest

The authors declare that the research was conducted in the absence of any commercial or financial relationships that could be construed as a potential conflict of interest.
