# Supplementary material for: Prevalence of Naturally-Occurring NS5A and NS5B Resistance-Associated Substitutions in Iranian Patients With Chronic Hepatitis C Infection
Source: Front Microbiol. 2021 Jan 28;11:617375. doi: 10.3389/fmicb.2020.617375 (PMC7876467; doi:10.3389/fmicb.2020.617375)
Supplement: Supplementary file 1 [file Data_Sheet_1.docx]

>MT259593

TCCGGTTCCTGGCTGAGGGACATCTGGGACTGGATATGCGAGGTGCTGAGCGATTTTAAG

ACCTGGCTGAAGGCCAAGCTCATGCCACAACTGCCTGGGATTCCCTTTGTGTCCTGCCAA

CGCGGATATAGGGGGGTCTGGCGAGGAGATGGCATTATGCACACTCGCTGCCACTGTGGA

GCCGAGATCACTGGACATGTCAAGAACGGGACGATGAGGATCGTCGGTCCTAAGACCTGC

AGGAACATGTGGAGTGGAACCTTCCCCATCAACGCCTATACCACGGGCCCCTGTACCCCC

CTT

>MT259594

TCCGGTTCCTGGCTAAGGGACATCTGGGACTGGATATGCGAGGTGCTGAGCGACTTCAAG

ACCTGGCTGAAGGCCAAGCTCATGCCACAACTGCCTGGGATTCCCCTTGTGTCCTGCCAA

CGCGGATATAGGGGGGTCTGGCGAGGGGATGGCATTATGTACACTCGCTGCCACTGTGGA

GCTGAGATTACTGGACATGTCAAGAACGGGACGATGAGGATCGTCGGTCCTAAGACCTGC

AGGAACATGTGGAGTGGGACCTTCCCCATCAACGCCTACACCACGGGCCCCTGTACCCCC

CTT

>MT259595

TCCGGTTCCTGGCTGAGGGACGTCTGGGACTGGATATGTGAGGTGCTGAGCGATTTTAAG

ACCTGGCTGAAGGCCAAGCTCATGCCACAACTGCCCGGGATTCCCTTTGTGTCCTGTCAA

CGCGGGTATAGGGGGGTCTGGCGGGGAGATGGCATTATGCACACCCGCTGCCATTGTGGA

GCTGAGATCACTGGGCATGTCAAGAACGGGACGATGAGGATCGTCGGTCCTAAGACCTGC

AGGAACATGTGGAGTGGGACCTTCCCCATCAACGCCTATACCACAGGCCCCTGCACCCCC

CTT

>MT259596

TCCGGTTCCTGGTTAAGGGACATCTGGGACTGGATATGCGAGGTGCTGAGCGATTTTAAG

ACCTGGCTGAAGGCCAAGCTCATGCCACAACTGCCCGGGATTCCCTTTATGTCCTGCCAA

CGCGGGTATAAGGGGGTCTGGCGGGGAGATGGTATTATGCACACTCGCTGCCATTGTGGA

GCTGAGATCACTGGACATGTCAAGAACGGGACGATGAGGATCGTCGGTCCTAAGACCTGC

AGGAACATGTGGAGTGGAACCTTCCCCATCAACGCCTACACCACAGGCCCCTGCACCCCC

CTT

>MT259597

TCCGGTTCCTGGCTAAGGGACATCTGGGACTGGATATGCGAGGTGCTGAGCGATTTCAAG

ACCTGGCTGAAGGCCAAGCTCATGCCACAGCTGCCTGGGATTCCCTTTGTGTCCTGCCAA

CGCGGGTATAGGGGGGTCTGGCGAGGAGATGGCATTATGCACACCCGCTGCCATTGTGGA

GCTGATATCACTGGACATGTCAAGAACGGGACGATGAGGATCGTCGGTCCTAAGACCTGC

AGGAACATGTGGAGTGGGACCTTCCCCATCAACGCCTACACCACGGGCCCCTGTACTCCC

CTT

>MT259598

TCGGGCTCGTGGCTAAGGGACATCTGGGACTGGATATGCGAGGTGATAAGCGACTTCAAG

ACCTGGCTGAAAGCCAAGCTCATGCCGCAACTGCCCGGGATTCCCTTCGTGTCCTGCCAG

CGCGGGTATAGGGGGGTCTGGCGAGGAGACGGCATTATGCACACTCGCTGCCACTGTGGA

GCTGAGATCACTGGCCATGTCAAAAACGGGACGATGAGGATCGTCGGTCCTAAGACCTGC

AGGAACATGTGGAGTGGGACTTTCCCCATTAACGCCTACACCACGGGCCCCTGTACTCCC

CTT

>MT259599

TCCGGTTCCTGGTTGAGGGACATCTGGGACTGGATATGCGAGGTGCTGAGCGACTTTAAG

ACCTGGCTGAAAGCCAAGCTCATGCCACAACTGCCTGGTATTCCCTTTGTATCCTGCCAG

CGTGGGTATAGGGGGGTCTGGCGAGGGGACGGCATCATGCACACTCGCTGCCACTGTGGA

GCTGAGATCACTGGACATGTCAAAAACGGGACGATGAGGATCGTCGGCCCTAAGACCTGC

AGGAACATGTGGAGTGGGACCTTCCCCATCAACGCCTACACTACGGGCCCCTGCACCCCA

CTT

>MT259600

TCCGGTTCCTGGTTAAGAGACATCTGGGACTGGATATGCGAGGTGCTGAGCGATTTCAAG

ACCTGGCTGAAGGCCAAGCTCATGCCACAACTGCCCGGGATTCCCTTTGTGTCCTGCCAA

CGCGGGTATAGGGGGGTCTGGCGGGGAGATGGTATTATGCACACTCGCTGCCACTGTGGA

GCTGAGATCACTGGACATGTCAAGAACGGGACGATGAGGATCGTCGGTCCTAGGACCTGC

AGAAACATGTGGAGTGGGACCTTCCCCATCAACGCCTACACCACGGGCCCCTGCACTCCC

CTT

>MT259601

TCCGGCTCCTGGTTAAGGGACATCTGGGACTGGATATGCGAGGTGCTGAGCGATTTTAAG

ACCTGGCTGAAGGCCAAGCTCATGCCACAACTGCCCGGGATTCCCTTTGTATCCTGCCAA

CGCGGGTATAAGGGGGTCTGGCGGGGAGATGGTATTATGCACACTCGCTGCCATTGTGGA

GCTGAGATCACTGGACATGTCAAAAACGGGACGATGAGGATCGCCGGTCCTAGGACCTGC

AGGAACATGTGGAGTGGAACCTTCCCCATCAACGCCTACACCACGGGCCCCTGCACCCCC

CTT

>MT259602

TCCGGTTCCTGGTTAAGGGACATCTGGGACTGGATATGCGAGGTGCTGAGCGATTTTAAG

ACCTGGCTGAAGGCCAAGCTCATGCCACAACTGCCTGGAATTCCCTTTGTGTCCTGCCAA

CGCGGGTATAGGGGGGTCTGGCGAGGAGATGGTATAATGCATACTCGCTGCCATTGTGGA

GCTGAGATCACTGGACATGTCAAGAACGGGACGATGAGGATCGTTGGTCCCAAGACCTGC

AGGAACATGTGGAGTGGGACCTTCCCCATCAACGCCTACACCACGGGCCCCTGTACCCCC

CTT

>MT259603

TCCGGTTCCTGGTTAAGGGACGTTTGGGACTGGATATGCGAGGTGCTGAGCGATTTTAAG

ACCTGGCTGAAGGCCAAGCTCATGCCACAACTGCCCGGGATTCCCTTTGTGTCCTGCCAA

CGCGGGTATAGGGGGGTCTGGCGGGGAGATGGTATTATGCACACTCGCTGCCATTGTGGG

GCTGAGATCACCGGACATGTCAAGAACGGGACGATGAGGATCGTCGGTCCCAAGACCTGC

AGGAACATGTGGAGTGGGACCTTCCCCATCAACGCCTACACCACGGGCCCTAGCACCCCC

CTT

>MT259604

TCCGGTTCCTGGTTAAGGGACATCTGGGACTGGATATGTGAGGTGCTGAGCGATTTTAAG

ACCTGGCTGAAGGCCAAGCTCATGCCACAACTGCCCGGGATTCCCTTTGTGTCCTGCCAA

CGCGGGTATAGGGGGGCCTGGCGAGGAGATGGTATCATGCACACTCGCTGCCATTGTGGA

GCTGAAATTACCGGGCATGTCAAGAACGGGACGATGAGGATCGTCGGTCCTAAGACCTGC

AGGAACATGTGGAGTGGGACCTTCCCCATCAACGCCTACACCACGGGCCCCTGCAGCCCC

CTT

>MT259605

TCCGGTTCCTGGTTAAGGGACATCTGGGACTGGATATGCGAGGTGCTGAGCGATTTTAAG

ACCTGGCTAAAGGCCAAGCTCGTGCCACAACTGCCCGGGATTCCCTTTGTGTCCTGCCAA

CGCGGGTATAGGGGGGTCTGGCGGGGGGATGGCATTATGCACACTCGCTGCCATTGTGGA

GCTGAGATCACTGGACATGTCAAGAACGGGTCGATGAGGATCGTCGGTCCTAAGACTTGT

AGGAACATGTGGAGTGGGACCTTCCCCATCAACGCCTACACCACGGGCCCCTGCACCCCC

CTT

>MT259606

TCCGGTTCCTGGTTAAGGGACATCTGGGACTGGATATGCGAGGTGCTGAGCGATTTTAAG

ACCTGGCTGAAGGCCAAGCTCATGCCACAACTGCCCGGGATTCCCTTTGTGTCCTGCCAA

CGAGGATATAGGGGGGTCTGGCGGGGAGATGGTATTATGCACACTCGCTGCCATTGTGGA

GCTGAGATTACTGGACATGTCAAGAACGGGACGATGAGGATCGCCGGCCCTAAGACCTGC

AGGAACATGTGGAGTGGGACCTTCCCCATCAACGCCTACACCACGGGGCCTTGCACCCCC

CTT

>MT259607

TCCGGCTCCTGGTTAAGGGACATCTGGGACTGGATATGCGAGGTGCTGAGCGATTTTAAG

ACCTGGCTCAAGGCCAAGCTCATGCCACAACTGCCCGGGATTCCCTTTGTATCCTGCCAA

CGCGGGTATAAGGGGGTCTGGCGGGGAGATGGTATTATGCACACTCGCTGCCATTGTGGA

GCTGAGATCACTGGACATGTCAAAAACGGGACGATGAGGATCGCCGGTCCTAGGACCTGC

AGGAACATGTGGAGTGGAACCTTCCCCATCAACGCCTACACCACGGGCCCCTGCACCCCC

CTT

>MT259608

TCCGGCTCCTGGTTAAGGGACATCTGGGACTGGATATGCGAGGTGCTGAGCGATTTTAAG

ACCTGGCTGAAGGCCAAGCTCATGCCACAACTGCCCGGAATTCCCTTTGTATCCTGCCAA

CGCGGGTATAAGGGGGTCTGGCGGGGAGATGGTATTATGCACACTCGCTGCCATTGTGGG

GCTGAGATTACTGGACATGTCAAAAACGGGACGATGAGGATCGCCGGTCCTAGGACCTGC

AGGAACATGTGGAGTGGAACCTTCCCCATCAACGCCTACACCACGGGCCCCTGCACCCCC

CTT

>MT259609

TCTGGTTCCTGGTTAAGGGACATCTGGGACTGGATATGCGAGGTGCTGAGCGATTTTAAG

ACCTGGCTGAAGGCCAAGCTCATGCCACAACTGCCCGGGATTCCCTTTGTGTCCTGCCAA

CGCGGGTATAGGGGGGCCTGGCGAGGGGATGGTATTATGCACACTCGCTGCCATTGTGGA

GCTGAGATCACTGGACATGTCAAGAACGGGACGATGAGGATCGTCGGTCCTAAGACCTGC

AAGAACATGTGGAGTGGGACCTTCCCCATCAACGCCTACACCACGGGCCCCTGCACCCCC

CTT

>MT259610

TCCGGTTCCTGGTTGAGGGACATCTGGGATTGGATATGCGAGGTGCTGAGCGATTTTAAG

ACCTGGCTGAAGGCCAAGCTCATGCCACAACTGCCCGGGATTCCCTTTGTGTCCTGCCAA

CGCGGGTATAGGGGGGTCTGGCGAGGAGATGGTATTATGCACACCCGCTGCCATTGTGGA

GCTGAAATCACTGGACATGTCAAGAACGGGACGATGAGGATCGTCGGTCCTAAGACCTGC

AGAAACATGTGGAGTGGAACCTTCCCCATCAACGCCTATACCACGGGCCCTTGCACCCCC

CTT

>MT259611

TCCGGTTCCTGGTTAAGGGACATCTGGGACTGGATATGCGAGGTGCTGAGCGATTTTAAG

ACCTGGCTGAAGGCCAAGCTCATGCCACAACTGCCCGGGATTCCCTTTGTGTCCTGCCAA

CGCGGGTATAGGGGGGCCTGGCGAGGAGATGGTATTATGCACACTCGCTGTTGTTGTGGA

GCTGAGATCACTGGACATGTCAAGAACGGGACGATGAGGATCGCCGGTCCTAAGACCTGC

AGGAACATGTGGAGTGGGACCTTCCCCATCAACGCCTATACCACGGGCCCCTGTACCCCC

CTT

>MT259612

TCCGGCTCCTGGTTAAGGGACATCTGGGACTGGATATGCGAGGTGCTGAGCGATTTTAAG

ACCTGGCTGAAAGCCAAGCTCATGCCACAACTGCCAGGGATTCCCTTCGTGTCCTGCCAA

CGCGGGTATAGGGGGGTCTGGCGAGGAGATGGCATTATGCACACTCGCTGCCATTGTGGA

GCTGAAATCACTGGACATGTCAAGAACGGGACGATGAGGATCGTCGGTCCTAAGACCTGC

AGAAACATGTGGAATGGGACCTTCCCCATCAACGCCTACACCACGGGCCCCTGCACCCCC

CTT

>MT259613

TCCGGCTCCTGGTTAAGGGACATCTGGGACTGGATATGCGAGGTGCTAAGCGATTTTAAG

ACCTGGCTGAAGGCCAAGCTCATGCCACAACTGCCCGGGATTCCCTTTGTGTCCTGCCAA

CGCGGGTATAGGGGGGTCTGGCGGGGAGATGGTATCATGCACACTCGCTGCCACTGTGGA

GCTGAGATTACTGGACATGTCAAGAACGGGACGATGAGGATCGTTGGTCCTAAGACTTGC

AAGAACATGTGGAGTGGGACCTTCCCTATCAACGCCTACACCACGGGCCCCTGCACCCCC

CTT

>MT259614

TCCGGTTCCTGGTTAAGGGACATCTGGGACTGGATATGCGAAGTGCTGAGCGACTTTAAG

ACATGGCTGAAGGCCAAGCTCATGCCACAACTGCCCGGGATTCCCTTTGTGTCCTGCCAG

CGCGGGTATAAGGGGGTCTGGCGGGGAGATGGCATTATGCACACTCGCTGCCATTGTGGA

GCTGAGATCACTGGACATGTCAAGAACGGGACGATGAGGATCGTCGGTCCTAAGACCTGC

AAGAACATGTGGAGTGGAACCTTCCCCATCAACGCCTACACCACGGGTCCCTGCACCCCC

CTT

>MT259615

TCCGGCTCCTGGCTAAGGGACGTCTGGGACTGGATATGCGAGGTGCTGAGCGACTTCAAG

ACCTGGCTGAAAGCCAAGCTCATGCCACAACTGCCTGGGATCCCTTTTGTGTCCTGCCAA

CGCGGGTACAGAGGGGTCTGGCGGGGGGATGGCATCATGCACACTCGCTGCCACTGTGGA

GCTGAAATCACTGGACATGTCAAAAACGGGACGATGAGGATCGTCGGCCCCAAGACCTGT

AGGAACATGTGGGGTGGAACCTTCCCCATCAACGCCTACACCACGGGCCCCTGTACCCCC

CTT

>MT259616

TCCGGCTCATGGCTAAGGGACATCTGGGACTGGATATGCGAGGTGCTGAGCGACTTTAAG

ACCTGGCTCAAGGCCAAGCTCATGCCACAACTGCCCGGGATTCCCTTTATATCCTGCCAA

CGCGGGTATAAGGGGGTCTGGCGGGGAGATGGTATTATGCACACTCGCTGCCATTGTGGA

GCTGAGATTACTGGACATGTCAAAAACGGGACAATGAGGATCGCCGGTCCTAGGACCTGC

AGGAACATGTGGAGTGGAACCTTCCCCATCAACGCCTACACCACGGGCCCCTGCACCCCC

CTT

>MT259617

TCTGGTTCCTGGCTAAGGGACATATGGGACTGGATATGCGAAGTGCTGAGCGACTTTAAG

ACCTGGCTGAAAGCCAAGCTCATGCCACAACTGCCCGGGATCCCCTTCATGTCCTGCCAG

CGCGGGTATAGGGGGGTCTGGCGAGGAGACGGCATTATGCACACTCGCTGCCACTGTGGA

GCTGAGATCACTGGACATGTCAAAAACGGGACGATGAGGATCGTCGGTCCTAGGACCTGT

AAGAACATGTGGAGTGGAACCTTCCCCATTAACGCCTACACCACGGGCCCCTGTACCCCC

CTT

>MT259618

TCCGGTTCCTGGTTAAGGGACATCTGGGACTGGATATGCGAGGTGCTGAGCGATTTTAAG

ACCTGGCTGAAGGCCAAGCTCATGCCACAACTGCCCGGGATCCCCTTTATGTCCTGCCAA

CGTGGGTATAGGGGGGTCTGGCGGGGAGATGGTATTATGCACACTCGCTGCCATTGTGGA

GCTGAGATCACTGGACATGTCAAGAACGGGACGATGAGGATCGTCGGTCCTAAGACCTGC

AGGAACATGTGGAGCGGAACCTTTCCCATTAACGCCTACACCACGGGCCCCTGCACCCCC

CTT

>MT259619

TCCGGCTCCTGGCTAAGGGACATCTGGGACTGGATATGCGAGGTGCTGACCGATTTCAAG

ATCTGGCTGAAGGCCAAGCTTATGCCACAACTGCCCGGGATCCCCTTTGTGTCCTGCCAA

CGCGGGTATAGGGGGGTCTGGCAAGGAGATGGTGTCATGCACACTCGCTGCCATTGTGGA

GCCGAGATCACTGGACATGTCAAAAACGGGACGATGAGGATCGTCGGTCCTAAGACCTGC

AGAAACATGTGGAGTGGGACCTTCCCCATCAACGCCTACACCACGGGCCCCTGCACCCCC

CTT

>MT259620

TCCGGTTCCTGGCTAAGGGACATCTGGGACTGGATATGCGAGGTGTTGAGCGACTTTAAG

ACCTGGCTGAAGGCCAAGCTCATGCCGCAACTGCCCGGGATTCCCCTCGTGTCCTGCCAG

CGCGGGTATAGAGGGGTCTGGCGAGGAGACGGCATCATGCACACTCGCTGCCACTGTGGA

GCTGAAATTACCGGACATGTCAAAAACGGGACGATGAGGATCGCCGGTCCTAGGACCTGC

AGGAACATGTGGAGTGGGACCTTCCCCATTAACGCCTACACCACGGGCCCCTGTGTTCCC

CTC

>MT259621

TCCGGTTCCTGGTTAAGGGACATCTGGGACTGGATATGCGAGGTGCTGAGCGATTTTAAG

ACCTGGCTGAAGGCCAAGCTCATGCCACAACTGCCTGGGATCCCCTTTGTGTCCTGCCAA

CGCGGGTATAGGGGGGTTTGGCGAGGAGATGGTATTATGCACACTCGCTGCCACTGTGGA

GCTGAGATTACTGGACATGTCAAGAACGGGACGATGAGGACCGTCGGTCCCAAGACCTGC

AGGAACATGTGGAGTGGGACCTTCCCCATCAACGCCTACACCACGGGCCCCTGCACCCCC

CTT

>MT259622

TCCGGTTCCTGGTTAAGGGACATCTGGGACTGGATATGCGAGGTGCTGAGCGATTTTAAG

ACCTGGCTGAAGGCCAAGCTCATGCCACAACTGCCCGGGATTCCCTTTGTATCCTGCCAA

CGCGGGTATAGGGGGGTCTGGCGGGGAGATGGCATTATGCACACTCGCTGCCATTGTGGA

GCTGAAATCGCTGGACATGTCAAGAACGGGACGATGAGGATCGTCGGTCCTAAGACCTGC

AGGAACGTGTGGAGTGGGACCTTCCCCATCAACGCCTACACTACGGGCCCCTGCACCCCC

CTT

>MT259623

GCCGGTTCCTGGTTAAGGGACATCTGGGACTGGATATGCGAGGTGCTGAGCGATTTTAAG

ACCTGGCTGAAGGCCAAGCTCATGCCACAACTGCCTGGGATTCCCTTTGTGTCCTGCCAA

CGCGGGTATAGAGGGGTCTGGCGAGGAGATGGTATTATGCACACTCGCTGCCATTGTGGA

GCTGAGATTGCTGGACATGTCAAGAACGGGACGATGAGGATCGTCGGTCCTAAGACCTGC

AGGAACACATGGAGTGGGACCTTCCCCATCAACGCCTACACCACAGGCCCCTGCACCCCC

CTT

>MT259624

TCTGGTTCCTGGCTAAGGGACATATGGGACTGGATATGCGAAGTGCTGAGCGACTTTAAG

ACCTGGCTGAAAGCCAAGCTCATGCCACAACTGCCCGGGATCCCCTTCATGTCCTGCCAG

CGCGGGTATAGGGGGGTCTGGCGAGGAGACGGCATTATGCACACTCGCTGCCACTGTGGA

GCTGAGATCACTGGACATGTCAAAAACGGGACGATGAGGATCGTCGGTCCTAGGACCTGT

AAGAACATGTGGAGTGGAACCTTCCCCATTAACGCCTACACCACGGGCCCCTGTACCCCC

CTT

>MT259625

TCCGGCTCCTGGTTACGGGAGATCTGGGACTGGATATGCGAGGTGCTGAGCGATTTTAAG

ACCTGGCTAAAGGCCAAGCTCATGCCACAACTGCCCGGGATTCCCTTTGTGTCCTGCCAG

CGCGGGTATAGGGGGGTCTGGCGAGGAGATGGCATTATGCACACTCGCTGCCATTGTGGA

GCTGAAATTACTGGACATGTCAAGAACGGGACGATGAGGATCGCCGGTCCTAAGACCTGC

AAGAACATGTGGAGTGGGACCTTCCCCATCAACGCCTACACCACGGGCCCTTGCACCCCC

CTT

>MT259626

TCCGGTTCCTGGTTAAGGGACATCTGGGACTGGATATGCGAGGTGCTGAGCGATTTTAAG

ACCTGGCTGAAGGCCAAGCTCATGCCACAACTGCCTGGGATTCCCTTTGTGTCCTGCCAA

CGCGGGTATAGGGGGGTCTGGCGGGGAGATGGCATCATGCACACTCGCTGCCATTGTGGA

GCCGAGATCACCGGACATGTCAAGAACGGGACGATGAGGATCGTCGGACCCAAGACCTGC

AGGAACATGTGGAGTGGGACCTTCCCCATCAACGCCTACACCACGGGCCCCTGTACCCCC

CTC

>MT259627

TCCGGTTCCTGGTTAAGAGACATCTGGGACTGGATATGCGAGGTGCTGAGCGATTTTAAG

ACCTGGCTGAAGGCCAAGCTCATGCCACAACTGCCCGGGATTCCCTTTGTGTCCTGCCAA

CGCGGGTACAAGGGAGTCTGGCGAGGAGATGGCATTATGCACACCCGCTGCCATTGTGGA

GCTGAAATCACTGGACATGTCAAAAACGGGACGATGAGGATCGTCGGTCCTAAGACCTGC

AAGAACATGTGGAGTGGGACCTTCCCCATCAATGCCTACACCACTGGCCCCTGCACCCCC

CTT

>MT259628

TCCGGTTCCTGGCTAAGGGACATCTGGGACTGGATATGCGAGGTGCTGAGCGACTTTAAG

ACCTGGCTGAAGGCCAAGCTCATGCCACAACTGCCTGGGATTCCCTTTGTATCCTGCCAA

CGCGGGTATAAGGGAGTCTGGCGAGGGGATGGCATCATGCACACTCGCTGCTGCTGTGGA

GCTGAAATCACCGGACATGTCAAGAACGGGACGATGAGGATCGTCGGTCCTAAGACCTGC

AGGAACATGTGGAGTGGGACCTTCCCTATCAACGCCTACACCACGGGCCCCTGTACTCCC

CTT

>MT259629

TCCGGTTCCTGGCTAAGGGACATCTGGGACTGGATATGCGAGGTGCTGAGCGACTTTAAG

ACCTGGCTGAAGGCCAAGCTCATGCCACAACTGCCTGGGATTCCCTTTGTATCCTGCCAA

CGCGGGTATAAGGGAGTCTGGCGAGGGGATGGCATCATGCACACTCGCTGCTGCTGTGGA

GCTGAAATCACCGGACATGTCAAGAACGGGACGATGAGGATCGTCGGTCCTAAGACCTGC

AGGAACATGTGGAGTGGGACCTTCCCTATCAACGCCTACACCACGGGCCCCTGTACTCCC

CTT

>MT259630

TCCGGTTCCTGGTTAAGAGACATCTGGGACTGGATATGCGAGGTGTTGAGCGATTTTAAG

ACCTGGCTAAAGGCCAAGCTCATGCCACAACTGCCCGGGATTCCCTTTGTGTCCTGCCAA

CGCGGGTACAAGGGAGTCTGGCGAGGAGACGGCATTATGCACACCCGCTGCCATTGTGGA

GCTGAGATTACTGGACATGTCAAAAACGGGACGATGAGGATCGTCGGTCCTAAGACCTGC

AAGAACATGTGGAGTGGGACCTTCCCCATCAATGCCTACACCACTGGCCCCTGCACCCCC

CTT

>MT259631

TCCGGTTCCTGGTTAAGGGACATCTGGGACTGGATATGCGAGGTGCTGAGCGACTTTAAG

ACCTGGCTGAAGGCCAAGCTCATGCCACAACTGCCTGGGATTCCCTTTGTGTCCTGCCAA

CGCGGGTATAGGGGGGTCTGGCGAGGAGATGGTATCATGCACACTCGCTGCTATTGTGGA

GCGGAGATCACTGGACATGTCAAGAACGGGACGATGAGGATCGTCGGTCCTAAGACCTGC

AGGAACATGTGGAGTGGGACCTTCCCCATCAACGCCTACACCACGGGCCCCTGTACCCCC

CTT

>MT259632

TCCGGCTCCTGGTTAAGGGACATCTGGGACTGGATATGCGAGGTGCTGAGCGATTTTAAA

ACCTGGCTGAAGGCCAAGCTCATGCCACAGCTGCCCGGGATCCCCTTTGTGTCCTGCCAA

CGCGGGTACAGAGGAGTCTGGCGGGGAGATGGTATTATGCACACTCGCTGCTCTTGTGGA

GCTGAAATCACTGGACATGTCAAGAACGGGACGATGAGGATCGTCGGTCCTAAGACCTGC

AGGAACATGTGGAGTGGGACCTTCCCCATCAACGCCTACACCACGGGCCCCTGCACCCCC

CTT

>MT259633

TCCGGTTCCTGGTTAAGGGACATCTGGGACTGGATATGCGAGGTGCTGAGCGATTTTAAG

ACCTGGCTGAAGGCCAAGCTCATGCCACAACTGCCCGGGATTCCCTTTGTGTCCTGCCAA

CGCGGGTATAAGGGGGTCTGGCGAGGGGATGGTATTATGCACACTCGCTGCCATTGTGGA

GCGGAGATCACTGGACATGTCAAGAATGGGACGATGAGGATCGTCGGTCCTAAGACCTGC

AGGAACATGTGGAGTGGGACCTTCCCCATCAACGCCTACACCACGGGCCCCTGTACCCCC

CTT

>MT259634

TCCGGTTCCTGGTTAAGGGACGTCTGGGACTGGATATGCGAGGTGCTGAGCGATTTTAAG

ACCTGGCTGAAGGCCAAGCTCATGCCACAACTGCCCGGGATTCCCTTTGTGTCCTGCCAA

CGCGGGTATAGGGGGGTCTGGCGAGGAGATGGTGTCATGCACACTCGCTGCCAGTGTGGA

GCTGAAATTACTGGACATGTCAAGAACGGGACGATGAGGATCGTCGGTCCTAAGACCTGC

AGGAACATGTGGAGTGGGACCTTCCCCATCAACGCCTACACCACGGGCCCCTGCACCCCC

CTT

>MT259635

TCCGGTTCCTGGTTAAGGGACATCTGGGACTGGATATGCGAGGTGCTGAGCGATTTTAAG

ACCTGGCTGAAGGCCAAGCTCATGCCACAACTGCCTGGGATCCCCTTTGTGTCCTGCCAA

CGCGGGTATAGGGGGGTTTGGCGAGGGGATGGTATCATGCACACTCGCTGCTGCTGTGGA

GCTGAAATTACTGGACATGTCAAGAACGGGACGATGAGGACCGTCGGTCCCAAGACCTGC

AGGAACATGTGGAGTGGGACCTTCCCTATCAACGCCTACACCACGGGCCCCTGCACCCCC

CTT

>MT259636

TCCGGTTCCTGGCTAAGGGACATCTGGGACTGGATATGCGAGGTGCTGAGCGATTTTAAG

ACCTGGCTAAAAGCCAAGCTCATGCCACAACTGCCTGGGATTCCCTTTGTGTCCTGCCAG

CGCGGGTATAAGGGAGTCTGGCGGGGGGACGGCATCATGCACACTCGCTGCCACTGTGGA

GCTGAGATCACTGGACATGTCAAAAACGGGACGATGAGGATCGTAGGTCCTAGGACCTGC

AGGAACATGTGGAACGGGACCTTCCCCATCAACGCCTACACCACAGGCCCCTGCACTCCC

CTT

>MT259637

TCCGGTTCCTGGTTGAGGGACATCTGGGACTGGATATGCGAGGTGCTGAGCGATTTTAAG

ACCTGGCTGAAGGCCAAGCTCGTGCCACAACTGCCGGGGATTCCCTTTGTGTCCTGCCAA

CGCGGGTATAGGGGGGTCTGGCAAGGAGATGGCATTATGCACACTCGCTGCCATTGTGGA

GCTGAAATCACTGGACATGTCAAGAACGGGACGATGAGGATCGTCGGTCCTAAGACCTGC

AGGAACATGTGGAGTGGGACCTTCCCCATCAACGCCTACACCACGGGCCCCTGTACCCCC

CTT

>MT259638

TCCGGTTCCTGGTTAAGAGACATCTGGGACTGGATATGCGAGGTGCTGAGCGATTTTAAG

ACCTGGCTGAAGGCCAAGCTCATGCCACAACTGCCTGGGATTCCCTTTGTGTCCTGCCAA

CGCGGGTATAGGGGGGTCTGGCGAGGAGATGGTATTATGCACACTCGCTGCCATTGTGGA

GCTGAAATCACTGGACATGTCAAGAACGGGACGATGAGGATCGTCGGTCCTAAGACCTGC

AAGAACATGTGGAGCGGGACCTTCCCCATCAACGCCTACACCACGGGCCCCTGTACCCCC

CTT

>MT259639

TCCGGCTCCTGGTTAAGGGACATCTGGGACTGGATATGCGAGGTGCTGAGCGATTTTAAG

ACCTGGCTAAAGGCCAAGCTCATGCCACAACTGCCCGGGATTCCCTTTGTATCCTGCCAA

CGCGGGTATAAGGGGGTCTGGCGGGGAGATGGTATTATGCACACTCGCTGCCATTGTGGA

GCTGAGATCACTGGACATGTCAAAAACGGGACGATGAGGATCGTCGGTCCTAGGACCTGC

AGGAACATGTGGAG--GAACCTTCCCCATCAACGCCTACACCACGGGCCCCTGCACCCCC

CTT

>MT259640

TCCGGTTCCTGGCTACGGGACATCTGGGACTGGATATGCGAGGTGCTGAGCGACTTTAAG

ACCTGGCTGAAAGCCAAGCTCATGCCGCAACTGCCCGGGATCCCCTTTGTGTCCTGCCAG

CGCGGGTATAGGGGGGTCTGGCGAGGAGACGGCATCATGCACACTCGCTGCTGCTGTGGA

GCTGAAATTACTGGACATGTCAAAAACGGGACGATGAGGATCGTCGGTCCTAAGACCTGC

AAGAACATGTGGAGTGGGACCTTCCCCATTAACGCCTACACCACGGGCCCCTGTACTCCC

CTT

>MT259641

TCCGGTTCCTGGTTAAGGGACATCTGGGACTGGATATGCGAGGTGCTGAGCGATTTTAAG

ACCTGGCTGAAAGCTAAGCTCATGCCACAACTGCCAGGGATTCCCTTTATGTCCTGCCAA

CGCGGGTATAGGGGGGTCTGGCGAGGAGATGGTGTTATGCACACTCGCTGCCCTTGTGGA

GCTGAAATCACTGGACATGTCAAGAACGGGACGATGAGGATCGTCGGTCCTAAAACCTGC

AGGAACATGTGGAGTGGGACCTTCCCCATCAACGCCTACACCACGGGCCCCTGCACCCCC

CTT

>MT259642

TCCGGTTCTTGGTTGAGGGACATCTGGGACTGGATATGCGAAGTGCTGAGCGATTTTAAG

ACCTGGCTGAAGGCCAAGCTCATGCCACAACTGCCTGGGATTCCCTTTGTGTCCTGCCAA

CGCGGGTATAGGGGGGTCTGGCGAGGAGACGGCATTATGCACACTCGCTGCCACTGTGGA

GCTGAGATTACTGGACATGTCAAGAACGGGACGATGAGGATCGTCGGTCCTAAGGCCTGC

AGGAACACATGGAGTGGGACCTTCCCCATCAACGCCTACACCACGGGCCCCTGCACCCCC

CTT

>MT259643

TCCGGTTCCTGGTTAAGAGACATCTGGGATTGGATATGCGAGGTGCTGAGCGACTTTAAG

ACCTGGCTGAAGGCCAAGCTCATGCCACAACTGCCCGGGATTCCCTTTGTGTCCTGCCAG

CGCGGGTACAGGGGGGTCTGGCGGGGAGATGGTATTATGCACACTCGCTGCCATTGTGGA

GCTGAAATCACTGGACATGTCAAGAACGGGACGATGAGGATCGTTGGCTCCAAGACCTGC

AGGAACATGTGGAATGGAACCTTCCCCATCAACGCCTATACCACGGGCCCCTGCTCCCCC

CTT

>MT259644

TCGGGTTCCTGGCTAAGGGACATCTGGGACTGGATATGCGAGGTGCTGAGCGACTTTAAG

ACCTGGCTGAAAGCCAAGCTCGTGCCACAACTGCCCGGGATTCCCCTCATGTCCTGCCAG

CGCGGGTATAGGGGGGTCTGGCGAGGAGATGGCATTATGCACACTCGCTGCCATTGTGGA

GCTGAGATCACTGGACATGTCAAAAACGGGACGATGAGGATCGTCGGTCCCAGGACCTGC

AGGAATATGTGGAGTGGGAGTTTCCCCATTAACGCCTACACCACGGGCCCCTGTTCTCCT

CTT

>MT259645

GCCGGTTCCTGGTTAAGGGACATCTGGGACTGGATATGCGAGGTGCTGAGCGATTTTAAG

ACCTGGCTGAAGGCCAAGCTCATGCCACAACTGCCTGGGATTCCCTTTGTGTCCTGCCAA

CGCGGGTATAGAGGGGTCTGGCGAGGAGATGGTATTATGCACACTCGCTGCCATTGTGGA

GCTGAGATTGCTGGACATGTCAAGAACGGGACGATGAGGATCATCGGTCCTAAGACCTGC

AGGAACACATGGAGTGGGACCTTCCCCATCAACGCCTACACCACAGGCCCCTGCACCCCC

CTT

>MT259646

TCCGGTTCCTGGCTAAGGGACATCTGGGACTGGATATGCGAGGTGCTGAGCGATTTCAAG

ACCTGGCTGAAGGCCAAGCTCATGCCACAACTGCCCGGGATCCCCTTTGTGTCCTGCCAA

CGCGGGTATAGAGGGGTCTGGCGAGGGGATGGCATCATGCACACTCGCTGCCACTGTGGG

GCCGAAATCACCGGACATGTCAAAAACGGGACGATGAGGATCGTCGGTCCCAGGACCTGC

AGGAACATGTGGAACGGGACCTTCCCCATCAATGCCTACACCACGGGCCCCTGCACCCCT

CTT

>MT259647

TCCGGTTCCTGGCTAAGGGACATCTGGGACTGGATATGCGAGGTGCTGAGCGACTTTAAG

ACCTGGCTAAAAGCTAAGCTCATGCCACAACTGCCTGGGATTCCCTTTGTATCCTGCCAG

CGCGGGTATAGGGGAGTCTGGGGGGGGGACGGCATTATGCACACTCGCTGCCACTGTGGA

GCTGAAATCACTGGACATGTCAAAAACGGGACGATGAGGATCGTAGGTCCTAGGACCTGC

AGGAACATGTGGAGTGGGACCTTCCCCATCAACGCCTACACCACAGGCCCCTGCACTCCC

CTT

>MT259648

TCCGGCTCCTGGTTAAGAGACATCTGGGACTGGATATGTGAGGTGCTGAGCGATTTTAAG

ACCTGGCTGAAGGCCAAGCTTGTGCCACGACTGCCCGGGATCCCCTTTGTGTCCTGCCAG

CGCGGGTATAGGGGGGTCTGGGGCGGAGATGGCATTATGCACACCCGCTGCCATTGCGGA

GCTGAGATCACCGGACATGTCAAGAACGGGACGATGAGGATCGTCGGCCCCAAGACCTGC

AAAAACATGTGGAGTGGGACCTTCCCCATCAACGCCTACACCACGGGCCCCTGTAGCCCC

CTT

>MT259649

TCCGGTTCCTGGTTGAGGGACATCTGGGACTGGATATGCGAGGTGCTGAGCGATTTTAAG

ACCTGGCTAAAGGCCAAGCTCATGCCACAGCTGCCTGGGATCCCCTTCATGTCCTGCCAA

CGCGGGTATAGGGGGGTCTGGCGGGGAGATGGCATTATGCACACTCGCTGCCCTTGTGGA

GCTGAGATCACTGGACATGTCAAGAACGGGACGATGAGGATCGCTGGTCCTAAGACCTGC

AGGAACATGTGGAGTGGGACCTTCCCCATCAACGCCTATACCACGGGCCCCTGCACCCCC

CTT

>MT259650

TCCGGTTCCTGGTTAAGGGACATCTGGGACTGGATATGCGAGGTGCTGAGCGATTTTAAG

ACCTGGCTGAAGGCCAAGCTCATGCCACAACTGCCTGGGATTCCCTTTGTGTCCTGCCAA

CGCGGGTATAAGGGGGTCTGGCGAGGAGATGGTATTATGCACACTCGCTGCCATTGTGGA

GCTGAAATCACTGGACATGTCAAGAACGGGACGATGAGGATCGTCGGTCCTAAGACCTGC

AAGAACATGTGGAGTGGGACCTTCCCCATCAACGCCTACACCACGGGCCCCTGCACCCCC

CTT

>MT259651

TCCGGTTCCTGGTTAAGGGACATCTGGGACTGGATATGCGAGGTGCTGAGCGATTTTAAG

ACCTGGCTGAAGGCCAAGCTCATGCCACAACTGCCTGGGATTCCCTTTGTGTCCTGCCAA

CGCGGGTATAGGGGGGTCTGGCGGGGGGATGGCATTATGCACACTCGCTGCCATTGTGGA

GCTGAGATTGCTGGACATGTCAAGAACGGGACGATGAGGATCGTCGGTCCTAAGACCTGC

AGGAACATGTGGAGTGGGACCTTCCCCATCAACGCCTACACCACAGGCCCCTGCACCCCC

CTT

>MT259652

TCCGGTTCCTGGTTGAGGGACGTCTGGGACTGGATATGCGAGGTGCTGAGCGATTTTAAG

ACCTGGCTGAAGGCCAAGCTCATGCCACAACTGCCCGGGATTCCCTTTGTGTCCTGCCAG

CGCGGGTATAAGGGGGCCTGGAAAGGAGATGGTATTATGCACACTCGCTGCCATTGTGGA

GCTGAAATCACCGGACATGTCAAGAACGGGACGATGAGGATCGTCGGTCCCAGGACCTGC

AGGAACATGTGGAGTGGGACCTTCCCCATCAACGCCTACACCACGGGCCCTTGCACCCCC

CTT

>MT259653

TCCGGTTCCTGGTTAAGGGACATCTGGGACTGGATATGCGAGGTGCTGAGCGATTTTAAG

ACCTGGTTGAAGGCCAAGCTCATGCCACAACTGCCTGGGATCCCCTTTGTGTCCTGCCAA

CGCGGGTATAAGGGGGTCTGGCGGGGAGATGGCGTTATGCACACCCGCTGCCATTGCGGA

GCTGAAATCACTGGACATGTCAAGAACGGGACGATGAGGATCGTCGGTCCTAAGACCTGC

AAGAACATGTGGAGTGGGACCTTCCCCATCAACGCCTACACCACGGGTCCCTGTACCCCC

CTT

>MT259654

TCCGGCTCCTGGTTAAGAGACATCTGGGACTGGATATGCGAGGTGCTGAGCGATTTCAAG

ACCTGGCTGAAGGCCAAGCTCATGCCACAACTACCTGGGATCCCCTTTGTGTCCTGCCAA

CGCGGGTATAAGGGGGTCTGGCGGGGAGATGGTATTATGCACACTCGCTGCCACTGTGGT

GCTGAGATCACCGGACATGTCAAGAACGGGACGATGAGGATTGTCGGCCCTAAGACCTGC

AGGAACACGTGGAGTGGGACCTTCCCCATCAACGCCTACACCACAGGCCCCTGCAACCCC

CTT

>MT259655

TCCGGTTCCTGGTTAAGGGACATCTGGGACTGGATATGCGAGGTGCTGAGCGATTTTAAG

ACCTGGCTGAAGGCCAAGCTCATGCCACAACTGCCCGGGATTCCCTTTGTGTCCTGCCAA

CGCGGGTATAGGGGAGTCTGGCGAGGAGATGGTATTATGCACACTCGCTGCCATTGTGGA

GCTGAAATCACTGGACATGTCAAGAACGGGACGATGAGGATCGTCGGTCCCAAGACCTGC

AAGAACATGTGGAGTGGGACTTTCCCCATCAACGCCTACACCACGGGCCCCTGCACTCCC

CTT

>MT259656

TCCGGTTCCTGGTTAAGGGACATCTGGGACTGGATATGCGAGGTGCTGAGCGATTTTAAG

ACCTGGCTGAAGGCCAAGCTCATGCCACAACTGCCTGGGATTCCCTTTGTGTCCTGCCAG

CGCGGGTATAGAGGGGTCTGGCGAGGAGATGGTATTATGCACACTCGCTGCCATTGTGGA

GCTGAGATTGCTGGACATGTCAAGAACGGGACGATGAGGATCGTCGGTCCTAAGACCTGC

AGGAACACATGGAGTGGGACCTTCCCCATCAACGCCTACACCACAGGGCCCTGCACCCCC

CTT

>MT259657

TCCGGCTCATGGCTAAGGGACATCTGGGACTGGATATGCGAGGTGCTGAGCGACTTTAAG

ACCTGGCTCAAGGCCAGGCTCATGCCACAACTGCCCGGGATTCCCTTTATATCCTGCCAA

CGCGGGTATAGGGGGGTCTGGCGGGGAGATGGTATTATGCACACTCGCTGCCATTGTGGA

GCTGAGATTACTGGACATGTCAAAAACGGGACAATGAGGATCGCCGGTCCTAGGACCTGC

AGGAACATGTGGAGTGGAACCTTCCCCATCAACGCCTACACCACGGGCCCCTGCACCCCC

CTT

>MT259658

TCTGGTTCCTGGTTAAGGGACATCTGGGACTGGATATGCGAGGTGCTGAGCGATTTTAAG

ACCTGGCTGAAGGCTAAGCTCACACCACACCTGCCCGGGATTCCCTTCGTGTCCTGCCAA

CGCGGGTATAGGGGGGTCTGGCGGGGAGATGGTATTATGCACACTCGCTGCCATTGCGGA

GCTGAGATCACTGGACATGTCAAGAACGGGACGATGAGGATCGTCGGTCCTAGGACCTGT

AAGAACATGTGGAGTGGAACCTTCCCCATCAACGCCTACACCACGGGCCCCTGCACCCCC

CTT

>MT259659

TCCGGTTCCTGGCTAAGGGACGTCTGGGACTGGATATGCGAGGTGCTGAGCGACTTTAAG

ACCTGGCTAAAAGCCAAGCTCATGCCACAACTGCCTGGGGTTCCCTTTGTGTCCTGCCAA

CGCGGGTATAAGGGAGTCTGGCGGGGGGACGGCATCATGCACACTCGCTGCCACTGTGGA

GCTGAGATCACTGGACATGTCAAAAACGGGACGATGAGGATCGTAGGTCCTAGGACCTGC

AGAAACATGTGGAGTGGGACCTTCCCCATCAACGCCTACACCACAGGCCCCTGTACTCCC

CTT

>MT454922

CACATTTGACAGACTGCAGGTTCTGGACAGCCACTACCAGGACGTGCTTAAGGAGGTTAA

GGCGGCGGCGTCAAAAGTGAAGGCTAACTTGCTATCTGTAAAGGAAGCTTGCAGCCTGAC

GCCCCCACATTCAGCCAGATCCAAATTTGGCTACGGGGCAAAGGACGTCCGTTGCCATGC

CAGAAAGGCCGTAAACCACATCAACTCCGTGTGGGAGGACCTTCTGGAAGACAGTGTAAC

ACCAATAGACACTACCATCATGGCTAAGAACGAAGTTTTCTGCGTCCAGCCTGAGAAGGG

AGGTCGTAAGCCAGCTCGACTCATCGTGTACCCCGACCTGAGTGTGCGCGTGTGCGAGAA

AATGGCCCTGTACGACGTGGTCAGCAAACTCCCTATAGCCGTGATGGGAAGCTCCTACGG

ATTCCAATACTCACCAGGGCAGCGGGTTGAATTCCTCGTGCAAGCGTGGAAGTCCAAAAA

GACCCCAATGGGGTTTTCATATGATACCCGCTGTTTTGACTCTACAGTCACTGAAAGCGA

CATCCGTACGGAGGAAGCAATCTACCAGTGTTGTGACCTGGACCCCCAAGCCCGCGTGGC

CATCAAGTCCCTCACCGATAGGCTTTATGTCGTGGGACCT

>MT454923

CACATTTGACAGACTGCAGGTCCTGGATAACCATTACCAGGACGTGCTTAAGGAGGTTAA

GGCCGCGGCGTCAAAAGTGAAGGCTAACTTGCTATCCGTAGAGGAAGCTTGCAGCCTGAC

GCCCCCACACTCAGCCAAATCCAAATTTGGCTACGGGGCGAAAGACGTCCGTTGCCATGC

CAGAAAGGCCGTGGACCACATCAACTCCGTGTGGAAGGACCTTCTGGAAGACAGTGTAAC

ACCAATAGACACTACCATCATGGCCAAGAATGAAGTTTTCTGTGTCCAACCTGAGAAGGG

GGGTCGTAAGCCAGCTCGACTCATCGTGTTCCCCGATCTGGGTGTGCGCGTGTGCGAGAA

GATGGCCCTGTACGACGTAGTCAGCAAACTCCCTGTAGCCGTGATGGGAAGCTCCTACGG

ATTTCAGTACTCACCAGGGCAGCGGGTTGAATTCCTCGTGCAAGCGTGGAAGTCCAAGAA

GATCCCAATGGGGTTCTCGTATGATACCCGCTGCTTTGACTCTACAGTCACTGAGAGCGA

CATCCGTACGGAGGAGGCAATTTACCAGTGTTGTGACCTGGACCCCCAAGCCCGCGTGGC

CATTAAGTCCCTCACCGAGAGGCTTTATGTTGGGGGCCCTCTTACCAATTCAA

>MT454924

CACATTTGACAGGCTGCAGGTTCTGGACAACCATTACCTGGATGTCGTCAAGGAGGTTAA

GGCGGCGGCGTCGAAAGTGAAGGCTAACTTGCTATCCGTGGAGGAAGCTTGTAACCTGAC

GCCCCCACATTCAGCCAAATCCAAATTTGGCTACGGGGCAAAAGACGTCCGTTGCCATGC

CAGAAAGGCCGTAAACCACATCAACTCCGTGTGGAAAGACCTTCTGGAAGACAGTGTAAC

TCCAATAGATACTACCATCATGGCTAAGAACGAAGTTTTCTGCGTTCAGCCTGAGAAGGG

GGGTCGTAAGCCAGCTCGACTCATCGTGTTCCCTGATTTGGGTGTGCGAGTGTGCGAGAA

GATGGCTCTGTACGACGTAGTCAGCAAGCTCCCTATAGCCGTGATGGGAAGCTCCTACGG

ATTCCAATACTCACCAGGACAGCGGGTTGAATTCCTCGTGCAAGCGTGGAAGTCCAAGAA

GACCCCAATGGGGTTTTCGTATGATACCCGCTGCTTTGACTCTACAGTCACTGAGAGCGA

TATCCGTATGGAGGAGGCAATCTACCAGTGTTGTGACCTGGACCCCCAAGCCCGTGTGGC

CATCAAGTCTCTCACCGAGAGGCTTTATGTCGGGGGCCCTCTTACCAATTCAA

>MT454925

CACATTTGACAGAGTGCAGGTTCTGGACAGCCATTACCAGGACGTGCTCAAGGAGGTTAA

GGCGGCGGCGTCGAAAGTGAAGGCCAACTTGCTATCCGTAGAGGAAGCTTGCAGCCTGAC

GCCCCCACATTCAGCCAAATCCAAATTTGGCTACGGGGCAAAGGACGTCCGTTGCCATGC

CAGAAAGGCCGTAAACCACATCAACTCCGTGTGGGAAGACCTTCTGGAAGACAGTGTAAC

ACCAATAGATACCACCATCATGGCTAAGAACGAGGTTTTCTGCGTTCAGCCTGAGAAGGG

AGGTCGTAAGCCAGCTCGTCTCATTGTGTTCCCCGACCTGGGTGTGCGCGTGTGCGAGAA

GATGGCCCTGTACGACGTGGTCAGCAAGCTCCCTCTGGCCGTGATGGGAAGCTCCTACGG

ATTCCAATACTCACCAGGACAGCGGGTTGAATTCCTCGTGCAAGCGTGGAAGTCCAAGAA

GACCCCAATGGGGTTTTCATATGATACCCGCTGCTTTGACTCTACGGTCACTGAGAACGA

CATCCGTACGGAGGAGGCGATCTACCAGTGTTGTGACCTTGACCCCCAAGCCCGCGTGGC

CATCAAGTCCCTCACCGAGAGGCTTTATGTCGGGGGTCCTCTTACCAATTCAC

>MT454926

CACATTTGACAGACTGCAGGTTCTGGACAACCATTACCAGGACGTGCTCAAGGAGGTTAA

GGCGGCGGCGTCAAAAGTGAAGGCTAACTTGCTGTCCGTAGAGGAAGCTTGTAGCCTGAC

GCCCCCACACTCAGCCAGATCCAAATTTGGCTACGGGGCAAAAGACGTCCGGTGCCATGC

CAGAAAGGCCGTAAACCACATCAACTCCGTGTGGAAAGACCTTCTGGAAGACAGTGTAAC

ACCAATAGATACTACCATCATGGCTAAGAACGAAGTTTTCTGCGTTCAGCCTGAGAAGGG

GGGTCGTAAGCCAGCTCGTCTCATCGTGTTCCCCGATCTGGGTGTGCGCGTGTGCGAGAA

GATGGCTTTGTACGACGTAGTCAGCAAACTCCCTGTAGCCGTGATGGGAAAGTCCTACGG

ATTCCAATACTCGCCAGGGCAGCGGGTTGAATTCCTCGTGCAAGCGTGGAAGTCCAAGAA

GACCCCAATGGGGTTTTCGTATGATACCCGCTGCTTTGACTCTACAGTCACCGAGAGCGA

TATCCGTACGGAGGAGGCAATCTACCAGTGTTGTGACCTGGACCCCCAAGCCCGCGTGGC

CATCAAGTCCCTCACCGAGAGGCTTTATGTTGGGGGCCCTCTTACCAATTCCA

>MT454927

CACATTTGACAGACTGCAGGTTCTGGACAACCACTACCAGGACGTGCTCAAGGAGGTTAA

GGCGGCGGCGTCAAAAGTGAAGGCTAACCTGCTATCCGTAGAGGAAGCTTGCAGCCTGAC

GCCCCCACATTCAGCCAAATCTAAATTTGGCTATGGGGCGAAAGACGTCCGTTGCCATGC

CAGAAAGGCCGTGAACCACATCAACTCCGTGTGGAAGGACCTTCTGGAAGACAGTGTAAC

ACCAATAGATACTACCATCATGGCTAAGAACGAAGTCTTCTGCGTTCAGCCTGAGAAGGG

AGGTCGTAAGCCGGCTCGACTCATCGTGTTCCCCGACCTGGGTGTACGCGTGTGCGAGAA

GATGGCTCTGTACGACGTAGTTAGCAAACTCCCTGTAGCCGTGATGGGAAGCTCCTACGG

ATTCCAATACTCACCAGGGCAGCGGGTTGAATTCCTCGTGCAAGCGTGGAAGTCCAAGAA

GACCCCAATGGGGTTTTCGTATGATACCCGCTGCTTTGACTCTACAGTCACTGAGAGCGA

TATCCGTACGGAGGAGTCAATTTACCAAAGTTGTGACCTGGACCCCCAAGCCCGTGTGGC

TATCAAGTCCCTCACTGAGAGGCTTTATGTTGGGGGTCCTCTTACCAATTCAA

>MT454928

CACATTTGACAGACTGCAAGTTCTGGACACCCATTACCAGGACGTGCTCAAGGAGGTTAA

GGCAGCGGCGTCAAAAGTGAAGGCTAACTTGCTATCCGTGGAGGAAGCTTGCAGCCTGAC

GCCCCCACACTCGGCCAAATCCAAGTTTGGCTATGGGGCAAAGGACGTCCGTTGCCATGC

CAGGAAGGCCGTAAACCACATCAGCTCCGTGTGGAAAGACCTTCTGGAAGACAGTGTAAC

ACCAATAGACACTACCATCATGGCCAAGAACGAGGTTTTCTGCGTTGAGCCTGAGAAGGG

GGGTCGTAAGCCAGCTCGCCTCATCGTGTTCCCCGACTTGGGCGTGCGCGTGTGCGAGAA

GATGGCACTATACGACGTGGTTAGCAAGCTCCCCCTGGCCGTGATGGGAAGCTCCTACGG

ATTCCAATACTCACCAGGACAGCGGGTTGAATTCCTCGTGCAAGCGTGGAAGTCCAAGAA

GACCCCAATGGGGTTCTCATATGATACCCGCTGCTTTGACTCCACAGTCACTGAGAGCGA

TATCCGTACGGAGGAAGCAATCTACCAATGTTGTGACCTGGACCCCCAAGCCCGCGTGGC

CATCAGGTCTCTCACTGAGAGGCTTTATGTTGGGGGCCCTCTTACCAATTCAA

>MT454929

CACATTTGACAGACTGCAGGTTCTGGACAGCCATTACCCGGACGTGCTCAAGGAGGTTAA

GGCAGCGGCGTCAAAAGTGAAGGCTAACTTGCTATCCGTAGAGGAAGCTTGCAGCCTGAC

GCCCCCACATTCAGCCAGATCCAAATTTGGCTATGGGGCAAAAGACGTCCGTTGCCATGC

CAGAAAGGCCGTGAACCACATCAACTCCGTGTGGAAGGACCTTCTGGAGGACAATGTAAC

ACCAATAGATACCACCATCATGGCTAAGAACGAAGTTTTCTGCGTTCAGCCTGAGAAGGG

GGGTCGTAAGCCAGCTCGCCTCATCGTGTTCCCCGATCTGGGTGTACGCGTGTGCGAGAA

GATGGCCCTGTACGACGTGATCAACAAACTCCCTACAGCCGTGATGGGAAGCTCCTACGG

ATTCCAATACTCGCCAGGGCAGCGGGTTGAATTCCTCGTGCAAGCGTGGAAGTCCAAGAA

GACCCCGATGGGGTTTTCGTATGATACCCGCTGCTTTGACTCCACAGTCACCGAGAACGA

TATCCGTACGGAGGAGGCAATCTACCAGTGTTGTGACCTGGACCCCCAAGCCCGTGTGGC

CATCAAGTCCCTCACCGAGAGGCTTTATGTCGGGGGCCCTCTTACCAATTCAA

>MT454930

CACATTTGACAGACTGCAAGTTCTGGACAGCCACTACCAGGACGTGCTCAAGGAGGTTAA

GGCGGCGGCGTCAAAAGTGAAGGCCAACTTGCTATCCGTAGAGGAAGCTTGCAGCCTGAC

GCCCCCACATTCAGCCAAATCCAAATTTGGCTACGGGGCAAAAGACGTCCGTTGCCATGC

CAGAAAGGCCGTAAACCACATCAACTCCGTGTGGAAAGACCTTCTGGAAGACAGTGTAAC

ACCAATAAGTACTACCATCATGGCTAAGAACGAAGTTTTCTGCGTTCAGCCTGAGAAGGG

GGGTCGTAAGCCAGCTCGACTCATCGTGTTCCCCGACCTGGGTGTGCGCGTGTGCGAGAA

GATGGCCTTGTACGACGTGATCAGCAAACTCCCTACAGCCGTGATGGGAAGCTCCTACGG

ATTCCAATACTCACCAGGGCAGCGGGTTGAATTCCTCGTGCAAGCGTGGAAGTCCAAGAA

GACCCCAATGGGGTTTTCGTATGATACCCGCTGCTTCGACTCTACAGTCACCGAGAGCGA

TATCCGTACGGAGGAGGCAATCTACCAGAGTTGTGACCTGGACCCCCAAGCCCGCGTGGC

CATCAAGTCCCTCACCGAGAGGCTTTATGTTGGGGGCCCTCTTATCAATTCAA

>MT454931

CACATTTGACAGACTGCAGGTTCTGGACAACCACTACCAGGACGTGCTCAAGGAGGTTAA

GGCGGCGGCGTCAAAAGTGAAGGCTAACTTGCTATCCGTAGAGGAAGCTTGCAGCCTGAC

GCCCCCACATTCAGCCAAGTCCAAATTTGGCTACGGGGCAAAAGACGTCCGTTGCCATGC

CAGAAAGGCCGTAAACCACATCAACTCCGTGTGGAAGGACCTTCTGGAAGACAGTGTAAC

ACCAATAGATACTACCATCATGGCTAAGAACGAAGTTTTTTGCGTTCAGCCTGAGAAGGG

AGGTCGTAAGCCAGCTCGACTCATCGTGTTCCCCGATCTGGGTGTGCGCGTGTGCGAGAA

GATGGCCCTGTACGACCTAGTCAGCAAACTCCCTATAGCCGTGATGGGAAGCTCCTACGG

ATTCCAGTACTCACCAGGGCAGCGGGTTGAATTCCTCGTGCAAGCGTGGAAGTCCAAGAA

GACCCCAATGGGGTTTTCGTATGATACCCGCTGCTTTGACTCTACAGTCACTGAGAGCGA

TATCCGTACGGAGGAGGCAATTTATCAGTGTTGTGACCTGGACCCCCAAGCCCGCGTGGC

CATCAAGTCCCTCACCGAGAGGCTTTATGTCGGGGGCCCTCTTACCAATTCAA

>MT454932

CACATTTGACAGACTGCAAGTTCTGGATAGCCATTACCAGGACGTGCTCAAGGAGGTTAA

GGCAGCGGCGTCAAAAGTGAAGGCTAACTTGCTATCCGTAGAGGAAGCTTGCAGCCTGAC

GCCCCCACATTCAGCCAAATCCAAGTTTGGCTACGGGGCAAAAGACGTCCGTTGCCATGC

CAGAAAGGCCGTTACCCACATCAACTCCGTGTGGAAAGACCTTCTGGAAGACAGTGTAAC

ACCAATAGACACTACCATCATGGCCAAGAACGAGGTTTTCTGCGTCCAGCCTGAGAAGGG

GGGTCGTAAGCCAGCTCGTCTCATCGTGTTCCCTGACCTGGGAGTGCGCGTGTGCGAGAA

GATGGCCTTGTACGACGTGGTTCGCAAACTTCCCCTGGCCGTGATGGGAAGCTCCTACGG

ATTCCAATACTCACCAGGGCAGCGGGTTGAATTCCTCGTGCAAGCGTGGAAGTCCAAGAA

GTCCCCAATGGGGTTCTCGTATGATACCCGCTGTTTTGACTCCACAGTCACTGAGAGCGA

CATCCGTACGGAGGAGGCAATCTACCAGTGTTGTGACCTGGACCCCCAAGCCCGCGTGGC

CATCAAGTCCCTCACTGAGAGGCTTTATGTTGGGGGCCCTCTTACTAATTCAA

>MT454933

CACATTTGACAGACTGCAGGTTCTGGACAACCACTACCAGGACGTGCTCAAGGAGGTTAA

GGCGGCGGCGTCAAAAGTGAAGGCTAACTTGCTATCCGTGGAGGAAGCTTGTGACCTGAC

GCCCCCACACTCAGCCAAATCCAAATTTGGCTATGGGGCAAAAGACGTCCGTTGCCATGC

CAGAAAGGCCGTAAACCACATCAACTCCGTGTGGAAAGACCTTCTGGAAGACAGTGTAAC

ACCAATAGACACTACCATCATGGCTAAGAACGAAGTTTTCTGCGTTCAGCCTGAGAAGGG

GGGTCGTAAGCCAGCTCGACTCATCGTGTTCCCCGACCTGGGTGTGCGCGTGTGCGAGAA

GATGGCTCTATACGACGTAGTCAGCAAACTCCCTATAGCCGTGATGGGAAGCTCCTACGG

ATTCCAATACTCACCAGGGCAGCGGGTTGAATTCCTCGTGCAAGCGTGGAAGTCCAAGAA

GACCCCAATGGGGTTTTCGTATGATACCCGCTGCTTTGACTCAACAGTCACTGAGAGCGA

TATCCGTACGGAGGAGGCAATCTACCAGTGTTGTGACCTGGACCCCCAAGCCCGCGTGGC

CATCAAGTCCCTCACCGAGAGGCTTTATGTCGGGGGCCCTCTTACCAATTCAA

>MT454934

CACATTTGACAGACAGCAGGTTCTGGACAGCCATTACCAGGACGTGCTCAAGGAGGTTAA

GGCGGCGGCGTCAAAAGTGAAGGCTAACTTGCTATCCGTGGAGGAAGCTTGCAGCCTGAC

GCCCCCACATTCAGCCAGATCCAAATTTGGCTATGGGGCAAAAGACGTCCGTTGCCATGC

CAGAAAGGCCATAAGCCACATCAACTCCGTGTGGAAAGACCTTCTGGAAGACAGTGTAAC

ACCAATAGACACTACCATCATGGCTAAGAACGAAGTTTTCTGCATTCAGCCTGAGAAGGG

AGGTCGTAAGCCAGCTCGACTCATCGTGTTCCCCGACTTGGGTGTGCGCGTGTGCGAGAA

GATGGCCCTGTACGACGTAGTCAGCAAACTCCCTATAGCCGTGATGGGAAGCTCCTACGG

ATTTCAATACTCACCTGGACAGCGGGTTGAATTCCTCGTGCAAGCGTGGAAGTCCAAGAA

GACCCCGATGGGGTTTTCGTATGATACCCGCTGCTTTGACTCTACGGTCACTGAGAGTGA

TATCCGCACGGAGGAGGCAATCTACCAGTGTTGTGACCTGGACCCCCAAGCCCGCGTGGC

CATCAAGTCCCTCACCGAGAGGCTTTATGTCGGGGGCCCTCTTACCAATTCAA

>MT454935

CACATTTGACAGACTGCAGGTTCTGGACAACCACTACCAGGACGTGCTCAAGGAGGTTAA

GGCGGCGGCGTCAAAAGTGAAGGCTAACTTGCTATCTGTAGAGGAAGCTTGCAGCCTGAC

GCCCCCACATTCAGCCAAATCCAAATTTGGCTACGGGGCAAAAGACGTCCGTTGCCATGC

CAGAAAGGCCATAAACCACATCAACTCCGTGTGGAAGGACCTTCTGGAAGACAGTGAAAC

ACCAATAATGACTACCATCATGGCTAAGAACGAAGTTTTTTGCGTTGAGCCTGAGAAGGG

GGGTCGCAAGCCAGCTCGACTCATCGTGTTCCCCGACCTGGGTGTACGCGTGTGCGAGAA

GATGGCCCTGTACGACGTGGTCAGCAAACTCCCCATAGCCGTGATGGGAAGCTCCTACGG

ATTCCAATACTCACCAGGGCAGCGGGTTGAATTCCTCGTGCAAGCGTGGAAGTCCAAGAA

GACCCCAATGGGGTTTTCGTATGATACCCGCTGCTTTGACTCTACAGTCACTGAGAGCGA

TATCCGTACGGAGGAGGCAATCTACCAGTGTTGTGACCTGGACCCCCAAGCCCGCGTGGT

CATCAAGTCCCTCAGCGAGAGGCTTTATGTCGGGGGCCCTCTTACCAATTCAA

>MT454936

CACATTTGACAGACTGCAGGTTCTGGACAACCATTACCAGGACGTGCTCAAGGAGGTTAA

GGCGGCGGCGTCAAAAGTGAAGGCTAACTTGCTGTCCGTGGAGGAAGCTTGCAGCCTGAC

GCCCCCCCATTCAGCCAAATCCAAATTTGGCTACGGGGCAAAAGACGTCCGTTGCCATGC

CAGAAAGGCCGTAAGCCACATCAGCTCCGTGTGGAAAGACCTTCTGGAAGACAGTGTAAC

ACCAATAGATACTACCATCATGGCCAAAAACGAAGTTTTCTGCGTTCAGCCTGAGAAGGG

AGGTCGTAAGCCGGCTCGACTCATCGTGTTCCCCGACCTGGGTGTGCGCGTGTGCGAGAA

GATGGCCCTGTACGACGTAGTCAGCAAACTCCCTATAGCCGTGATGGGAAGCTCCTACGG

ATTTCAATACTCACCAGGGCAGCGGGTTGAATTCCTTGTGCAAGCATGGAAGTCCAAGAA

GACCCCAATGGGGTTTTCGTATGATACCCGCTGCTTTGACTCTACAGTCACTGAGAGCGA

CATCCGTACGGAGGAGGCAATCTACCAGTGTTGTGACCTGGACCCCCAAGCCCGCGTGGC

CATCAAGTCCCTCACCGAGAGGCTTTATGTCGGGGGCCCTCTTACCAATTCAA

>MT454937

CACATTTGACAGACTGCAGGTTCTGGACAACCACTACCAGGACGTGCTCAAGGAGGTTAA

GGCGGCGGCGTCAAAAGTGAAGGCTAACTTGCTATCCGTAGAGGAAGCTTGCAGCCTGAC

GCCCCCACATTCAGCCAAATCCAAATTTGGCTACGGGGCAAAAGACGTCCGTTGCCATGC

CAGAAAGGCCATAAACCACATCAACTCCGTGTGGGAGGACCTTCTGGAAGACAGTGTAAC

ACCAATAGATACTACCATCATGGCTAAGAACGAAGTTTTCTGCGTTCAGCCTGAGAAGGG

GGGTCGTAAGCCAGCTCGACTCATCGTGTTCCCCGACCTGGGTGTGCGCGTGTGCGAGAA

AATGGCCCTATACGACGTAGTCAGCAAACTCCCTATAGCCGTGATGGGAAGCTCCTACGG

ATTCCAATACTCACCAGGGCAGCGGGTTGAATTCCTCGTGCAAGCGTGGAAGTCCAAGAA

GACCCCAATGGGGTTTTCATATGATACTCGCTGTTTTGACTCCACAGTCACTGAGAGTGA

CATCCGTACGGAGGAGGCAATCTACCAGTGTTGTGACCTGGACCCCCAAGCCCGTGTGGC

CATTAAGTCCCTCACCGAGAGGCTTTATGTTGGGGGCCCTCTTACTAACTCAA

>MT454938

CACATTTGACAGACTGCAGGTTCTGGACAACCACTACCAGGACGTGCTCAAGGAGGTTAA

GGCGGCGGCGTCGAAAGTGAAGGCTAACTTGCTATCCGTAGAGGAAGCTTGCAGCCTGAC

GCCCCCACATTCAGCCAAATCCAAATTTGGCTACGGGGCAAAAGACGTCCGTTGCCATGC

CAGAAAGGCCGTAAACCACATCAACTCCGTGTGGAAAGACCTTCTGGAAGACAGTGTAAC

ACCAATAGATACCACCATCATGGCTAAGAACGAAGTTTTCTGCGTTCAGCCTGAAAAGGG

AGGTCGTAAGCCAGCTCGACTCATCGTGTTCCCCGACCTGGGTGTGCGCGTGTGCGAGAA

GATGGCCTTATACGACGTAGTCAGCAAGCTCCCTATAGCCGTGATGGGAAGCTCCTACGG

ATTCCAATACTCACCAGGGCAGCGGGTTGAATTCCTCGTGCAAGCGTGGAAGTCCAAGAA

GACCCCAATGGGGTTTTCGTATGATACCCGCTGCTTTGACTCCACAGTCACTGAGAGCGA

TATCCGTACGGAGGAGGCAATCTACCAGTGTTGTGACCTGGACCCCCAAGCCCGCGTGGC

CATCAAGTCCCTCACCGAGAGGCTTTATGTCGGGGGCCCTCTTACCAATTCAA

>MT454939

CACATTTGACAGACTGCAGGTTCTGGACAACCACTACCAGGACGTGCTCAAGGAGGTTAA

GGCGGCGGCGTCAAAAGTGAAGGCTAACTTGCTATCCGTAGAGGAAGCTTGTAGCCTGAC

GCCCCCACATTCAGCCAGATCCAAATTTGGCTATGGGGCGAAAGACGTCCGTTGCCATGC

CAGAAAGGCCGTAAACCACATCAACTCCGTGTGGGAGGACCTTCTGGAAGACAGTGTAAC

ACCAATAGATACCACCATCATGGCTAAAAACGAGGTCTTTTGCGTTGCGCCTGAGAAGGG

AGGTCGCAAGCCAGCTCGACTCATCGTGTTCCCCGACCTGGGTGTGCGCGTGTGCGAGAA

GATGGCTTTGTACGACGTAGTCAGCAAACTCCCTACGGCCGTGATGGGAAGCTCCTACGG

ATTCCAATACTCACCAGGACAGCGGGTTGAGTTCCTCGTGCAAGCGTGGAAGTCCAAGAG

GACCCCAATGGGGTTCTCGTATGATACCCGCTGCTTTGACTCTACAGTCACTGAGAGCGA

TATCCGTACGGAGGAGGCAATCTACCAGTGTTGTGACCTGGACCCCCAAGCCCGCGTGGC

CATCAAGTCCCTCACTGAGAGGCTTTATGTCGGGGGCCCTCTTACCAACTCAA

>MT454940

CACATTTGACAGACTGCAAGTCCTGGACAGCCATTACCGGGACGTGCTCAAGGAGGTAAA

AGCAGCGGCGTCGACAGTGAAGGCTAACTTGCTATCTGTAGAGGAAGCTTGCAGCCTGAC

GCCCCCACACTCAGCCAGATCCAAGTTTGGCTATGGGGCAAAAGACGTCCGTTGCCATGC

CAGAAAGGCCGTAAACCACATCAACTCCGTGTGGAAAGACCTTCTGGAAGACAGTGTAAC

ACCAATAGACACCACCATCATGGCCAAGAACGAGGTTTTCTGCGTTCAGCCTGAAAAGGG

GGGTCGTAAGCCAGCTCGTCTCATCGTGTACCCCGACCTGGGTGTGCGCGTGTGCGAGAA

GATGGCCCTGTACGACGTGGTCAGCAAGCTCCCCCTGGCCGTGATGGGAAGCTCCTACGG

ATTCCAATACTCACCAGGACAGCGGGTTGAATTCCTCGTGCAAGCGTGGAAGTCCAAGAA

GACTCCAATGGGGTTTTCGTATGATACCCGCTGCTTTGACTCCACAGTCACTGAGAGCGA

CATCCGTACGGAGGAGGCAATCTACCAATGTTGTGACCTGGACCCCCAAGCCCGCGTGGC

TATTAAGTCCCTCACTGAGAGGCTTTACGTTGGGGGCCCTCTTACCAATTCAA

>MT454941

CACATTTGACAGACTGCAGGTTCTGGATAACCACTACCAGGACGTGCTCAAGGAGGTTAA

GGCGGCGGCGTCAAAAGTGAAGGCTAACTTGCTATCCGTGGAGGAAGCTTGCAGCCTGAC

GCCCCCACATTCAGCCAAATCCAAATTTGGCTACGGGGCAAAAGACGTCCGTTGCCACGC

CAGAAAGGCCGTAAACCACATTAACTCCGTGTGGAAAGACCTTCTGGAAGACAGTGTAAC

ACCAATACATACCACCATCATGGCTAAGAACGAAGTTTTTTGCGTTCATCCTGAGAGGGG

GGGTCGTAAGCCAGCTCGCCTCATCGTGTTCCCCGACCTGGGTGTGCGCGTGTGCGAGAA

GATGGCCCTGTACGACGTAGTCAGCAAACTCCCTATAGCCGTGATGGGAAGCTCCTACGG

ATTCCAATACTCACCAGGGCAGCGGGTTGAATTCCTCGTGCAAGCGTGGAAGTCCAAGAA

GACCCCAATGGGGTTTTCGTATGATACCCGCTGCTTTGACTCTACAGTCACTGAGAGCGA

TATCCGTACTGAGGAGGCAATCTACCAGTGTTGTGACCTGGACCCCCAAGCCCGCGTGGT

CATCAAGTCCCTCACCGAGAGGCTTTATGTCGGGGGTCCTCTTACCAATTCAA

>MT454942

CACATTTGACAGACTGCAGGTTCTGGACAATCACTACCAGGACGTGCTCAAGGAGGTTAA

GGCGGCGGCGTCAAAAGTGAAGGCTAACTTGCTATCCGTGGAGGAAGCTTGCAGCCTGAC

GCCCCCACACTCAGCCAAATCCAAATTTGGCTACGGGGCAAAAGACGTCCGTTGCCATGC

CAGAAAGGCCGTAAACCACATCAACTCCGTGTGGAAGGACCTTCTGGAAGACAATGTAAC

ACCAATAGATACTACCATCATGGCTAAGAACGAAGTTTTCTGCGTTCAGCCTGAGAAGGG

GGGTCGTAAGCCAGCTCGACTCATCGTGTTCCCCGACCTGGGTGTGCGCGTGTGCGAGAA

GATGGCCCTGTACGACGTAGTCAGCAAACTCCCCATAGCCGTGATGGGAAGCTCCTACGG

ATTCCAATACTCACCAGGGCAGCGGGTTGAATTCCTCGTGCAAGCGTGGAAATCCAAGAA

GACCCCAATGGGGTTTTCGTATGATACCCGCTGCTTTGACTCTACAGTCACTGAGAGCGA

CATCCGTACGGAGGAGGCAATCTACCAGTGTTGTGACCTGGACCCCCAAGCCCGCGTGGC

CATCAAGTCCCTCACCGAGAGGCTTTATGTCGGGGGCCCTCTTACCAATTCAA

>MT454943

CACATTTGACAGACTGCAGGTTCTGGACAACCACTACCAGGACGTGCTCAAGGAGGTTAA

GGCGGCGGCGTCAAAAGTGAAGGCTAACTTGCTATCTGTAGAGGAAGCTTGCAGCCTGAC

GCCCCCACATTCAGCCAAATCCAAATTTGGCTACGGGGCAAAAGACGTCCGTTGCCATGC

CAGAAAGGCCATAAACCACATCAACTCCGTGTGGAAGGACCTTCTGGAAGACAGTGAAAC

ACCAATAATGACTACCATCATGGCTAAGAACGAAGTTTTCTGCGTTGAGCCTGAGAAGGG

GGGTCGCAAGCCAGCTCGACTCATCGTGTTCCCCGACCTGGGTGTACGCGTGTGCGAGAA

GATGGCTCTGTACGACGTGGTCAGCAAACTCCCTATAGCCGTGATGGGAAGCTCCTACGG

ATTCCAATACTCACCAGGGCAGCGGGTTGAATTCCTCGTGCAAGCGTGGAAGTCCAAGAA

GACCCCAATGGGGTTTTCGTATGATACCCGCTGCTTTGACTCTACGGTCACTGAGAGCGA

TATCCGTACGGAGGAGGCAATCTACCAGTGTTGTGACCTGGACCCCCAAGCCCGCGTGGT

CATCAAGTCCCTCAGCGAGAGGCTTTATGTCGGGGGCCCTCTTACCAATTCAA

>MT454944

CACATTTGACAGACTGCAGGTCCTGGACAACCACTACCAGGACGTGCTCAAGGAGGTTAA

GGCGGCGGCGTCAAAAGTGAAGGCTAACTTGCTATCCGTAGAGGAAGCTTGCAGCCTGAC

GCCCCCACATTCAGCCAGATCCAAATTTGGCTATGGGGCAAAAGACGTCCGTTGCCATGC

CAGAAAGGCCGTAAACCACATCAACTCCGTGTGGGAGGACCTTCTGGAAGACAGCGTAAC

ACCATTACAAACTACCATCATGGCTAAGAACGAAGTTTTCTGCGTTGAGCCTGAGAAGGG

GGGTCGTAAGCCAGCTCGACTCATCGTGTTCCCCGACCTGGGTGTGCGCGTGTGCGAGAA

GATGGCCTTGTACGACGTAGTCAGCAAGCTCCCTATAGCCGTGATGGGAAGCTCCTACGG

ATTCCAATACTCACCAGGACAGCGGGTTGAATTCCTCGTGCAAGCGTGGAAGTCTAAGAA

GACCCCAATGGGGTTTTCGTATGATACCCGCTGCTTTGACTCCACAGTCACTGAGAGCGA

TATCCGTACGGAGGAGGCAATCTACCAGTGTTGTGACCTGGACCCCCAAGCCCGCGTGGC

CATCAAGTCCCTCACCGAGAGGCTTTATGTCGGGGGCCCGCTTACCAATTCAA

>MT454945

CACATTTGACAGACTGCAGGTTCTGGACAACCACTACCAGGACGTGCTTAAGGAGGTTAA

GGCGGCGGCGTCAAAGGTGAAGGCTAACTTGCTATCCGTAGAGGAAGCTTGTAGCCTGAC

GCCCCCACATTCAGCCAAATCCAAATTTGGCTACGGGGCAAAGGACGTCCGCTGCCATGC

CAGAAAGGCCGTAAACCACATCAACTCCGTGTGGGAAGACCTTCTGGAAGACAATGTAAC

ACCAATAGACACTACCATCATGGCTAAGAACGAAGTTTTCTGCGTTCAGCCCGAGAAGGG

GGGTCGTAAGCCAGCTCGACTCATCGTGTTCCCCGACCTGGGTGTGCGCGTGTGCGAGAA

GATGGCTCTGTACGACGTGGTCAGCAACCTCCCCATAGCCGTGATGGGAAGCTCCTACGG

ATTCCAATACTCACCAAAGCAGCGGGTTGAATTCCTCGTGCAAGCGTGGAAGTCCAAGAA

GACCCCAATGGGGTTTTCGTATGATACCCGCTGTTTTGACTCTACAGTCACTGAGCATGA

TATCCGTACGGAGGAGGCAATCTACCAGTGTTGTGACCTGGACCCCCAAGCCCGCGTGGC

CATCAAGTCCCTCACCGAGAGGCTTTATGTCGGGGGCCCTCTTACCAATTCAA

>MT454946

CACATTTGACAGACTGCAGATTCTGGACAACCACTACCAGGACGTGCTCAAGGAGGTTAA

GGCGGCGGCGTCAAAAGTGAAGGCTAACTTGCTATCTGTAGAGGAAGCTTGCTGCCTGAC

GCCCCCACATTCAGCCAAATCCAAATTTGGCTACGGGGCAAAAGACGTCCGTTGCCATGC

CAGAAAGGCCATAAACCACATCAACTCCGTGTGGAAGGACCTTCTGGAAGACAGTGAAAC

ACCAATAATGACTACCATCATGGCTAAGAACGAAGTTTTCTGCGTTGAGCCTGAGAAGGG

GGGTCGCAAGCCAGCTCGACTCATCGTGTTCCCCGACCTGGGTGTACGCGTGTGCGAGAA

GATGGCCCTGTACGACGTGGTCAGCAAACTCCCTATAGCCGTGATGGGAAGCTCCTACGG

ATTCCAATACTCACCAGGGCAGCGGGTTGAATTCCTCGTGCAGGCGTGGAAGTCCAAGAA

GACCCCAATGGGGTTTTCGTATGATACCCGCTGCTTTGACTCTACAGTCACTGAGAGCGA

TATCCGTACGGAGGAGGCAATCTACCAGTGTTGTGACCTGGACCCCCAAGCCCGTGTGGT

CATCAAGTCCCTCAGCGAGAGGCTTTATGTCGGGGGCCCTCTTACCAATTCAA

>MT454947

CACATTTGACAGACTGCAAGTTCTGGACAACCATTACCAGGACGTGCTCAAGGAGGTCAA

AGCAGCGGCGTCAAAAGTGAAGGCTAACTTGCTATCCGTAGAGGAAGCTTGCAGCCTGAC

GCCCCCACATTCAGCCAAATCCAAGTTTGGCTATGGGGCAAAAGACGTCCGTTGCCATGC

CAGAAAGGCCGTAGCCCACATCAACTCCGTGTGGAAAGACCTTCTGGAAGACAGTGTAAC

ACCAATAGACACTACCATCATGGCTAAGAACGAGGTTTTCTGCGTTCAGCCTGAGAAGGG

GGGTCGCAAGCCAGCTCGTCTCATCGTGTTCCCTGACCTGGGCGTGCGTGTGTGCGAGAA

GATGGCCCTGTACGACGTGGTTAGCAAGCTCCCCCTGGCCGTGATGGGAAGCTCCTACGG

ATTCCAGTACTCACCAGGACAGCGGGTTGAATTCCTCGTGCAAGCATGGAGGTCCAAGAA

AACCCCAATGGGGTTCTCGTATGACACCCGCTGTTTTGACTCCACAGTTACTGAGAATGA

CATCCGTACGGAGGAGGCAATTTACCAATGTTGTGACCTGGACCCCCAAGCCCGCGTGGC

CATCAAGTCCCTCACTGAGAGGCTTTATGTTGGGGGCCCTCTTACCAATTCAA

>MT454948

CACATTTGACAGACTACAAGTTCTGGACAACCACTACCAGGACGTGGTCAAGGAGGTCAA

GGCGGCGGCGTCAAAAGTGAAGGCTAACTTGCTATCCGTAGAGGAAGCTTGCAGCCTGAC

GCCCCCACATTCAGCCAAATCTAAATTTGGCTACGGGGCAAAAGACGTCCGTTGCCATGC

CAGAAAGGCCGTGGCCCACATCAACTCCGTGTGGAAAGACCTTCTGGAAGACAGTGTAAC

ACCAATAGATACTACCATCATGGCTAAGAACGAAGTTTTCTGCGTTCAGCCTGAGAAGGG

GGGTCGTAAGCCAGCTCGACTCATCGTGTTCCCCGATCTGGGTGTGCGCGTGTGCGAGAA

GATGGCCCTGTACGACGTAGTCAGCAAGCTCCCTATAGCCGTGATGGGAAGCTCCTACGG

ATTCCAATACTCACCAGGACAGCGGGTTGAATTCCTCGTGCAAGCGTGGAAGTCCAAGAA

GACCCCAATGGGGTTTTCGTATGATACCCGCTGCTTTGACTCCACAGTCACTGAGAGCGA

TATCCGTATGGAGGAGACAATCTACCAGTGTTGTGACCTGGACCCCCAGGCCCGCGTGGC

CATTAAGTCCCTCACCGAGAGGCTTTATGTCGGGGGTCCTCTTACCAATTCAA

>MT454949

CACATTTGACAGACTGCAGGTCCTGGATAACCATTACCATGACGTGCTAAAGGAGGTTAA

GGCGGCGGCGTCAAAAGTGAAGGCTAACTTGCTATCCGTGGAGGAAGCTTGCAGCCTGAC

GCCCCCACATTCAGCCAGATCCAAATTTGGCTATGGGGCAAAAGACGTCCGCTGCCACGC

CAGAAAGGCCGTAAACCACATCAGCTCCGTGTGGAAAGACCTTCTGGAAGACAGTGTAAC

ACCAATAGACACTACCATCATGGCTAAGAACGAAGTTTTCTGCGTCCAGCCTGAAAAGGG

AGGTCGTAAGCCAGCTCGACTCATCGTGTTCCCCGACCTGGGTGTGCGCGTGTGCGAGAA

GATGGCCCTGTACGACGTAGTCAGCAAACTCCCTATTGCCGTGATGGGAAGCTCCTACGG

ATTCCAATACTCACCAAAGCAGCGGGTTGAATTCCTCGTGCAAGCGTGGAAGTCCAAGAA

GACCCCAATGGGTTTCTCGTATGATACCCGCTGCTTTGACTCTACAGTCACCGAGAGCGA

TATCCGTACGGAGGAGGCAATCTACCAATGTTGTGACCTGGACCCCCAAGCCCGTGTGGC

CATCAAGTCCCTCACTGAGAGGCTTTATGTCGGGGGCCCTCTTACCAATTCAA

>MT454950

CACATTTGACAGACTGCAGGTTCTGGACAATCACTACCAGGACGTGCTCAAGGAGGTTAA

GGCGGCGGCGTCAAAAGTGAAGGCTAACTTGCTGTCCGTAGAGGAAGCTTGCAGCCTGAC

GCCCCCACATTCAGCCAAATCCAAATTTGGCTACGGGGCAAAAGACGTCCGTTGCCATGC

CAGAAAGGCCGTAAACCACATCAACTCCGTGTGGAAGGACCTTCTGGAAGACAGTGTAAC

ACCAATAGACACTACCATCATGGCTAAGAACGAAGTTTTCTGCGTTCAGCCTGAAAAGGG

AGGTCGTAAGCCGGCTCGACTCATCGTGTTCCCCGACCTGGGTGTGCGCGTGTGCGAGAA

GATGGCCCTGTACGACGTGGTCAGCAAACTCCCTATAGCCGTGATGGGAAGCTCCTACGG

ATTCCAATACTCACCAGGGCAGCGGGTTGAATTCCTCGTGCAAGCGTGGAAGTCCAAGAA

GACCCCAATGGGGTTTTCGTATGATACCCGCTGCTTTGACTCTACAGTCACTGAGAGCGA

TATCCGTACGGAGGAGGCAATCTACCAGTGTTGTGACCTGGACCCCCAAGCCCGCGTGGC

CATCAAGTCCCTCACCGAGAGACTTTATGTCGGGGGCCCTCTTACCAATTCAA

>MT454951

CACATTTGACAGACTGCAAGTTCTGGACAACCACTACCAGGACGTGCTCAAGGAGGTTAA

GGCGGCGGCGTCAAAAGTGAAGGCCAACTTGCTATCCGTAGAGGAAGCATGTAGCCTGAC

GCCCCCACATTCAGCCAAATCCAAATTTGGCTACGGGGCAAAGGACGTCCGCTGCCATGC

CAGAAAGGCCGTAAACCACATCAACTCCGTGTGGAAGGACCTTCTGGAAGACAGTGTAAC

ACCAATAGATACTACCATCATGGCTAAGAACGAAGTTTTCTGCGTTCAGGCTGAGAAGGG

GGGTCGTAAGCCAGCTCGACTCATCGTGTTCCCCGACCTGGGTGTGCGCGTGTGCGAGAA

GATGGCTTTGTACGACGTAGTCAGCAAACTCCCTGTAGCCGTGATGGGAAGCTCCTACGG

ATTCCAATACTCACCAGGGCAGCGGGTTGAATTCCTCGTGCAAGCGTGGAAGTCCAAGAA

GACCCCAATGGGGTTTTCGTATGATACCCGCTGCTTTGACTCTACAGTCACTGAGAGCGA

CATCCGTACGGAGGAGGCAATCTACCAATGTTGTGACTTGGACCCCCAAGCCCGCGTGGC

CATTAAGTCCCTCACCGAGAGGCTTTATGTCGGGGGTCCTCTTACCAATTCAA

>MT454952

CACATTTGACAGACTGCAGGTTCTGGACAGCCACTACCAGGACGTGCTCAAGGAGGTTAA

GGCAGCGGCGTCAAAAGTGAAGGCTAATTTGCTATCCGTAGAGGAAGCTTGCAGCCTGAC

GCCCCCACATTCAGCCAGATCCAAATTTGGCTACGGGGCAAAAGACGTCCGTTGCCACGC

CAGAAAGGCCGTAAGCCACATCAACTCCGTGTGGAAAGACCTTCTGGAAGACAGTGTAAC

ACCAATAGATACTACCATCATGGCTAAGAACGAAGTTTTCTGCGTTCAGCCTGAAAAGGG

AGGTCGTAAGCCAGCTCGACTCATCGTGTACCCCGACCTGGGTGTGCGCGTGTGCGAGAA

AATGGCCCTGTACGACGTAGTCAGCAAACTCCCCATAGCCGTGATGGGAAGCTCCTACGG

ATTCCAATACTCACCAGCGCAGCGGGTTGAATTCCTCGTGCAAGCGTGGAAGTCCAAGAA

GACCCCAATGGGGTTTTCGTATGATACCCGCTGCTTTGACTCTACAGTCACTGAGAGCGA

TATCCGTACGGAGGAGGCAATCTACCAATGTTGTGACCTGGACCCCCAAGCCCGCGTGGC

CATCAAGTCTCTCACCGAGAGGCTTTATGTCGGGGGCCCTCTTACCAATTCAA

>MT454953

CACATTTGACAGACTGCAGGTTCTGGACAATCACTACCAGGACGTGCTCAAGGAGGTTAA

GGCGGCGGCGTCAAAAGTGAAGGCTAACTTGCTATCCGTAGAGGAAGCTTGCAGCCTGAC

GCCCCCACATTCAGCCAGATCCAAATTTGGCTATGGGGCAAAAGACGTCCGCTGCCATGC

CAGGAAGGCCGTATCCCACATCAACTCCGTGTGGAAGGACCTTCTGGAAGACTCTGTAAC

ACCAATAGATACTACCATCATGGCTAAGAACGAAGTTTTCTGTGTTCAGCCTGAGAAGGG

AGGTCGTAAGCCAGCTCGACTCATCGTGTTTCCCGACCTGGGTGTGCGCGTGTGCGAGAA

GATGGCCCTGTACGACGTAGTCAGCAAACTCCCTACAGCCGTGATGGGCAGCTCCTACGG

ATTCCAATACTCACCAGGGCAGCGGGTTGAATTCCTCGTGCAAGCGTGGAAGCACAAGAA

GACCCCAATGGGGTTTTCGTATGACACCCGCTGCTTTGACTCTACAGTCACTGAGAGCGA

TATCCGTACGGAGGAGGCAATCTACCAGTGTTGTGACCTGGACCCCCAAGCCCGCGTGGC

CATCAAGTCCCTCACCGAGAGGCTTTATGTCGGGGGCCCTCTTACCAATTCAA

>MT454954

CACATTTGACAGACTGCAGGTCCTGGACAACCACTACCAGGACGTGCTCAAGGAGGTTAA

GGCGGCGGCGTCAAAAGTGAAGGCTAACTTGCTATCCGTAGAGGAAGCTTGTAGCCTGAC

GCCCCCACATTCAGCCAGATCTAAATTTGGCTATGGGGCAAAGGACGTCCGCAGCCATGC

CAGAAAGGCCGTAAACCACATCAACTCCGTGTGGGCAGACCTTCTGGAAGACAGTGTAAC

ACCAATAGATACTACCATCATGGCTAAGAACGAAGTCTTCTGCGTTCAGCCTGAGAAGGG

GGGTCGTAAGCCAGCTCGTCTCATCGTGTTCCCCGACCTGGGTGTGCGCGTGTGCGAGAA

GATGGCCCTGTACGACGTGGTCAGCAAGCTCCCTGTGGCCGTGATGGGAAGCTCCTACGG

ATTTCAATACTCACCAGGACAGCGGGTTGAATTCCTCGTGCAAGCGTGGAAATCCAAGAA

GACCCCAATGGGGTTTTCATATGACACCCGCTGCTTTGACTCCACAGTCACTGAGAACGA

TATCCGTACGGAGGAGGCAATCTACCAGTGTTGTGACCTGGACCCCCAAGCCCGCGTGGC

CATTAAGTCCCTCACCGAGAGGCTTTATGTTGGGGGCCCTCTTACCAATTCAA

>MT454955

AACATTTGACAGACTGCAGGTTCTGGACAACCACTACCAGGACGTGCTCAAGGAGGTCAA

GGCGGCGGCGTCAAAAGTGAAGGCTAACTTGCTATCCGTAGAGGAAGCTTGTGACCTGAC

GCCCCCACATTCAGCCAAATCCAAATTTGGCTATGGGGCAAAAGACGTCCGTTGCCATGC

CAGAAAGGCCGTAAACCACATCAACTCCGTGTGGAAAGACCTTCTGGAAGACAGTGTAAC

ACCAATAGATACTACCATCATGGCTAAGAACGAAGTTTTCTGCGTTCAGCCTGAGAAGGG

AGGTCGTAAGCCAGCTCGACTCATCGTGTTCCCCGACCTGGGTGTGCGCGTGTGCGAGAA

GATGGCCCTATACGACGTAGTCAGCAAACTCCCTATAGCCGTGATGGGAAGCTCCTACGG

ATTCCAATACTCACCAGGGCAGCGGGTTGAATTCCTCGTGCAAGCGTGGAAGTCCAAGAA

GACCCCAATGGGGTTTTCGTATGATACCCGCTGCTTTGACTCCACAGTCACTGAGAGCGA

TATCCGTACGGAGGAGGCAATCTACCAATGTTGTGACCTGGACCCCCAAGCCCGCGTGGC

CATCAAGTCCCTCACCGAGAGGCTTTATGTCGGGGGCCCTCTTACCAATTCAA

>MT454956

CACATTTGACAGACTGCAAGTTCTGGACAACCACTACCAGGACGTGCTCAAGGAGGCTAA

GGCGGCGGCGTCAAAAGTGAAGGCTAATTTGCTATCCGTAGAGGAAGCTTGCAGCCTGAC

GCCCCCACATTCAGCCAGATCTAAATTTGGCTATGGGGCAAAAGACGTCCGTTGCCATGC

CAGAAAGGCCGTAGCCCACATCAACTCCGTGTGGAAGGACCTTCTGGAAGACAGTGTAAC

ACCAATAGATACTACCATCATGGCTAAGAACGAGGTTTTCTGCGTTCAGCCTGAAAAGGG

GGGTCGTAAGCCAGCTCGACTCATCGTGTTCCCCGACTTGGGTGTGCGCGTGTGCGAGAA

GATGGCCCTGTACGACGTAGTCAGGAAACTCCCCATAGCCGTGATGGGAAGCTCCTACGG

ATTCCAATACTCGCCAGGGCAGCGGGTTGAATTCCTCGTGCAAGCGTGGAAGTCCAAGAA

GACCCCAATGGGGTTTTCGTATGATACCCGCTGCTTTGACTCCACAGTCACCGAGAGCGA

CATCCGTACGGAGGAGGCAATCTACCAGTGTTGTGACCTGGACCCCCAAGCCCGCGTGGC

CATCAAGTCCCTCACAGAGAGGCTTTATGTCGGGGGTCCCCTTACCAATTCAA

>MT454957

CACATTTGACAGACTGCAAGTTCTGGACAACCACTACTATGAAGTGGTTAAGGAGGTTAA

GGCGGCGGCGTCAAAAGTGAAGGCTAACTTGCTATCCGTAGAGGAAGCTTGCAGCCTGAC

GCCCCCACATTCAGCTAAATCTAAATTTGGCTACGGGGCAAAAGACGTCCGCTGCCATGC

CAGAAAGGCCGTAAACCACATCAACTCCGTGTGGGAGGACCTTCTGGAAGACAGTGTAAC

ACCAATAGATACTACCATCATGGCCAAGAACGAAATTTTCTGTGTTCAGCCTGAGAAAGG

GGGTCGTAAGCCAGCTCGACTCATCGTGTTCCCCGACCTGGGTGTGCGCGTGTGCGAGAA

GATGGCCCTGTACGACGTCATCAGGAAACTCCCCACGGCCGTGATGGGAAGCTCCTACGG

ATTCCAATATTCACCAGGACAGCGGGTTGAATTTCTCGTGAAAGCGTGGAATTCCAAGAG

GATCCCAATGGGGTTCTCGTATGATACCCGCTGCTTTGACTCTACAGTCACTGAGAGCGA

CATCCGTACGGAGGAGGCAATCTACCAGTGCTGTGACCTGGACCCCCAAGCCCGTATAGC

CATCAGGTCCCTCACCGAGAGGCTTTACGTTGGGGGCCCTCTCACCAATTCAA

>MT454958

CACATTTGACAGACTGCAAGTCCTGGATAGCCATTACCGGGACGTGCTCAAGGAGGTTAA

GGCAGCGGCGTCAAAGGTGAAGGCTAACTTGCTATCCGTAGAGGAGGCTTGCAGCCTGAC

GCCCCCACACTCAGCCAGATCCAAGTTTGGCTATGGGGCAAAAGACGTCCGTTGCCATGC

CAGAAAGGCCGTAAACCACATCAACTCCGTGTGGAAAGACCTTCTGGAAGACAGTGTAAC

ACCTATAGACACTACCATCATGGCTAAGAACGAGGTTTTCTGCGTTCAGCCTGAGAAGGG

GGGTCGCAAGCCAGCTCGTCTCATCGTGTACCCCGATCTGGGTGTGCGCGTGTGCGAGAA

GATGGCCCTGTACGACGTGGTTAGCAAGCTCCCCGTAGCCGTGATGGGAGACTCCTACGG

ATTCCAATACTCACCAGGACAGCGGGTTGAATTCCTCGTGCAAGCGTGGAAGTCCAAGAA

GACCCCGATGGGGTTCTCGTATGATACCCGCTGCTTTGACTCCACAGTCACTGAGAGCGA

CATCCGTACGGAGGAGGAAATCTACCAATGTTGTGACCTGGACCCCCAAGCCCGCGTGGC

CATCAAGTCCCTTACTGAGAGGCTTTATGTCGGGGGCCCGCTTACCAATTCAA

>MT454959

CACATTTGACAGACTGCAGGTTCTGGACAACCACTACCAGGACGTGCTCAAGGAGGTCAA

GGCGGCGGCGTCAAAAGTGAAGGCTAACTTGCTATCCGTAGAGGAAGCTTGTGACCTGAC

GCCCCCACATTCAGCCAAATCCAAATTTGGCTACGGGGCAAAAGACGTCCGTTGCCATGC

CAGAAAGGCCGTAAACCACATCAGCTCCGTGTGGAAAGACCTTCTGGAAGACAGTGTAAC

ACCAATAGACACTACCATCATGGCTAAGAACGAAGTTTTTTGCGTTCAGCCCGAGAAGGG

AGGTCGTAAGCCAGCTCGACTCATCGTGTTCCCCGACCTGGGTGTGCGCGTGTGCGAGAA

GATGGCCCTGTACGACGTAGTCAGCAAACTCCCTATAGCCGTGATGGGAAGCTCCTACGG

ATTCCAATACTCACCAGGGCAGCGGGTTGAATTCCTCGTGCAAGCGTGGAAGTCCAAGAA

GACCCCAATGGGGTTTTCATATGATACCCGCTGCTTTGACTCCACAGTCACTGAGAGCGA

TATCCGTACGGAGGAGGCAATCTACCAGTGTTGTGACCTGGACCCCCAAGCCCGTGTGGC

CATCAAGTCTCTCACCGAGAGGCTTTATGTCGGGGGCCCTCTTACCAATTCAA

>MT454960

CACATTTGACAGACTGCAGGTTCTGGATAGCCACTACCAGGACGTGCTCAAGGAGGTTAA

GGCGGCGGCGTCAAAAGTGAAGGCTAACTTGCTATCCGTAGAGGAAGCTTGCAGCCTGAC

GCCCCCACACTCAGCCAAATCCAAATTTGGCTATGGGGCAAAAGATGTCCGTTGCCATGC

CAGAAGGGCCGTGGACCACATCAACTCCGTGTGGAAGGACCTTCTGGAAGACAGTGTAAC

ACCAATAGATACTACCATCATGGCTAAGAACGAAGTTTTCTGCGTTCAGCCTGAGAAGGG

AGGTCGTAAGCCAGCTCGACTCATCGTGTTCCCGGACCTGGGTGTACGCGTGTGCGAGAA

AATGGCCCTGTACGACGTGGTCAGCAAACTCCCTATAGCTGTGATGGGAAGCTCCTACGG

ATTCCAATACTCACCAGGCCAGCGGGTTGAATTCCTCGTGCAAGCGTGGAAGTCCAAGAA

GACCCCAATGGGGTTTTCGTATGATACCCGCTGCTTTGACTCCACAGTCACTGAGAGCGA

TATCCGTACGGAGGAGGCAATCTACCAGTGTTGTGACCTGGACCCCCAAGCCCGCGTGGC

CATCAAGTCCCTCACCGAGAGGCTTTATGTCGGGGGTCCTCTTACCAATTCAA

>MT454961

CACATTTGACAGACTGCAAGTTCTGGACAACCATTACCAGGACGTGCTCAAGGAGGTTAA

AGCAGCGGCGTCGAGAGTGAAGGCTAACTTGCTGTCCGTAGAGGAAGCTTGCAGCCTGAC

GCCCCCACATTCAGCCAAGTCCAAATTTGGCTATGGGGCAAAAGACGTCCGTTGCCATGC

CAGAAAGGCCGTAAACCACATCAACTCCGTGTGGAAAGACCTTCTGGAAGACAGTGTAAC

ACCAATAGATACTACCATCATGGCTAAGAACGAAGTTTTCTGCGTTCAGCCTGAGAAGGG

AGGTCGTAAGCCAGCTCGACTCATCGTGTTCCCCGACCTGGGTGTGCGCGTGTGCGAGAA

GATGGCTCTGTACGACGTAGTCAGCAAACTCCCTATAGCCGTGATGGGAAGCTCCTACGG

ATTCCAATACTCACCAGGGCAGCGGGTTGAATTCCTCGTGCAAGCGTGGAAGTCCAAGAA

GACCCCAATGGGGTTTTCGTATGATACCCGCTGCTTTGACTCTACAGTCACTGAGAGCGA

TATCCGTACGGAGGAGGCAATCTACCAATGTTGTGACCTGGACCCCCAAGCCCGCGTGGC

CATCAAGTCCCTCACCGAGAGACTTTATGTCGGGGGCCCTCTTACCAATTCAA

>MT454962

CACATTTGACAGACTGCAGGTTCTGGACAACCACTACCAGGACGTGCTCAAGGAGGTTAA

GGCAGCGGCGTCAAAAGTGAAGGCTAACTTGCTATCCGTAGAGGAAGCTTGCAGCCTGAC

GCCCCCACATTCAGCCAAATCCAAATTTGGCTACGGGGCGAAAGACGTCCGTTGCCATGC

CAGAAAGGCCGTAAACCACATCAACTCCGTGTGGGAAGACCTTCTGGAAGACAGTGTAAC

ACCAATAGATACTACCATCATGGCTAAGAACGAAGTTTTTTGCGTTCAGCCTGAGAAGGG

AGGTCGTAAGCCAGCTCGACTCATCGTGTTCCCCGACCTGAGCGTGCGCGTGTGCGAGAA

GATGGCACTGTACGACGTAGTCAGCAAACTCCCTATAGCCGTGATGGGAAGCTCCTACGG

ATTCCAATACTCACCAGGGCAGCGGGTTGAATTCCTCGTGCAAGCGTGGAAGTCCAAGAA

GACCCCAATGGGGTTTTCGTATGATACCCGCTGCTTTGACTCTACAGTCACTGAGAGCGA

TATCCGTACGGAGGAGGCAATCTACCAATGTTGTGACCTGGACCCCCAAGCCCGCGTGGC

CATCAAGTCCCTCACCGAGAGGCTTTATGTCGGGGGCCCTCTTACCAATTCAA

>MT454963

CACATTTGACAGACTGCAGGTTCTGGACAACCATTACCAGGACGTGCTCAAGGAGGTTAA

GGCGGCGGCGTCAAAGGTGAAGGCTAACTTGCTATCCGTAGAGGAAGCTTGCAGCCTGAC

GCCCCCACATTCAGCCAAATCCAAATTTGGCTACGGGGCAAAAGACGTCCGTTGCCATGC

CAGAAAGGCCATAAACCACATCAACTCCGTGTGGAAGGACCTTCTGGAAGACAGTGTAAC

ACCAATACAAACTACCATCATGGCTAAGAACGAAGTTTTTTGCGTTCAGCCTGAGAAAGG

GGGTCGCAAGCCAGCTCGACTCATCGTGTTCCCCGACCTGGGTGTGCGCGTGTGCGAGAA

GATGGCCCTGTACGACGTAGTCAGCAAACTCCCTATAGCCGTGATGGGAAACTCCTACGG

ATTCCAATACTCACCAGGGCAGCGGGTTGAATTCCTCGTGCAAGCGTGGAAGTCCAAGAA

GACCCCGATGGGGTTTTCGTATGATACCCGCTGCTTTGACTCTACAGTCACTGAGAGCGA

TATCCGTACGGAGGAGGCAATCTACCAGTGTTGTGACCTGGACCCCCAAGCCCGTGTGGC

CATCAAGTCCCTCACCGAGAGGCTTTATGTTGGGGGCCCTCTTACCAATTCAA

>MT454964

CACATTTGACAGACTGCAAGTCCTGGATAGCCATTACCAGGACGTGCTTAAGGAGGTTAA

GGCAGCGGCGTCAAAGGTGAAGGCCAACTTGCTATCCGTGGAGGAAGCTTGTAGCCTGAC

GCCCCCACATTCAGCCAGATCCAAGTTTGGCTATGGGGCAAAAGACGTCCGTTGCCATGC

CAGAAAGGCCGTAAACCACATCAACTCCGTGTGGAAAGACCTTCTGGAAGACAGTGTAAC

ACCTATAGACACCACCATCATGGCTAAGAACGAAGTTTTTTGCGTTCAGCCTGAGAAGGG

GGGCCGCAAGCCAGCTCGTCTCATCGTGTACCCCGATCTGGGTGTGCGCGTGTGCGAGAA

AATGGCTTTATACGACCTGGTCAGCAAGCTCCCCCTGGCCGTGATGGGAAGCTCCTACGG

GTTCCAATACTCACCAGGACAGCGGGTTGAATTCCTCGTGCAAGCGTGGAAGTCCAAGAA

GACCCCGATGGGGTTCTCGTATGATACCCGCTGCTTTGACTCCACAGTCACTGAGAGCGA

CATCCGTACGGAGGAGGCAATCTACCAATGTTGTGACCTGGACCCCCAAGCCCGCGTGGC

CATCAAGTCTCTCACTGAGAGGCTTTATGTTGGGGGCCCGCTTACCAATTCAA

>MT454965

CACATTTGACAGACTGCAGGTTCTGGACAGCCACTACCAGGACGTGCTCAAGGAGGTCAA

GGCGGCGGCGTCAAAAGTGAAGGCTAACTTGCTATCCGTAGAGGAAGCTTGCAGCCTGAC

GCCCCCACACTCAGCCAAATCCAAGTTTGGCTACGGGGCAAAAGACGTCCGTTGCCATGC

CAGAAAGGCCGTAAGCCACATCAACTCCGTGTGGAAGGACCTTCTGGAAGACAGTGTAAC

ACCAATAGACACCACCATCATGGCTAAGAACGAAGTTTTCTGCGTTCAGCCTGAGAAGGG

AGGTCGTAAGCCAGCTCGACTCATCGTGTTCCCCGACCTGGGTGTGCGCGTGTGTGAGAA

GATGGCCCTATACGACGTAGTCAGCAAACTCCCTTTAGCCGTGATGGGAAGCTCCTACGG

ATTCCAATACTCACCAGGACAGCGGGTTGAATTCCTCGTGCAAGCGTGGAAGTCCAAGAA

GACCCCAATGGGGTTTTCGTATGATACCCGCTGCTTTGACTCCACAGTCACTGAGAGCGA

TATCCGTACGGAGGAGGCAATCTACCAGTGTTGTGACCTGGACCCCCAAGCCCGCGTGGC

CATCAAGTCCCTCACCGAGAGGCTTTATGTCGGGGGCCCTCTTATCAATTCAA

>MT454966

CACATTTGACAGACTGCAGGTTCTGGACGACCATTACCAGGACGTGCTCAAGGAGGTCAA

GGCGGCGGCGTCAAAAGTGAAGGCCAACTTGCTATCCGTAGAGGAAGCTTGCAGCCTGAC

GCCCCCACATTCAGCCAAATCCAAATTTGGCTATGGGGCAAAAGACGTCCGTTGCCATGC

CAGAAAGGCCGTAAACCACATCGACTCCGTGTGGAAAGACCTTCTGGAAGACAGTGTAAC

ACCAATAGATACTACCATCATGGCTAAGAACGAAGTGTTCTGCGTTCAGCCTGAAAAGGG

GGGTCGTAAGCCAGCTCGACTCATCGTGTTCCCCGACCTGGGTGTGCGCGTGTGCGAGAA

GATGGCCTTATACGACGTAGTCAGCAAACTCCCTATAGCCGTGATGGGAAGCTCCTACGG

ATTCCAATACTCACCAGGGCAGCGGGTTGAATTCCTCGTGCAAGCGTGGAAGTCCAAGAA

GACCCCAATGGGGTTTTCGTATGATACCCGCTGCTTTGACTCCACAGTCACTGAGAGCGA

TATCCGTACGGAGGAGGCAATCTACCAATGTTGTGACCTGGACCCCCAAGCCCGCGTGGC

CATCAAGTCCCTCACCGAGAGGCTTTATGTGGGGGGCCCTCTTACCAATTCAA

>MT454967

CACATTTGACAGACTGCAGGTTCTGGACAACCACTACCAGGACGTGCTCAAGGAGGTCAA

GGCGGCGGCGTCAAAAGTGAAGGCTAACTTGCTATCCGTAGAGGAAGCTTGTGACCTGAC

GCCCCCACATTCAGCCAAATCCAAATTTGGCTACGGGGCAAAAGACGTCCGTTGCCATGC

CAGAAAGGCCGTAAACCACATCAGCTCCGTGTGGAAAGACCTTCTGGAAGACAATGTAAC

ACCAATAGACACTACCATCATGGCTAAGAACGAAGTTTTCTGCGTTCAGCCCGAGAAGGG

AGGTCGTAAGCCAGCTCGACTCATCGTGTTCCCCGACCTGGGTGTGCGCGTGTGCGAGAA

GATGGCCCTGTACGACGTAGTCAGCAAACTCCCTATAGCCGTGATGGGAAGCTCCTACGG

ATTCCAATACTCACCAGGGCAGCGGGTTGAATTCCTCGTGCAAGCGTGGAAGTCCAAGAA

GACCCCAATGGGGTTTTCATATGATACCCGCTGCTTTGACTCCACAGTCACTGAGAGCGA

TATCCGTACGGAGGAGGCAATCTACCAGTGTTGTGACCTGGACCCCCAAGCCCGTGTGGC

CATCAAGTCCCTCACCGAGAGGCTTTATGTCGGGGGCCCTCTTACCAATTCAA

>MT454968

CACATTTGACAGACTGCAAGTTCTGGACAACCACTACCAGGACGTGCTCAAGGAGGTTAA

AGCGGCGGCGTCGAGAGTGAAGGCTAACTTGCTGTCCGTAGAGGAAGCTTGCAGCCTGAC

GCCCCCACATTCAGCCAAATCCAAATTTGGCTATGGGGCAAAAGACGTCCGTTGCCATGC

CAGAAAGGCCGTAAACCACATCAACTCCGTGTGGAAAGACCTTCTGGAAGACAGTGTAAC

ACCAATAGATACTACCATCATGGCTAAGAACGAAGTTTTCTGCGTTCAGCCTGAGAAGGG

AGGTCGTAAGCCAGCTCGACTCATCGTGTTCCCCGACCTGGGTGTGCGCGTGTGCGAGAA

GATGGCTCTGTACGACGTAGTCAGCAAACTCCCTATAGCCGTGATGGGAAGCTCCTACGG

ATTCCAATACTCACCAGGGCAGCGGGTTGAATTCCTCGTGCAAGCGTGGAAGTCCAAGAA

GACCCCAATGGGGTTTTCGTATGATACCCGCTGCTTTGACTCTACAGTCACTGAGAGCGA

TATCCGTACGGAGGAGGCAATCTACCAATGTTGTGACCTGGACCCCCAAGCCCGCGTGGC

CATCAAGTCCCTCACCGAGAGACTTTATGTCGGGGGCCCTCTTACCAATTCAA

>MT454969

CACATTTGACAGACTGCAGGTCCTGGATAACCACTACCAGGACGTGCTCAAGGAGGTCAA

GGCGGCGGCGTCAAAAGTGAAGGCTAACTTGCTATCCGTAGAGGAAGCTTGTAGCCTGAC

GCCCCCACATTCAGCCAAATCCAAATTTGGCTACGGGGCGAAGGACGTCCGTTGCCATGC

CAGAAAGGCCGTAAACCACATCAACTCCGTGTGGGAAGACCTTCTGGAAGACAGTGTAAC

ACCAATAGACACTACCATCATGGCTAAGAACGAAGTCTTCTGCGTTCAGCCTGAGAAGGG

GGGTCGTAAGCCAGCTCGGCTCATTGTGTTCCCCGACCTGGGTGTGCGCGTGTGCGAGAA

GATGGCCCTGTACGACGTAGTCAGCAAACTCCCTGTGGCCGTGATGGGAAGCTCCTACGG

ATTTCAATACTCGCCGGGACAGCGGGTTGAATTCCTCGTGCAAGCGTGGAAGTCCAAGAA

GACCCCAATGGGGTTTTCGTATGATACCCGCTGTTTTGACTCAACAGTCACTGAGAGCGA

TATCCGTACAGAGGAGGCAATCTACCAGTGTTGTGACCTGGACCCCCAAGCCCGCGTGGC

CATCAAGTCCCTCACCGAGAGGCTTTATGTCGGGGGCCCTCTTACCAATTCAA

>MT454970

CACATTTGACAGACTGCAGGTCCTGGATAGCCACTACCAGGACGTGCTCAAGGAGGTTAA

GGCGGCGGCGTCAAAAGTGAAGGCTAACTTGCTATCCGTAGAGGAAGCTTGCAGCCTGAC

GCCCCCACATTCAGCCAAGTCCAAATTTGGCTATGGGGCAAAAGATGTCCGTTGCCATGC

CAGAAGGGCCGTAGACCACATCAACTCCGTGTGGAAGGACCTTCTGGAAGACAGTGTAAC

ACCAATAGATACTACCATCATGGCTAAGAACGAAGTTTTCTGCGTTCAGCCTGAGAAGGG

GGGTCGTAAGCCAGCTCGACTCATCGTGTTCCCCGACCTGGGTGTGCGCGTGTGCGAGAA

AATGGCCCTGTACGACGTGGTCAGCAAACTCCCTATAGCTGTGATGGGAAGCTCCTACGG

ATTCCAATACTCACCAGGCCAGCGGGTTGAATTCCTCGTGCAAGCGTGGAAGTCCAAGAA

GACTCCAATGGGGTTTTCGTATGATACCCGCTGCTTTGACTCCACAGTCACTGAGAGCGA

TATCCGTACGGAGGAGGCAATTTACCAGTGTTGTGACCTGGACCCCCAAGCCCGCGTGGC

CATCAAGTCCCTCACCGAGAGGCTTTATGTCGGGGGCCCCCTTACCAATTCAA

>MT454971

CACATTTGACAGACTGCAGGTTCTGGATAGCCACTACCAGGACGTGCTCAAGGAGGTTAA

AGCAGCGGCGTCAAGAGTGAAGGCCAACTTGCTATCCGTAGAAGAAGCTTGTAGCCTGAC

GCCCCCACATTCAGCCAAATCCAAATTTGGCTATGGGGCAAAAGATGTCCGTTGCCATGC

CAGAAGGGCCGTAGACCACATCAACTCCGTGTGGAAGGACCTTCTGGAAGACAGTGTAAC

ACCAATAGATACTACCATCATGGCTAAGAACGAAGTTTTCTGCGTTCAGCCTGAGAAGGG

GGGTCGTAAGCCAGCTCGACTCATCGTGTTCCCCGACCTGGGTGTGCGCGTGTGCGAGAA

AATGGCCCTGTACGACGTGGTCAGCAAACTCCCTATAGCCGTGATGGGAAGCTCCTACGG

ATTCCAATACTCACCAGGGCAGCGGGTTGAATTCCTCGTGCAAGCGTGGAAGTCCAAGAA

GACCCCAATGGGGTTTTCGTATGATACCCGCTGCTTTGACTCCACAGTCACTGAGAGCGA

TATCCGTACGGAGGAGGCAATCTACCAATGTTGTGACCTGGACCCCCAAGCCCGCGTGGC

CATCAAGTCCCTCACCGAGAGGCTTTATGTCGGGGGCCCTCTTACCAATTCAA

>MT454972

CACATTTGACAGACTGCAGGTTCTGGACAATCACTACCAGGACGTGCTCAAGGAGGTCAA

GGCGGCGGCGTCAAAAGTGAAGGCTAACTTGCTATCCGTAGAGGAAGCTTGTGACCTGAC

GCCCCCACATTCAGCCAAATCCAAATTTGGCTATGGGGCAAAAGACGTCCGTTGCCATGC

CAGAAAGGCCGTAAACCACATCAACTCCGTGTGGAAAGACCTTCTGGAAGACAGTGTAAC

ACCAATAGACACTACCATCATGGCTAAGAACGAAGTTTTCTGCGTTCAGCCTGAGAAGGG

AGGTCGTAAGCCAGCTCGACTCATCGTGTTCCCCGACCTGGGTGTGCGCGTGTGCGAGAA

GATGGCCCTGTACGACGTAGTCAGCAAACTCCCTATAGCCGTGATGGGAAGCTCCTACGG

ATTCCAATACTCACCAGGGCAGCGGGTTGAATTCCTCGTGCAAGCGTGGAAGTCCAAGAA

GACCCCAATGGGGTTTTCGTATGATACCCGCTGCTTTGACTCCACAGTCACTGAGAGCGA

TATCCGTACGGAGGAGGCAATCTACCAGTGTTGTGACCTGGACCCCCAAGCCCGCGTGGC

CATCAAGTCCCTCACCGAGAGGCTTTATGTCGGGGGTCCTCTTACCAATTCAA

>MT454973

CACATTTGACAGACTGCAGGTCCTGGATAGCCACTACCAGGACGTGCTCAAGGAGGTTAA

GGCGGCGGCGTCAAAAGTGAAGGCTAACTTGCTATCCGTAGAGGAAGCTTGCAGCCTGAC

GCCCCCACATTCAGCCAAATCCAAATTTGGCTATGGGGCGAAAGATGTCCGTTGCCATGC

CAGAAGGGCCGTAGACCACATCAACTCCGTGTGGAAGGACCTTCTGGAAGACAGTGTAAC

ACCAATAGATACTACCATCATGGCTAAGAACGAAGTTTTCTGCGTTCAGCCTGAGAAGGG

GGGTCGTAAGCCAGCTCGACTCATCGTGTTCCCCGACCTGGGTGTGCGCGTGTGCGAGAA

AATGGCCCTGTACGACGTGGTCAGCAAACTCCCTATAGCTGTGATGGGAAGCTCCTACGG

ATTCCAATACTCACCAGGCCAGCGGGTTGAATTCCTCGTGCAAGCGTGGAAGTCCAAGAA

GACCCCAATGGGGTTTTCGTATGATACCCGCTGCTTTGACTCCACAGTCACTGAGAGCGA

TATCCGTACGGAGGAGGCAATCTACCAGTGTTGTGACCTGGACCCCCAAGCCCGCGTGGC

CATCAAGTCCCTCACCGAGAGGCTTTATGTCGGGGGCCCTCTTACCAATTCAA

>MT454974

CACATTTGACAGACTGCAGGTCCTGGATAGCCACTACCAGGACGTGCTCAAGGAGGTTAA

GGCGGCGGCGTCAAAAGTGAAGGCTAACTTGCTATCCGTAGAGGAAGCTTGCAGCCTGAC

GCCCCCACATTCAGCCAAATCCAAATTTGGCTATGGGGCAAAAGATGTCCGTTGCCATGC

CAGAAGGGCCGTAGACCACATCAACTCCGTGTGGAAGGACCTTCTGGAAGACAGTGTAAC

ACCAATAGATACTACCATCATGGCTAAGAACGAAGTTTTCTGCGTTCAGCCTGAGAAGGG

GGGTCGTAAGCCAGCTCGACTCATCGTGTTCCCCGACCTGGGTGTACGCGTGTGCGAGAA

AATGGCCCTGTACGACGTGGTCAGCAAACTCCCTATAGCTGTGATGGGAAGCTCCTACGG

ATTCCAATACTCACCAGGCCAGCGGGTTGAATTCCTCGTGCAAGCGTGGAAGTCCAAGAA

GACCCCAATGGGGTTTTCGTATGATACCCGCTGCTTTGACTCCACAGTCACTGAGAGCGA

TATCCGTACGGAGGAGGCAATCTACCAGTGTTGTGACCTGGACCCCCAAGCCCGCGTGGC

CATCAAGTCCCTCACCGAGAGGCTTTATGTCGGGGGTCCTCTTACCAATTCAA

>MT454975

CACATTTGACAGACTGCAGGTTCTGGATAGCCACTACCAGGACGTGCTCAAGGAGGTTAA

GGCGGCGGCGTCAAAAGTGAAGGCTAACTTGCTATCCGTAGAGGAAGCTTGCAGCCTGAC

GCCCCCACATTCAGCCAAATCCAAATTTGGCTATGGGGCAAAAGATGTCCGTTGCCATGC

CAGAAGGGCCGTAGACCACATCAACTCCGTGTGGAAGGACCTTCTGGAAGACAGTGTAAC

ACCAATAGATACTACCATCATGGCTAAGAACGAAGTTTTCTGCGTTCAGCCTGAGAAGGG

GGGTCGTAAGCCAGCTCGACTCATCGTGTTCCCCGACCTGGGTGTACGCGTGTGCGAGAA

AATGGCCCTGTACGACGTGGTCAGCAAACTCCCTATAGCTGTGATGGGAAGCTCCTACGG

ATTCCAATACTCACCAGGCCAGCGGGTTGAATTCCTCGTGCAAGCGTGGAAGTCCAAGAA

GACCCCAATGGGGTTTTCGTATGATACCCGCTGCTTTGACTCCACAGTCACTGAGAGCGA

TATCCGTACGGAGGAGGCAATCTACCAGTGTTGTGACCTGGACCCCCAAGCCCGCGTGGC

CATCAAGTCCCTCACCGAGAGGCTTTATGTCGGGGGTCCTCTTACCAATTCAA

>MT454976

CACGTTTGACAGACTGCAAGTCCTGGATAGCCACTACCAGGATGTACTCAAGGAGGTTAA

GGCAGCGGCGTCAAAGGTGAAGGCTAACTTGCTATCCGTGGAGGAAGCTTGTAGCCTGAC

GCCCCCACACTCGGCCAGATCTAAGTTCGGCTATGGGGCAAAAGACGTCCGCTGCCATGC

CAGAAAGGCCGTGAACCACATCCGCTCCGTGTGGAAGGACCTTCTGGAAGACAATGTAAC

GCCTCTAGACACTACCATCATGGCCAAGAACGAGGTTTTCTGTGTTGACCCTGCGAAGGG

AGGTCGCAAGCCAGCTCGTCTCATCGTGTACCCTGATCTGAGTGTGCGCGTGTGCGAGAA

GATGGCCTTGTACGACCTGGTCAGCAAGCTCCCTCTAGCCGTGATGGGAAGCTCCTACGG

ATTCCAATACTCACCAGGACAGCGGGTTGAATTCCTCGTGCAAGCGTGGAAGTCCAAGAA

GACCCCGATGGGGTTCTCGTATGATACCCGCTGCTTTGACTCCACAGTCACTGAGAACGA

CATCCGTACGGAGGAGGCAATCTACCAATGTTGTGACCTGGACCCCCAAGCCCGTGTGGC

CATCAAGTCCCTTACTGAGAGGCTATATGTTGGGGGCCCGCTTACCAATTCAA

>MT454977

CACATTTGACAGACTGCAGGTTCTGGATAGCCACTACCAGGACGTGCTCAAGGAGGTTAA

GGCGGCGGCGTCAAAAGTGAAGGCTAACTTGCTATCCGTAGAGGAAGCTTGTAGCCTGAC

GCCCCCACATTCAGCCAGATCCAAATTTGGCTATGGGGCAAAAGACGTCCGTTGCCATGC

CAGAAGGGCCGTAGACCACATCAACTCCGTGTGGAAGGACCTTCTGGAAGACAGTGTAAC

ACCAATAGATACTACCATCATGGCTAAGAACGAAGTTTTCTGCGTTCAGCCTGAGAAGGG

GGGTCGTAAGCCAGCTCGACTCATCGTGTTCCCCGACCTGGGTGTACGCGTGTGCGAGAA

AATGGCCCTGTACGACGTGGTCAGCAAACTCCCTATAGCTGTGATGGGAAGCTCCTACGG

ATTCCAATACTCACCAGGCCAGCGGGTTGAATTCCTCGTGCAAGCGTGGAAGTCCAAGAA

GACCCCAATGGGGTTTTCGTATGATACCCGCTGCTTTGACTCCACAGTCACTGAGAGCGA

TATCCGTACGGAGGAGGCAATCTACCAGTGTTGTGACCTGGACCCCCAAGCCCGCGTGGC

CATCAAGTCCCTCACCGAGAGGCTTTATGTCGGGGGTCCTCTTACCAATTCAA

>MT347782

GCTTGCTCCGACGGTTGCACCAGTGGATCAATGAAGACTACCC

AAGTCCTTGCAGCGGTGATTGGCTGCGTACCATCTGGGATTGGGTTTGCATGGTGCTGTC

CGACTTCAAGACGTGGCTCTCCGCTAAGATCATGCCAACGCTTCCTGGCCTGCCCTTCAT

TTCCTGTCAGAAGGGATACAAAGGCGTGTGGCGAGGGGATGGTGTGATGTCAACACGCTG

CACTTGCGGAGCAACACTAGCCGGTCATGTGAAGAATGGGTCCATGCGGCTTGCAGGGCC

GCGTACATGTGCCAACATGTGGTATGGTACTTTTCCCATCAATGAGCACACCACCGGA

>MT347783

CTGCTCCGGCGGTTGCACCAGTGGATCAATGAAGACTACCC

GAGTCCTTGCAGCGGTGACTGGCTGCGTGACATCTGGGACTGGGTTTGCTCAGTGTTGTC

TGACTTCAGGACATGGCTCTCTGCTAAGATTATGCCAACACTCCCCGGGCTGCCCTTCAT

TTCCTGTCAAAAGGGATATAAGGGCGTGTGGCGGGGGGACGGTGTGATGTCGACACGCTG

TCCTTGCGGGGCATCAATAACTGGTCATGTGAAAAATGGGTCCATGCGGCTTGCAGGGCC

GCGTACATGTGCTAACATGTGGCACGGTACTTTCCCCATCAATGAGCACACCACCGG

>MT347784

GGTTACACCAGTGGATCAATGAAGACTACCC

AAGCCCCTGCAGCGGCGATTGGCTGCGTGACATCTGGGACTGGGTTTGCACGGTGTTGTC

CGACTTCAAAACATGGCTCTCCGCCAAAATTATGCCAACGCTTCCTGGACTGCCCTTCAT

CTCCTGTCAAAAGGGGTACAAGGGCGTGTGGCGGGGGGACGGCGTGATGTCAACACGCTG

TCCTTGCGGGGCATCAATAACCGGTCATGTGAAGAATGGGTCCATGCGGCTTGCAGGGCC

GCGTACATGTGCTAACATGTGGCACGGTACTTTTCCCATCAATGAGCACACCACCGGA

>MT347785

GTGGATCAATGAAGACTACCCAAGTCCTTGCAGCGGTAATTGGCTGCGCGACATCTGGGACTGGGTTTGCACGGAGTTGCATGACTTCAGGGCATGGCTCTCCGCTAAGATTATGCCAGCGCTCCCCGGGCTGCCCTTCATTTCCTGTCAAAAGGGATATAAGGGCGTGTGGCGGGGAGACGGTGTGATGTCAACACGCTGTCCTTGCGGAGCATCAATATCCGACCATGTGAAGAATGGGTCCATGCGGCTTGCAGGGCCGCGTACATGTGCCAACCTGTGGCATGGTACTTTCCCCATCAACGAGCACACCACCGGA

>MT347786

CATGTCTGCTCCGGCGATTACACCAGTGGATCAATGAAGACTACCC

AAGTCCTTGCAGCGGCGATTGGCTGCGTGACATCTGGGACTGGGTTTGCTCGGTGTTGTC

TGACTTCAAGACATGGCTCTCTGCTAAGATTATGCCGGCGTTCCCTGGGCTGCCCTTCAT

TTCCTGTCAAAAGGGATATAAGGGCGTGTGGCGGGGGGACGGTGTGATGTCGACACGCTG

TCCTTGCGGGGCATTAATAACTGGTCATGTAAAGAATGGTTCCATGCGGATTGCAGGGCC

GCGTACATGTGCTAACATGTGGTATGGTACTTTCCCCATCAATGAGTACACCACCGG

>MT347787

GGCGGTTGCACCAGTGGATTAATGAAGACTACCC

AAGTCCTTGCAGCGGCGACTGGCTGCGTGACATCTGGGACTGGGTTTGCTCAGTGTTGTC

CGACTTCAGGACATGGCTCTCTGCTAAGATTATGCCAGCGCTCCCTGGGCTGCCCTTCAT

TTCCTGTCAAAAGGGATATAAGGGCGTGTGGCGGGGGGACGGTGTGATGTCGACACGCTG

TACTTGCGGGGCACCAATAACTGGCCATGTGAAGAATGGGTCTATGCGGCTTGCAGGGCC

GCGTACATGTGCTAACATGTGGTACGGTACCTTCCCCATCAATGAGTACACCACCGG

>MT347788

TGCACCAGTGGATCAATGAAGACTACCC

AAGTCCTTGCAGCGGCGATTGGCTGCGTACCATCTGGGACTGGGTTTGCACGGTGTTGTC

TGACTTCAAGACATGGCTCTCTGCTAAGATCATGCCAGCGCTCCCCGGACTGCCTTTCAT

TTCCTGTCAAAAGGGATACAAGGGCGTGTGGCGGGGGGATGGCGTAATGTCAACACGCTG

TCCTTGCGGGGCAACAATAGCCGGTCATGTGAAGAATGGGTCTATGCGGCTTGCAGGGCC

GCGTACATGTGCTAACATGTGGCACGGTACTTTCCCCATCAACGAGTACACCACCGGAC

>MT347789

ACTCCGGCGGCTGCACCAGTGGATCAATGAAGACTACCC

GAGTCCCTGCAGCGGCGACTGGCTGCGTGACATCTGGGACTGGGTTTGCACAGTGTTGTC

CGACTTCAGGAGATGGCTCTCTGCTAAGATCATGCCAACACTCCCCGGGCTGCCCTTCAT

TTCCTGTCAAAAGGGATACAAGGGCGTGTGGCGGGGGGACGGTGTGATGTCGACACGCTG

TCCTTGCGGGGCACTAATGACTGGCCATGTAAAGAATGGGTCTATGCGGCTTGCAGGGCC

GCGTACATGTGCCAACATGTGGTACGGTACCTTCCCCATTAATGAGTACACCACCGG

>MT347790

GGCGGCTGCACAGATGGATCAATGAAGATTACCC

AAGTCCCTGCAGCGGTGACTGGCTGCGTGACATCTGGGACTGGGTTTGCACGATGTTGTC

CGACTTCAAGACATGGCTCTCTGCTAAGATCATGCCAGCGCTCCCCGGGCTGCCCTTCAT

TTCCTGTCAAAAGGGATACAAGGGCGTGTGGCGGGGGGACGGTGTGATGTCAACACGCTG

TCCTTGCGGGGCACTAATAACTGGCCATGTAAAGAATGGGTCTATGCGGCTTGCAGGGCC

GCGTACATGTGCTAACATGTGGTACGGTACCTTCCCCATTAATGAGTACACCACCGG

>MT347791

GGCGGCTGCACAGATGGATCAATGAAGATTACCC

AAGTCCCTGCAACGGTGACTGGCTGCGTGACATCTGGGACTGGGTTTGCTCGGTGTTGTC

CGACTTCAAGACATGGCTCTCTGCTAAGATCATGCCAGCGCTCCCCGGGCTGCCCTTCAT

TTCCTGTCAAAAGGGATACAAGGGCGTGTGGCGGGGGGACGGTGTGATGTCAACACGCTG

TCCTTGCGGGGCATCAATAACTGGCCATGTAAAGAATGGGTCTATGCGGCTTGCAGGGCC

GCGTACATGTGCTAACATGTGGTACGGTACCTTCCCCATCAATGAGTACACCACCGG

>MT347792

TGCTTCGGCGGCTGCACAGATGGATCAATGAAGATTACCC

AAGTCCCTGCAGCGGTGACTGGCTGCGTGACATCTGGGACTGGGTTTGCACGATGTTGTC

CGACTTCAAGGCATGGCTCTCTGCTAAGATCATGCCAGCGCTCCCCGGGCTGCCCTTCAT

TTCCTGTCAAAAGGGATACAAGGGCGTGTGGCGGGGGGACGGTGTGATGTCAACACGCTG

TCCTTGCGGGGCACTAATAACTGGCCATGTAAAGAATGGGTCTATGCGGCTTGCAGGGCC

GCGTACATGTGCTAACATGTGGTACGGTACCTTCCCCATTAATGAGTACACCACCGGA

CCCAGCA

>MT347793

TGCTTCGGCGGCTGCACAGATGGATCAATGAAGATTACCC

AAGTCCCTGCAGCGGTGACTGGCTGCGTGACATCTGGGACTGGGTTTGCTCGGTGTTGTC

CGACTTCAAGACATGGCTCTCTGCTAAGATCATGCCAGCGCTCCCCGGGCTGCCCTTCAT

TTCCTGTCAAAAGGGATACAAGGGCGTGTGGCGGGGGGACGGTGTGATGTCAACACGCTG

TCCTTGCGGGGCACTAATAACTGGCCATGTAAAGAATGGGTCTATGCGGCTTGCAGGGCC

GCGTACATGTGCTAACATGTGGTACGGTACCTTCCCCATTAATGAGTACACCACCGGACCCGG

>MT347794

GGCGGCTGCACAGATGGATCAATGAAGATTACCC

AAGTCCCTGCAGCGGTGACTGGCTGCGTGCCATCTGGGACTGGGTTTGCACGGTGTTGTC

CGACTTCAAGACATGGCTCTCTGCTAAGATCATGCCAGCGCTCCCCGGGCTGCCCTTCAT

TTCCTGTCAAAAGGGATACAAGGGCGTGTGGCGGGGGGACGGTGTGATGTCGACACGCTG

TCCTTGCGGGGCACTAATAACTGGCCATGTAAAGAATGGGTCTATGCGGCTTGCAGGGCC

GCGTACATGTGCTAACATGTGGTACGGTACCTTCCCCATTAATGAGTACACCACCGG

>MT347795

GGCGGCTGCACAGATGGATCAATGAAGATTACCC

AAGTCCCTGCAGCGGTGACTGGCTGCGTGACATCTGGGACTGGGTTTGCACGATGTTGTC

CGACTTCAAGACATGGCTCTCTGCTAAGATCATGCCAGCGCTCCCCGGGCTGCCCTTCAT

TTCCTGTCAAAAGGGATACAAGGGCGTGTGGCGGGGGGACGGTGTAATGTCAACACGCTG

TCCTTGCGGGGCACTAATAGCTGGCCATGTAAAGAATGGGTCTATGCGGCTTGCAGGGCC

GCGTACATGTGCTAACATGTGGTACGGTACCTTCCCCATCAATGAGTACAC

>MT347796

GGCGGCTGCACAGATGGATCAATGAAGATTACCC

AAGTCCCTGCAGCGGTGACTGGCTGCGTGACATCTGGGACTGGGTTTGCACGATGTTGTC

CGACTTCAAGACATGGCTCTCTGCTAAGATCATGCCAGCGCTCCCCGGGCTGCCCTTCAT

TTCCTGTCAAAAGGGATACAAGGGCGTGTGGCGGGGGGACGGTGTGATGTCGACACGCTG

TCCTTGCGGGGCACTAATAACTGGCCATGTAAAGAATGGGTCTATGCGGCTTGCAGGGCC

GCGTAGATGTGCTAACATGTGGCACGGTACCTTCCCCATTAATGA

>MT347797

GGCGGCTGCACAGATGGATCAATGAAGACTATCC

AAGTCCCTGCAGCGGTGACTGGCTGCGTGACATCTGGGACTGGGTTTGCACGATGTTGTC

CGACTTCAAGACATGGCTCTCTGCTAAGATCATGCCAGCGCTCCCCGGGCTGCCCTTCAT

TTCCTGTCAAAAGGGATACAAGGGCGTGTGGCGGGGGGACGGTGTGATGTCAACACGCTG

TCCTTGCGGGGCACAAATATCTGGCCATGTAAAGAATGGGTCTATGCGGCTTGCAGGGCC

GCGTACATGTGCTAACATGTGGTACGGTACCTTCCCCATTAATG

>MT347798

GGCGGCTGCACAGATGGATCAATGAAGATTACCC

AAGTCCCTGCAGCGGTGACTGGCTGCGTGACATCTGGGACTGGGTTTGCACGGTGTTGTC

CGACTTCAAGACATGGCTCTCTGCTAAGATCATGCCAGCGCTCCCCGGGCTGCCCTTCAT

TTCCTGTCAAAAGGGATACAAGGGCGTGTGGCGGGGGGACGGTGTGATGTCAACACGCTG

TCCTTGCGGGGCACTAATAACCGGTCATGTAAAGAATGGGTCTATGCGGCTTGCAGGGCC

GCGTACATGTGCTAACATGTGGTACGGTACCTTCCCCATCAATGAGTAC

>MT347799

GGCGGCTGCACAGATGGATCAATGAAGATTACCC

AAGTCCCTGCAGCGGTGACTGGCTGCGTGACATCTGGGACTGGGTTTGCACGATGTTGTC

CGACTTCAAGACATGGCTCTCTGCTAAGATCATGCCAGCGCTCCCCGGGCTGCCCTTCAT

TTCCTGTCAAAAGGGATACAAGGGCGTGTGGCGGGGGGACGGTGTGATGTCAACACGCTG

TCCTTGCGGGGCATCAATAACCGGCCATGTGAAGAATGGGTCCATGCGGCTTGCAGGGCC

GCGTACATGTGCTAACATGTGGTACGGTACCTTCCCCATTAATGAGTACAC

>MT347800

GCGGCTGCACAGATGGATCAATGAAGATTACCC

AAGTCCCTGCAGCGGTGACTGGCTGCGTGACATCTGGGACTGGGTTTGCACGGTGTTGTC

CGACTTCAAGACATGGCTCTCTGCTAAGATCATGCCAGCGCTCCCCGGACTGCCCTTCAT

TTCCTGTCAAAAGGGATACAAGGGCGTGTGGCGGGGGGACGGTGTGATGTCAACACGCTG

TCCTTGCGGGGCACTAATAACTGGCCATGTAAAGAATGGGTCTATGCGGCTTGCAGGGCC

GCGTACATGTGCTAACATGTGGTACGGTACCTTCCCCATCAATGAGTACACC

>MT347801

CGGCTGCACAGATGGATCAATGAAGATTACCC

AAGTCCCTGCAGCGGTGACTGGCTGCGTGACATCTGGGACTGGGTTTGCTCGGTGTTGTC

CGACTTCAAGACATGGCTCTCTGCTAAGATCATGCCAGCGCTCCCCGGGCTGCCCTTCAT

TTCCTGTCAAAAGGGATACAAGGGCGTGTGGCGGGGGGACGGTGTGATGTCAACACGCTG

TCCTTGCGGGGCACTAATAACTGGCCATGTAAAGAATGGGTCTATGCGGCTTGCAGGGCC

GCGTACATGTGCTAACATGTGGTACGGTACCTTCCCCATTAATGAGTACACCA

>MT347802

GCGGCTGCACAGATGGATCAATGAAGATTACCC

AAGTCCCTGCAGCGGTGACTGGCTGCGTGACATCTGGGACTGGGTTTGCACGATGTTGTC

CGACTTCAAGACATGGCTCTCTGCTAAGATCATGCCAGCGCTCCCTGGGCTGCCCTTCAT

TTCCTGTCAAAAGGGATACAAGGGCGTGTGGCGGGGGGACGGTGTGATGTCAACACGCTG

TCCTTGCGGGGCATTGATAACTGGCCATGTAAAGAATGGGTCTATGCGGCTTGCAGGGCC

GCGTACATGTGCTAACATGTGGTACGGTACCTTCCCCATCAATGAGTACACCAC

>MT347803

CGGCTGCACAGATGGATCAATGAAGATTACCC

AAGTCCCTGCAGCGGTGACTGGCTGCGTGACATCTGGGACTGGGTTTGCACGATGTTGTC

CGACTTCAAGACATGGCTCTCTGCTAAGATCATGCCAGCACTCCCTGGGCTGCCCTTCAT

TTCCTGTCAAAAGGGATACAAGGGCGTGTGGCGGGGGGACGGTGTGATGTCAACACGCTG

TCCTTGCGGGGCACTAATAACTGGCCATGTAAAGAATGGGTCCATGCGGCTTGCAGGGCC

GCGTACATGTGCTAACATGTGGTACGGTACCTTCCCCATTAATGAGTACACCAC

>MT502204

ACGGAACGGCTTTACTGCGGGGGCCCTATGTTCAACAGCAAAGGAGCCCAGTGTGGTTAT

CGCCGTTGCCGTGCTAGTGGAGTTCTGCCTACCAGCTTCGGCAACACAATCACCTGTTAC

ATCAAGGCTACGGCGGCCGCTAGAGCTGCAGGCCTCCGGAACCCAGACTTCCTTGTCTGC

GGAGATGATCTAGTCGTGGTGGCTGAGAGTGATGGCGTCGACGAGGATAGAGCAGCCCTG

AGAGCCTTCACGGAGGCTATGACCAGGTATTCTGCTCCACCCGGAGATGCCCCACAGCCC

ACCTACGACCTTGAGCTCATTACATCTTGCTCCTCTAACGTCTCCGTGGCACGGGACGAC

AAGGGGAAGAGGTACTATTACCTCACCCGCGATGCCACTACTCCCCTGGCCCGTGCGGCC

TGGGAAACAGCTCGTCACACTCCAGTTAACTCCTGGTTGGGGAACATCATCATGTACGCG

CCTACTATCTGGGTGCGCATGGTGATGATGACACACTTTTTCTCCATACTCCAGTCTCAG

GAGATACTTGATCGCCCCCTTGACTTTGA

>MT502205

ACGGAGCGGCTTTACTGCGGGGGCCCTATGTTTAACAGCAAGGGGGCCCAGTGTGGTTAT

CGCCGTTGCCGTGCCAGTGGAGTTCTGCCTACCAGCTTTGGCAACACAATCACTTGTTAC

ATCAAGGCTACAGCGGCTGCGAAGGCCGCAGGTCTCCGGGACCCGGACTTTCTCGTCTGC

GGAGATGATTTGGTCGTGGTGGCCGAGAGTGATGGCGTCGACGAGGATGGAACAGCCCTG

AGAGCCTTCACGGAGGCTATGACCAGGTATTCTGCTCCACCCGGAGATGCTCCACAGGCC

ACCTACGACCTTGAGCTCATTACATCTTGCTCCTCCAACGTCTCTGTGGCACTGGACAAT

AAGGGAAAGAGGTATTATTACCTCACTCGTGATGCCACTACTCCCCTGACCCGTGCGGCT

TGGGAGACAGCTCGTCACACTCCAGTCAACTCCTGGTTAGGCAACATCATCATGTACGCG

CCTACTATTTGGGTGCGCATGGTAATGATGACACATTTTTTCTCCATACTCCAATCCCAG

GAGATACTTGACCGACCCCTTGACTTTGA

>MT502206

ACGGAGCGGCTTTACTGCGGGGGCCCTATGTTCAACAGCAAAGGGGCCCAGTGCGGTTAT

CGCCGTTGTCGTGCCAGTGGAGTTTTGCCTACCAGCTTCGGCAACACAATCACCTGTTAC

ATCAAGGCCACAGCGGCCGCGAAGGCCGCGGGTCTTCGGAACCCAGACTTTCTTGTCTGC

GGAGACGATTTGGTCGTGGTGGCTGAGAGTGATGGCGTCGATGAGGACAGGGCAGCCCTG

AGAGCCTTCACAGAGGCTATGACCAGGTATTCTGCTCCACCCGGAGATGCTCCACAGGCC

ACCTATGACCTTGAGCTCATCACATCTTGCTCCTCCAACGTCTCTGTGGCACGGGACGAC

AAGGGCAAGAGGTACTATTACCTCACCCGTGATGCCACTACTCCCCTAGTCCGTGCAGCT

TGGGAGACAGCTCGTCACACTCCAGTCAACTCCTGGTTAGGCAACATCATCATGTACGCG

CCCACCATCTGGGTACGCATGGTACTGATGACACATTTTTTCTCCATACTCCAGTCCCAG

GAAATACTTGATCGGTCCCTTGACTTTGAAA

>MT502207

ACGGAACGGCTTTACTGCGGGGGCCCTATGTTCAACAGCAAGGGGGCCCAGTGCGGTTAT

CGCCGTTGCCGTGCCAGTGGAGTTCTGCCTACCAGCTTCGGCAACACAATCACTTGTTAC

ATCAAGGCCACAGCGGCTGCCAGAGCCGCAGGCCTCCGGAACCCGGACTTTCTTGTCTGC

GGAGATGATCTGGTCGTGGTGGCTGAGAGTGACGGCGTCGACGAGGATAGAGCAGCCCTG

AGAGCCTTCACGGAGGCTATGACCAGGTATTCTGCTCCACCCGGAGATGCTCCACAGCCC

ATCTACGACCTTGAGCTCATTACATCTTGCTCCTCCAACGTCTCCGTGGCACTGGACGAC

AAGGGGAAGAGGTATTATTACCTCACCCGTGATGCCACTACTCCCCTAGCCCGTGCGGCT

TGGGAAACAGCTCGACACACTCCAGTCAACTCCTGGTTAGGCAACATCATCATGTACGCG

CCTACCATCTGGGTACGCATGGTACTGATGACACACTTCTTCTCCATACTCCAATCCCAG

GAGATACTTGACCGACCCCTTGACTTTGAAA

>MT502208

ACGGAGCGGCTTTACTGCGGGGGTCCTATGTTCAACAGCAAGGGGGCCCAGTGCGGTTAT

CGCCGTTGTCGTGCCAGTGGAGTTCTGCCTACCAGCTTCGGCAACACAATCACTTGTTAC

ATCAAGGCCACAGCGGCTGCAAGAGCCGCAGGCCTCCGGAACCCGGACTTTCTTGTCTGC

GGAGATGATTTGGTCGTGGTGGCTGAGAGTGACGGCGTCGACGAGGATAGAGCAGCCCTG

AGAGCCTTCACGGAGGCTATGACCAGGTATTCTGCTCCACCCGGAGATGCTCCACAGCCC

ATATACGACCTTGAGCTCATTACATCTTGCTCCTCCAACGTCTCCGTGGCACTAGACGAC

AAGGGGAAGAGGTATTATTACCTCACCCGTGATGCCACTACTCCCCTGGCCCGTGCGGCT

TGGGAAACAGCTCGTCACACTCCAGTCAACTCCTGGTTAGGCAACATCATCATGTACGCG

CCTACCATCTGGGTGCGCATGGTGATGATGACACACTTTTTCTCCATACTCCAATCCCAG

GAGATACTTGACCGACCCCTTGACTTTGAAA

>MT502209

ACGGAACGGCTTTACTGCGGGGGCCCCATGTTTAACAGCAAAGGGGCCCAGTGTGGTTAT

CGCCGCTGCCGTGCCAGTGGAGTCCTGCCTACCAGCTTTGGTAATACAATCACTTGTTAC

ATCAAGGCTACGGCGGCCGCTAGGGCCGCAGGCCTCCGGAACCCGGACTTTCTTGTCTGC

GGGGATGATTTGGTCGTGGTGGCTGAGAGTGACGGCGTCGAGGAAGACAGAGCAGCCCTG

AGAGCCTTCACGGAGGCTATGACCAGGTACTCTGCTCCACCCGGAGATGCCCCGCAGCCT

ACCTACGACCTCGAGCTTATTACATCTTGCTCCTCCAACGTCTCCGTGGCACTGGACGAC

AAGGGTAAGAGGTACTATTACCTCACCCGTGATGCCACCACTCCCCTGGCCCGTGCGGCT

TGGGAGACAGCTCGTCACACTCCAGTTAACTCCTGGCTGGGCAACATCATCATGTACGCG

CCTACCATCTGGGTGCGCATGGTAATGATGACACACTTTTTCTCCATACTCCAGTCCCAG

GAGATACTTGATCGCCCTCTTGACTTTGA

>MT502210

ACGGAACGGCTTTACTGCGGGGGCCCTATGTTCAACAGCAAGGGGGCCCAGTGTGGTTAT

CGCCGTTGCCGCGCCAGTGGAGTTCTGCCCACCAGCTTTGGTAACACAATAACTTGTTAC

ATCAAGGCCACAGCGGCTGCGAGAGCTGCGGGCCTTCGAAACCCGGACTTTCTTGTCTGC

GGAGATGATTTGGTCGTGGTGGCAGAGAGTGATGGCGTCAACGAGGATGGAGCAGCCCTG

AGAGCTTTCACGGAGGCTATGACCAGGTATTCTGCTCCGCCCGGAGATGCTCCACAGCCC

ACCTACGACCTCGAGCTCATTACATCTTGCTCCTCCAACGTCTCTGTAGCGCGGGACTAC

AAGGGGAAGAGGTACTATTACCTCACCCGTGATCCCACTACTCCCCTGGCCCGTGCGGCT

TGGGGAACAGCTCGTCACACTCCAGTCAACTCCTGGTTGGGCAACATCATCATGTACGCG

CCTACCATCTGGGTGCGCATGGTAATGATGACACACTTCTTCTCCATACTCCAATCCCAG

GAGATACTTGATCGGTCCCTTGACTTTGA

>MT502211

ACGGAGCGGCTTTACTGCGGGGGTCCCATGTTCAACAGCAAGGGGGCCCAGTGCGGTTAT

CGCCGTTGCCGTGCCAGTGGAGTCCTGCCTACCAGCTTTGGCAACACAATCACTTGTTAC

ATCAAGGCCACAGCGGCTGCGAAAGCTGCAGGCCTCCAGAACCCGGACTTTCTTGTCTGC

GGAGATGATCTGGTCGTGGTAGCTGAGAGTGGTGGCGTCGATGAGGATAGAGCAGCCCTG

AGAGCCTTCACGGAGGCTATGACCAGGTATTCTGCTCCACCCGGAGATGCCCCACAACCC

GCCTACGACCTGGAGCTCATTACATCTTGCTCCTCCAACGTCTCCGTAGCACGGGACGAC

AAGGGGAAGAGGTACTATTACCTCACCCGTGATGCCACCACTCCCCTAGCCCGTGCGGCT

TGGGAAACAGCTCGTCACACTCCAGTTAACTCCTGGTTAGGCAACATCATCATGTACGCG

CCCACTATCTGGGTGCGCATGGTAATGATGACACATTTTTTCTCCATACTCCAATCCCAG

GAGATACTAGATCGACCCCTTGACTTTGAAA

>MT502212

ACGGAGCGGCTTTACTGCGGGGGCCCTATGTTCAACAGCAAAGGGGCCCAGTGCGGTTAT

CGCCGTTGCCGCGCAAGCGGAGTTCTGCCTACCAGCTTCGGCAACACAATCACTTGCTAC

ATCAAGGCCACAGCGGCTGCAAAGGCCGCAGGCCTCCGGAACCCGGACTTTCTTGTCTGC

GGAGATGATTTGGTCGTGGTAGCTGAGAGCGATGGCGTCGATGAGGATAGAGCAGCCCTG

CGAGCCTTCACGGAGGCTATGACCAGGTACTCTGCTCCACCTGGCGATGTTCCACAGCCC

ACCTACGACCTTGAGCTCATTACATCCTGCTCTTCTAACGTCTCCGTGGCACTAGACAAC

AAGGGGAAGAGGTATTATTACCTCACCCGTGATGCCACTACTCCCCTAGCCCGTGCGGCT

TGGGAAACAGCTCGTCACACTCCAGTTAACTCCTGGTTAGGCAACATCATCATGTACGCG

CCTACCATCTGGGTGCGCATGGTAATGATGACGCACTTTTTCTCCATACTCCAATCCCAG

GAAATACTTGATCGACCCCTTGACTTTGAAA

>MT502213

ACGGAGCGGCTTTACTGCGGAGGTCCTATGTTCAACAGCAAGGGGGCCCAGTGTGGTTAT

CGCCGTTGCCGTGCCAGTGGAGTTCTGCCCACCAGCTTTGGCAACACAATCACTTGTTAC

ATCAAGGCCACAGCGGCCGCGAAGGCCGCAGGCCTCCGGGACCCGGACTTTCTTGTCTGC

GGAGATGATCTGGTCGTGGTGGCTGAGAGTGATGGTGTCGATGAGGATAGAGCAGCCCTG

AGAGCCTTCACGGAGGCTATGACCAGGTATTCTGCTCCACCCGGAGACGCTCCACAGCCC

ACCTACGACCTTGAGCTCATTACATCTTGCTCCTCCAACGTCTCTGTGGCACAGGACAAC

AAGGGGAAGAGGTATTATTACCTCACCCGTGATGCCACCACTCCCCTAGCCCGCGCGGCT

TGGGAAACAGCTCGTCACACTCCAGTAAACTCCTGGTTGGGCAACATCATCATGTACGCG

CCTACCATCTGGGTGCGCATGGTAATGATGACACACTTTTTCTCCATACTCCAATCCCAG

GAGATACTTGACCGACCCCTTGACTTTGA

>MT502214

ACGGAACGGCTTTACTGCGGAGGCCCTATGTTCAACAGCAAAGGAGCCCAGTGCGGTTAT

CGCCGTTGCCGTGCCAGTGGAGTTCTGCCTACCAGCTTTGGCAACACAATCACCTGTTAC

ATCAAGGCTACGGCAGCTGCTAGAGCTGCAGGCCTCCGGAACCCGGACTTTCTTGTCTGC

GGGGACGATCTAGTCGTGGTGGCCGAGAGTGACGGCGTCGACGAGGATAGAGCAGCCCTG

AGAGCCTTCACGGAGGCTATGACCAGGTACTCTGCTCCACCCGGAGATGCTCCACAGCCC

ACCTACGACCTTGAGCTCATCACATCTTGCTCCTCTAACGTCTCCGTAGCACGGGACGAC

AAGGGGAAGAGGTACTATTACCTCACCCGTGATGCCACTACTCCCTTGGCCCGTGCGGCT

TGGGAAACAGCTCGCCACACTCCAGTCAACTCCTGGTTGGGCAACATTATCATGTACGCG

CCTACCATCTGGGTGCGCATGGTAATGATGACACATTTTTTCTCCATACTCCAGTCCCAG

GAGGTACTTGATCGCCCCCTTGACTTTGA

>MT502215

ACGGAACGACTTTACTGCGGGGGCCCCATGTTCAACAGCAAGGGGGCCCAGTGTGGTTAT

CGCCGTTGCCGTGCCAGTGGAGTTTTGCCTACCAGCTTTGGCAACACAATCACTTGCTAC

ATCAAAGCCACAGCGGCTGCGAAAGCTGCAGGCCTCCGGAACCCGGACTTTCTTGTCTGC

GGGGATGATTTGGTCGTGGTGGCTGAGAGTGATGGAGTTGATGAGGACAGAGCAGCCCTG

AGAGCCTTCACGGAGGCTATGACCAGGTACTCTGCTCCACCTGGAGATGCTCCACAGCCC

ACCTATGACCTTGAGCTCATTACATCTTGCTCCTCCAACGTCTCCGTGGCATTGGACAAC

AAGGGGAGAAGGTACTACTACCTCACCCGTGATGCCACTACTCCCCTAGCCCGTGCGGCT

TGGGAAACAGCTCGACACACTCCAGTTAACTCCTGGTTAGGCAACATCATTATGTATGCA

CCTACCATCTGGGTGCGCATGGTGATGATGACACACTTTTTCTCCATACTCCAATCCCAA

GAGATACTTGATCGACCCCTTGACTTTGAAA

>MT502217

ACGGAACGGCTTTACTGCGGAGGCCCTATGTTCAACAGCAAGGGGGCCCAGTGCGGTTAT

CGCCGTTGCCGTGCCAGTGGAGTTCTGCCTACCAGCTTCGGCAACACAATCACTTGTTAC

ATCAAGGCTACGGCGGCTGCTAGAGCCGCAGGCCTCCGGAACCCGGACTTTCTTGTCTGC

GGGGACGATCTAGTCGTGGTAGCTGAGAGTGGTGGCGTCGACGAGGACAGAACAGCCCTG

AGAGCCTTCACGGAGGCTATGACCAGGTATTCTGCTCCACCCGGAGATGCTCCACAGCCT

ACCTACGACCTTGAGCTTATTACATCTTGCTCCTCCAACGTCTCCGTGGCACTGGACGGC

AAGGGGAAGAGGTACTATTACCTCACCCGTGATGCCACTACTCCCCTGGCCCGTGCGGCT

TGGGAAACAGCTCGTCACACTCCAGTTAACTCCTGGTTGGGCAACATTATCATGTACGCG

CCCACCATTTGGGTGCGCATGGTAATGATGACACACTTTTTCTCCATACTCCAGTCCCAG

GAGGTACTTGATCGCCCCCTTGACTTTGA

>MT502218

ACGGAGCGGCTCTACTGCGGGGGCCCTATGTTTAACAGCAAGGGGACCCAGTGTGGTTAC

CGCCGCTGCCGTGCTAGTGGAGTTTTGCCTACCAGCTTCGGCAATACAATCACTTGTTAC

ATCAAGGCCACAGCGGCTGCGAGGGCCGCAGGCCTCCGGAACCCGGACTTTCTCGTCTGC

GGAGATGATTTGGTCGTGGTGGCTGAGAGTGATGGCGTCGACGAGGATGGGGCAGCCCTG

AGAGCCTTCACGGAGGCTATGACCAGGTATTCTGCTCCGCCCGGAGATGCTCCACAACCC

ACCTACGACCTCGAGCTCATTACATCTTGCTCCTCTAACGTCTCTGTAGCGCAGGACAAC

AAGGGAAAGAGGTATTATTACCTCACCCGTGATGCCACTACCCCCCTGGCCCGTGCGGCT

TGGGAGACAGCTCGTCACACTCCAGTCAACTCCTGGTTGGGCAACATCATCATGTACGCG

CCCACCATCTGGGTGCGCATGGTAATGATGACACACTTTTTCTCCATACTCCAATCCCAG

GAGATACTTGACCGACCCCTTGACTTTGA

>MT502219

ACGGAGCGGCTTTACTGTGGGGGCCCTATGTTCAACAGCAAGGGGGCCCAGTGTGGTTAT

CGCCGCTGCCGTGCTAGTGGAGTCTTGCCTACCAGCTTCGGCAATACAATCACTTGTTAC

ATCAAGGCTACAGCGGCTGCAAGGGCCGCAGGCCTCCGGAACCCGGACTTTCTCGTCTGC

GGAGATGATCTAGTCGTGGTGGCTGAGAGTGATGGCGTCGACGAGGATAGGGCAGCCCTG

AGAGCCTTCACGGAGGCTATGACCAGGTATTCTGCTCCACCCGGAGATGCTCCACAGCCT

ACCTACGACCTTGAGCTCATTACATCTTGCTCCTCTAACGTCTCCGTGGCACTGGACAAC

AAGGGAAAGAGGTATTATTACCTCACCCGTGATGCCACCACTCCCCTGGCCCGTGCGGCT

TGGGAGACAGCTCGACACACTCCAGTTAACTCCTGGTTGGGCAACATCATCATGTACGCG

CCCACCATCTGGGTGCGCATGGTGATGATGACACATTTTTTCTCCATACTCCAATCCCAG

GAGATACTTGATCGGCCCCTCGACTTTGA

>MT502220

ACGGAGCGGCTTTACTGCGGGGGCCCTATGTTCAACAGCAAGGGGGCCCAGTGTGGTTAT

CGCCGTTGCCGTGCCAGTGGAGTTCTACCTACCAGCTTCGGCAATACAATCACTTGTTAC

ATCAAGGCCACAGCGGCTGCGAAAGCTGCAGGCCTCCGGAACCCGGACTTTCTTGTCTGC

GGAGATGATCTGGTCGTGGTGGCTGAGAGCGATGGCGTCGACGAGGATAGAGCATCCCTG

AGAGCCTTCACGGAGGCTATGACCAGGTACTCTGCTCCACCCGGAGATGCTCCGCAGCCC

ACCTACGATCTTGAGCTTATTACATCCTGCTCCTCTAACGTCTCCGTGGCACGGGACAAC

AAGGGGAAGAGGTACTACTACCTCACCCGTGATGCCACTACTCCCCTGGCCCGTGCGGCT

TGGGAAACAGCTCGTCACACTCCAGTTAACTCCTGGTTGGGCAACATCATCATGTACGCG

CCTACCATATGGGTGCGCATGGTAATGATGACGCACTTTTTCTCCATACTCCAATCCCAG

GAGATACTTGACCGACCCCTTGACTTTGAAA

>MT502221

ACGGAGCGGCTTTACTGCGGGGGCCCTATGTTTAACAGCAAAGGGGCCCAGTGTGGTTAT

CGCCGTTGCCGTGCCAGTGGAGTTCTGCCTACCAGCTTCGGCAATACTATCACTTGTTAC

ATCAAGGCCACAGCGGCTGCGAGGGCCGCAGGCCTCCGGAACCCGGACTTTCTTGTCTGC

GGAGATGATCTAGTAGTAGTGGCTGAGAGTGATGGCGTCGACGAGGATAGAGCAGCCCTG

AGAGCCTTCACGGAGGCTATGACCAGGTATTCTGCTCCACCCGGAGATGCCCCACAGCCT

ACCTACGACCTTGAGCTCATCACATCTTGCTCCTCTAACGTCTCCGTAGCGCGGGACAAC

AAGGGGAAGAGGTACTATTACCTCACCCGTGATGCCACTACTCCCCTGGCCCGCGCAGCT

TGGGAAACAGCTCGTCACACTCCAGTTAACTCCTGGTTGGGCAACATCATCATGTACGCG

CCTACCATCTGGGTACGCATGGTAATGATGACACACTTTTTCTCCATACTCCAATCCCAG

GAGATACTTGATCGCCCCCTTGACTTTGA

>MT502223

ACGGAACGGCTTTACTGCGGGGGCCCCATGTTCAACAGCAAGGGGGCCCAGTGTGGTTAT

CGCCGTTGCCGTGCCAGTGGAGTTCTGCCTACCAGCTTCGGCAACACAATGACTTGTTAC

ATCAAGGCCACGGCGGCTGCGAAGGCCGCAGGCCTCCGGGACCCGGAATTTCTCGTCTGC

GGGGATGATTTAGTCGTGGTGGCTGAGAGTGATGGCATTGACGAGGATAGAGCAGCCCTG

AGAGCCTTCACGGAGGCTATGACCAGGTATTCTGCTCCACCCGGAGATGCTCCGCAGCCC

ACATACGACCTTGAGCTCATTACATCTTGCTCCTCCAACGTCTCCGTGGCACAGGACAAC

AAGGGGAAGAGGTACTATTACCTCACCCGTGACCCCACTACACCCCTAGCCCGTGCGGCT

TGGGAAACAGCTCGTCACACTCCAGTTAACTCCTGGTTAGGCAACATCATCATGTACGCG

CCTACCATCTGGGTGCGCATGGTAATGATGACACATTTTTTCTCCATACTTCAATCCCAG

GAGATACTTGATCGACCCCTTGACTTTGA

>MT502224

ACGGAACGGCTTTACTGCGGGGGCCCTATGTTCAACAGCAAGGGGGCCCAGTGTGGTTAT

CGCCGTTGCCGCGCCAGTGGAGTTCTGCCCACCAGCTTTGGTAACACAATAACTTGTTAC

ATCAAGGCCACAGCGGCTGCGAGAGCTGCGGGCCTTCGAAACCCGGACTTTCTTGTCTGC

GGAGATGATTTGGTCGTGGTGGCAGAAAGTGATGGCGTCAACGAGGATGGAGCAGCCCTG

AGAGCTTTCACGGAGGCTATGACCAGGTATTCTGCTCCGCCCGGAGATGCTCCACAGCCC

ACCTACGACCTCGAGCTCATTACATCTTGCTCCTCCAACGTCTCTGTAGCGCGGGACTAC

AAGGGGAAGAGGTACTATTACCTCACCCGTGATCCCACTACTCCCCTGGCCCGTGCGGCT

TGGGAAACAGCTCGTCACACTCCAGTCAACTCCTGGTTGGGCAACATCATCATGTACGCG

CCTACCATCTGGGTGCGCATGGTAATGATGACACACTTCTTCTCCATACTCCAATCCCAG

GAGATACTTGATCGGTCCCTTGACTTTGA

>MT502225

ACGGAGCGGCTTTACTGCGGGGGCCCCATGTTCAACAGTAAAGGGGCCCAGTGTGGTTAC

CGCCGTTGCCGTGCTAGTGGGGTCCTGCCTACCAGCTTCGGCAACACGATCACTTGTTAC

ATCAAGGCTACAGCGGCTGCAAAGGCCGCAGGCCTCCGGAACCCGGAGTTTCTCGTCTGC

GGAGATGATCTAGTCGTGGTGGCTGAGAGTGATGGCGTCGAGGAGGATAGAGCAGCCCTG

GGAGCCTTCACGGAGGCTATGACCAGGTATTCTGCTCCACCCGGAGATGCTCCACAGCCT

ACCTACGACCTTGAGCTCATTACATCTTGCTCTTCCAACGTCTCCGTGGCGCGGGACGAC

AAGGGGAGGAGGTATTATTACCTCACCCGTGATCCCACTACTCCCCTAGCCCGTGCGGCT

TGGGAAACAGCTCGTCACACTCCAGTTAATTCCTGGTTGGGCAACATCATCATGTACGCG

CCTACCATTTGGGTGCGCATGGTAATGATGACACATTTTTTCTCCATACTCCAATCCCAG

GAAATACTTGATCAACCCCTTGACTTTGAAA

>MT502226

ACGGAGCGGCTTTACTGCGGGGGTCCTATGTTCAACAGCAAGGGGGCCCAGTGTGGTTAT

CGCCGCTGCCGTGCCAGTGGAGTTCTGCCTACCAGCTTTGGCAACACAATCACTTGTTAC

ATCAAGGCCACGGCGGCTGCGAAGGCCGCAGGCCTCCGGGACCCGGACTTTCTTGTCTGC

GGAGATGATCTGGTCGTGGTGGCCGAGAGTGATGGCGTCGAAGAGGATAGAGCAGCCCTG

AGAGCCTTCACGGAGGCTATGACCAGGTATTCTGCTCCACCCGGAGATGCCCCACAGCCC

ACCTACGACCTTGAGCTCATTACATCTTGCTCCTCCAACGTCTCTGTGGCACTGGACAAC

AAAGGGAAGAGGTATTATTACCTCACCCGTGATCCCACTACTCCCTTAGCCCGTGCGGCT

TGGGAAACAGCTCGTCACACTCCAGTTAACTCCTGGCTAGGCAACATCATCATGTACGCG

CCTACCATCTGGGTGCGCATGGTAATGATGACGCACTTTTTCTCCATACTCCAATCCCAG

GAGATACTTGACCGCCCCCTTGACTTCGA

>MT502227

ACGGAACGGCTTTACTGCGGTGGCCCTATGTTCAACAGCAAGGGGGCCCAGTGTGGTTAT

CGCCGTTGCCGTGCCAGTGGAGTTTTGCCTACCAGCTTCGGCAACACAATCACTTGTTAC

ATCAAGGCCACAGCGGCTGCAAAGGCTGCAGGCCTCCGGAACCCGGACTTTCTTGTCTGC

GGAGATGATTTAGTCGTAGTGGCTGAGAGCGACGGCGTCGAGGAGGATAGGGCAGCCCTG

AGAGCCTTCACGGAGGCTATGACCAGGTATTCTGCTCCACCCGGAGACGCTCCACAGCCC

ACCTACGACCTTGAGCTCATCACATCTTGCTCTTCTAACGTCTCCGTGGCACTGGACAAC

AAGGGGCGAAGGTACTATTACCTCACCCGTGATGCCACTACTCCCCTAGCCCGTGCGGCT

TGGGAAACAGCTCGTCACACTCCAGTTAACTCCTGGTTAGGCAACATCATCATGTACGCG

CCTACCATCTGGGTGCGCATGGTAATGATGACACATTTTTTCTCCATACTCCAATCCCAG

GAGATACTTGACCGACCCCTTGACTTCGAAA

>MT502228

ACGGAGCGGCTTTACTGCGGGGGCCCTATGTTTAACAGCAAAGGGGCCCAGTGTGGTTAT

CGCCGTTGCCGTGCCAGTGGAGTTTTGCCCACCAGCTTCGGCAATACTATCACTTGTTAC

ATCAAGGCCACAGCGGCTGCGAGGGCCGCAGGCCTCCGGAACCCGGACTTTCTTGTCTGC

GGAGATGATCTAGTTGTGGTGGCTGAAAGTGATGGCGTCGACGAGGACAGAGCAGCCCTG

AGAGCCTTCACGGAGGCTATGACCAGGTATTCTGCTCCACCCGGAGATGCTCCACAGCCC

ACCTATGACCTTGAGCTCATCACATCTTGCTCCTCTAACGTCTCCGTAGCACGGGACAAC

AAGGGGAAGAGGTACTATTACCTCACCCGTGATGCTACTACTCCCCTGGCCCGCGCGGCT

TGGGAAACAGCTCGTCACACTCCAGTTAACTCCTGGTTGGGTAACATCATCATGTACGCG

CCTACTATATGGGTGCGCATGGTAATGATGACACATTTTTTCTCCATACTCCAATCCCAG

GAGATACTTGATCGCCCCCTTGACTTTGA

>MT502229

ACGGAACGGCTTTACTGCGGGGGCCCTATGTTCAACAGCAAAGGGGCCCAGTGTGGTTAT

CGCCGTTGCCGTGCTAGTGGAGTTCTGCCTACCAGCTTTGGCAACACAATCACGTGTTAC

ATCAAGGCTACAGCGGCTGCTAGGGCCGCAGGCCTCCGGAACCCGGACTTTCTTGTCTGC

GGGGATGATCTAGTCGTGGTGGCTGAGAGTGATGGCGTCGACGAGGATAGAGCAGTCCTG

AGAGCCTTCACGGAGGCTATGACCAGGTATTCTGCTCCACCCGGAGATGCTCCACAGCCT

ACCTACGACCTTGAGCTCATTACATCTTGCTCCTCTAACGTCTCCGTGGCGCTGGACGAC

AAGGGGAAGAGGTACTATTACCTCACCCGTGATGCCACTACTCCCCTGGCCCGTGCGGCT

TGGGAAACAGCTCGCCACACTCCAGTTAACTCCTGGTTGGGCAACATTATCATGTACGCG

CCTACCATCTGGGTGCGCATGGTAATGATGACACACTTTTTCTCCATACTCCAGTCCCAG

GAGGTACTTGATCGCCCCCTTGACTTTG

>MT502230

ACGGAGCGGCTTTACTGCGGGGGCCCTATGTTCAACAGCAAGGGGACCCAGTGTGGTTAT

CGCCGTTGCCGCGCCAGCGGAGTTCTGCCTACCAGTTTCGGCAACACAATCACTTGTTAC

ATCAAGGCCACAGCGGCTGCGAGGGCCGCGGGCCTCCGGAACCCGGACTTTCTTGTCTGC

GGGGATGATCTGGTCGTGGTGGCTGAGAGTGATGGCGTCAACGAGGATGGAGCAGCCCTG

AGAGCCTTCACGGAGGCTATGACCAGGTATTCTGCTCCGCCCGGAGATGCTCCACAGCCC

ACCTACGACCTTGAGCTCATCACATCTTGCTCCTCCAACGTCTCCGTAGCGCGGGACAAC

AAGGGGAAGAGGTATTACTACCTCACCCGTGATGCCACTACTCCCCTGGCCCGTGCGGCT

TGGGAAACAGCTCGTCACACTCCAGTTAACTCCTGGTTGGGCAACATCATCATGTACGCG

CCTACCATCTGGGTACGCATGGTAATGATGACACACTTTTTCTCCATACTCCAATCCCAG

GAGATACTTGATCGACCCCTTGACTTTGA

>MT502231

ACGGAGCGGCTTTACTGCGGAGGCCCTATGTTCAACAGCAAAGGGGCCCAGTGTGGTTAT

CGCCGCTGCCGTGCTAGTGGAGTTCTGCCTACCAGCTTCGGCAACACAATCACTTGTTAC

ATCAAGGCCACGGCGGCCGTAAGGGCCGCAGGCCTCCGGAACCCGGACTTTCTTGTCTGC

GGAGATGATCTAGTCGTGGTGGCCGAGAGTGACGGCGTCGACGAGGATAGAGTAGCCCTG

AGAGCCTTCACGGAGGCTATGACCAGGTATTCTGCTCCACCCGGAGATGCTCCACAGCCT

ATCTACGACCTTGAGCTCATTACATCTTGCTCCTCTAACGTCTCCGTAGCACGGGACAAC

AAGGGGAAGAGGTATTATTACCTCACCCGTGATGCCACCACTCCCCTCGCCCGAGCGGCT

TGGGAAACAGCTCGTCACACTCCAGTTAACTCCTGGTTGGGCAACATCATCATGTACGCG

CCTACCATCTGGGTGCGCATGGTAATGATGACCCACTTCTTCTCTATACTCCAATCCCAG

GAGATACTTGACCGCCCCCTTGACTTTGA

>MT502232

ACGGAACGGCTTTACTGTGGGGGCCCCATGTTCAACAGCAAAGGGGCTCAGTGTGGTTAT

CGCCGTTGCCGCGCCAGTGGAGTCTTGCCTACCAGCTTCGGCAACACGATCACGTGTTAC

ATCAAGGCTACGGCGGCTGCTAGGGCCGCAGGTCTCCGGAACCCGGACTTTCTTGTATGC

GGGGATGATTTAGTCGTGGTGGCTGAGAGCGATGGCGTCGACGAGGATAGAGCAGCCCTG

AGAGCTTTCACGGAGGCTATGACCAGGTATTCTGCTCCACCCGGAGATGCTCCACAGCCT

ACTTATGACCTTGAGCTCATTACATCTTGCTCCTCCAACGTCTCCGTAGCACGGGACGAC

AAGGGGAAGAGGTACTATTACCTCACCCGTGATGCCACTACTCCCCTGGCCCGTGCGGCT

TGGGAAACAGCACGTCACACTCCAGTTAACTCCTGGTTGGGCAACATCATCATGTACGCG

CCTACCATCTGGGTGCGCATGGTAATGATGACACATTTTTTCTCCGTACTCCAGTCCCAG

GAGGTACTTGATCGCCCCCTAGATTTTGA

>MT502233

ACGGAGCGGCTTTACTGCGGGGGCCCTATGTTCAACAGCAAGGGGGCCCAGTGTGGTTAT

CGCCGCTGTCGTGCTAGTGGAGTTCTACCTACCAGCTTCGGCAACACAATAACTTGTTAC

ATCAAAGCCACAGCAGCCGCGAGGGCTGCAGGCCTCCGGAACCCGGACTTCCTCGTCTGC

GGAGATGATTTGGTCGTGGTAGCTGAGAGCGATGGCGTCGAGGAGGATAGAGCAGCCCTG

AGAGCCTTCACGGAGGCTATGACCAGGTACTCTGCTCCACCTGGAGATGCCCCACAGCCC

ACCTACGACCTTGAGCTCATTACATCTTGCTCTTCCAACGTCTCCGTGGCATTGGACGGC

AAGGGGAGGAGGTACTATTACCTCACTCGTGATGCCACTACTCCCCTGGCCCGCGCTGCT

TGGGAAACAGCTCGTCACACTCCAGTTAACTCCTGGTTAGGCAACATCATCATGTACGCG

CCTACCATCTGGGTGCGTATGGTGATGATGACACACTTTTTCTCCATACTCCAAGCCCAG

GAGATACTTGACCGACCCCTTGACTTTGAAA

>MT502234

ACGGAGCGGCTTTACTGCGGGGGCCCTATGTTTAACAACAAGGGGGCCCAGTGTGGTTAT

CGCCGTTGTCGTGCCAGTGGAGTTCTGCCTACCAGCTTTGGCAACACTATCACTTGTTAC

ATCAAGGCCACAGCGGCTGCGAGGGTCGCAGGCCTCCGGAACCCGGACTTTCTTGTCTGC

GGAGATGATTTAGTCGTGGTGGCTGAGAGTGATGGCGTCGACGAGGATAAGGCAGCCCTG

AGAGCTTTCACGGAAGCTATGACCAGGTATTCTGCTCCACCCGGAGATGCTCCACAGCCC

ACCTACGATCTTGAGCTCATCACATCTTGCTCCTCCAACGTCTCCGTAGCACGCGACAAC

AAGGGGAAGAGGTATTATTACCTCACCCGTGATGCCACTACTCCCCTGGCCCGCGCGGCT

TGGGAAACAGCTCGTCACACTCCAGTTAACTCCTGGTTGGGCAACATCATCATGTACGCG

CCTACCATTTGGGTGCGCATGGTAATGATGACACACTTTTTCTCCATACTCCAATCCCAG

GAGATACTTGATCGCCCCCTTGACTTTGA

>MT502235

ACGGAACGGCTTTACTGCGGGGGCCCTATGTTTAACAGCAAAGGGGCCCAGTGTGGTTAT

CGCCGCTGCCGTGCCAGTGGAGTTTTGCCTACCAGCTTCGGCAACACGATCACGTGTTAC

ATCAAGGCTACGGCGGCTGCTAGGGCCGCAGGCCTCCGGAACCCGGACTTTCTTGTCTGC

GGGGATGATCTAGTCGTGGTGGCTGAGAGTGATGGCGTCGACGAGGATAGAGCAGCCCTG

AGAGCTTTCACGGAGGCTATGACCAGGTATTCTGCTCCACCCGGAGATGCTCCACAGCCC

ACCTACGACCTTGAGCTCATTACATCTTGCTCCTCCAACGTCTCCGTAGCACGGGATGAC

AAGGGGAAGAGGTACTATTACCTCACTCGTGATGCCACTACTCCCTTGGCCCGTGCAGCT

TGGGAAACAGCTCGTCACACTCCAGTCAACTCCTGGTTGGGCAACATTATCATGTACGCG

CCTACCATCTGGGTGCGTATGGTAATGATGACGCACTTTTTCTCCATACTCCAGTCCCAG

GAGGTACTTGATCGCCCCCTTGACTTTGA

>MT502236

ACGGAGCGGCTTTACTGCGGGGGCCCTATGTTCAACAGCAAGGGGGCCCAGTGCGGTTAT

CGCCGTTGCCGCGCTAGTGGAGTTCTGCCTACCAGCTTCGGTAACACAATCACTTGTTAC

ATCAAGGCCACAGCAGCTGCAAAGGCCGCAGGCCTCCGAAACCCGGACTTTCTTGTCTGC

GGAGATGATCTGGTCGTCGTGGCTGAGAGTGACGGCGTCGATGAGGATAGAGCAGCCCTG

AGAGCCTTCACGGAGGCTATGACCAGGTATTCTGCTCCACCCGGAGATGCCCCACAGCCC

ACTTACGACCTTGAGCTTATTACATCTTGCTCCTCCAACGTCTCCGTGGCACGAGACGAC

AAGGGAAGGAGGTACTACTACCTCACCCGTGATGCCACTACTCCCCTGGCCCGTGCGGCT

TGGGAAACAGCTCGTCACACTCCAGTTAACTCCTGGTTGGGCAACATCATCATGTACGCG

CCTACCATCTGGGTGCGCATGGTAATGATGACGCATTTTTTCTCCATACTCCAATCCCAG

GAGATGCTTGAACGGTCCCTTGATTTTGAAA

>MT502237

ACGGAACGGCTTTACTGCGGGGGCCCTATGTTCAACAGTAAGGGGGCCCAGTGTGGTTAT

CGCCGTTGCCGTGCCAGTGGAGTTCTGCCCACCAGCTTTGGCAACACACTCACTTGTTAC

ATCAAGGCCACGGCGGCCGCGAGAGCCGCCGGCCTCCGGAACCCGGACTTTCTTGTCTGC

GGAGATGATTTAGTCGTGGTGGCTGAAAGCGATGGCGTTGACGAGGATAGAGCAGCCCTG

GGAGCCTTCACGGAGGCTATGACCAGATATTCTGCTCCACCCGGAGATGCTCCACAGCCT

ACTTACGACCTTGAGCTTATTACATCCTGCTCCTCTAACGTCTCCGTGGCACTGGACAAC

AAGGGGAAGAGGTACTATTACCTCACCCGTGATGCCACCACTCCCCTAGCCCGTGCGGCT

TGGGAAACAGCTCGTCACACTCCAGTCAACTCCTGGTTAGGCAACATCATCATGTACGCG

CCTACTATCTGGGTGCGCATGGTAATGATGACACATTTTTTCTCCATACTGCAATCCCAG

GAGATACTTGATCGGTCCCTTGACTTTGA

>MT502238

ACGGAGCGGCTTTACTGCGGGGGCCCCATGTTCAACAGTAAAGGGGCCCAGTGTGGTTAC

CGCCGCTGCCGTGCCAGTGGAGTTCTGCCTACCAGTTTCGGCAACACACTCACTTGTTAC

ATCAAAGCCACAGCGGCTGCAAGGGCCGCAGGCCTCCGGAACCCGGAGTTCCTTGTTTGC

GGAGATGATCTGGTCGTGGTGGCTGAGAGTGATGGCGTCGACGAGGATAGAGCAGCCCTG

AGAGCCTTCACGGAGGCTATGACCAGGTATTCTGCTCCACCCGGAGATGCTCCACAGCCT

ACCTACGACCTTGAGCTCATTACATCTTGCTCTTCCAACGTCTCCGTGGCACGGGACGAC

AAGGGAAAGAGGTACTATTACCTCACCCGTGATCCCACCACTCCCCTAAGCCGTGCGGCT

TGGGAAACAGCTCGTCACACTCCAGTTAACTCCTGGTTGGGCAACATCATCATGTACGCG

CCTACCATTTGGGTGCGCATGGTAATGATGACCCACTTTTTCTCCATACTCCAATCCCAG

GAGATACTTGATCGGTCCCTTGACTTTGAAA

>MT502239

ACGGAACGGCTTTACTGCGGGGGCCCTATGTTTAACAGCAAAGGGGCCCAGTGTGGTTAT

CGCCGTTGCCGTGCTAGTGGAGTTCTGCCTACCAGCTTTGGCAACACAATCACTTGCTAC

ATCAAGGCTACGGCGGCTGCTAGAGCCGCTGGCCTCCGGAACCCGGACTTTCTTGTCTGC

GGGGACGATCTAGTCGTGGTGGCCGAGAGTGATGGCGTCGACAAGGATAAAGCAGCCCTG

AGAGCCTTCACGGAGGCTATGACCAGGTATTCTGCTCCACCCGGAGATGCTCCACAGCCT

ACCTACGACCTTGAGCTCATTACATCTTGCTCCTCTAACGTCTCCGTAGCACGGGACGAC

AAGGGGAAGAGGTACTACTACCTCACCCGTGATCCCACAACTCCCCTGGCCCGTGCGGCT

TGGGAAACAGCCCGTCACACTCCAGTTAACTCCTGGTTGGGGAACATCATCATGTACGCG

CCTACCATCTGGGTACGCATGGTACTGATGACACACTTTTTCTCCATACTCCAGTCCCAG

GAGATACTTGATCGCCCCCTTGACTTTGA

>MT502240

ACGGAACGGCTTTACTGCGGGGGCCCTATGTTCAACAGCAAGGGGGCCCAGTGTGGATAT

CGTCGTTGCCGTGCCAGTGGAGTCCTGCCTACCAGCTTTGGCAATACAATCACTTGTTAC

ATCAAGGCTACGGCGGCTGCTAGAGCCGCAGGTCTCCGAGACCCGGACTTTCTTGTTTGC

GGGGATGATCTAGTCGTGGTGGCTGAGAGCGATGGCGTCGACGAGGATAGAGCGGCCCTG

AGAGCCTTCACGGAGGCTATGACCAGGTATTCCGCTCCACCCGGAGATGCTCCACAGCCT

ACCTACGACCTTGAGCTCATTACATCTTGCTCCTCCAACGTCTCCGTAGCACGAGACGAC

AAGGGGAAGAGGTACTATTACCTCACCCGTGATGCCACTACCCCCCTTGTCCGTGCGGCC

TGGGAAACAGCTCGTCACACTCCAGTTAACTCCTGGTTGGGCAACATTATCATGTACGCG

CCTACCATCTGGGTGCGCATGGTAATGATGACTCACTTTTTCTCCATACTCCAGTCCCAG

GAGATACTTGATCGCCCCCTTGACTTTGA

>MT502241

ACGGAACGGCTTTACTGCGGGGGCCCCATGTTCAACAGCAAAGGGGCCCAGTGTGGTTAT

CGCCGTTGCCGTGCCAGTGGAGTCCTGCCTACCAGCTTCGGCAACACCATCACTTGTTAC

ATCAAGGCCACAGCGGCTGCGAAGGCCGCAGGCCTCCGGAACCCGGACTTTCTTGTCTGC

GGAGATGATTTGGTTGTGGTGGCTGAAAGTGATGGCGTCGATGAGGACAGAACAGCCCTG

AGAGCCTTCACGGAGGCTATGACCAGGTATTCTGCTCCACCCGGAGATGCTCCACAGCCC

ACCTACGACCTTGAGCTCATTACATCTTGCTCTTCCAACGTCTCCGTAGCACGGGACAAC

AAGGGGAAAAGGTACTATTACCTCACCCGTGATGCCACTACTCCCCTGGCCCGTGCGGCT

TGGGAAACAGCTCGTCACACTCCAGTTAACTCCTGGTTAGGCAACATCATCATGTACGCG

CCTACCATCTGGGTGCGCATGGTAATGATGACACACTTTTTCTCCATACTCCAATCCCAG

GAGATACTTGATCGCCCCCTTGATTTTGA

>MT502242

ACGGAACGGCTTTACTGCGGGGGCCCCATGTTCAACAGCAAAGGGGCTCAGTGTGGTTAC

CGCCGTTGTCGTGCTAGTGGAGTTCTGCCTACCAGCTTCGGCAACACGATCACTTGTTAC

ATCAAGGCCACAGCGGCCGCAAGGGCCGCAGGCCTTCGCAACCCGGAGTTTCTCGTCTGC

GGAGATGATTTGGTCGTGGTGGCTGAAAGTGATGGCGTCGATGAGGATAGAGCGACCCTG

AGAGCCTTCACGGAGGCTATGACCAGGTATTCTGCTCCACCCGGAGATGCTCCACAGCCC

ACCTATGACCTTGAGCTCATTACATCTTGCTCTTCCAACGTCTCTGTGGCACGGGACGAC

AAGGGAAGGAGGTACTACTACCTCACCCGTGATGCTACTACCCCCCTGGCCCGTGCGGCT

TGGGAAACAGCTCGTCACACTCCAGTTAACTCCTGGTTGGGTAACATCATCATGTACGCG

CCTACTATCTGGGTGCGCATGGTAATGATGACTCACTTTTTCTCCATACTTCAATCCCAG

GAGATACTTGATCGACCCCTCGACTTTGAAA

>MT502243

ACGGAGCGGCTTTACTGCGGGGGCCCTATGTTCAATAGCAAGGGGGCCCAGTGCGGTTAT

CGCCGTTGCCGTGCCAGTGGAGTCCTGCCTACTAGCTTTGGCAACACAATCACTTGTTAC

ATCAAGGCCACAGCGGCTGCAAAGGCCGCAGGCCTCCGGAACCCGGATTTTCTTGTCTGC

GGAGATGATCTGGTCGTGGTAGCTGAGAGTGATGGTGTCGACGAGGATAGAGCAGCCCTG

AGAGCCTTCACGGAGGCTATGACCAGGTATTCTGCTCCACCCGGAGATGCTCCACAGCCC

ACCTACGATCTTGAGCTCATTACATCTTGCTCTTCTAACGTCTCTGTGGCACTGGACAAC

AAGGGGAAGAGGTATTATTACCTCACCCGTGATGCCACTACTCCCCTGACCCGTGCGGCT

TGGGAAACAGCTCGTCACACTCCAGTTAACTCCTGGTTAGGCAACATCATCATGTACGCG

CCTACCATCTGGGTGCGCATGGTAATGATGACACACTTCTTCTCCATACTCCAATCCCAG

GAGATACTTGACCGCCCCCTTGACTTTGAAA

>MT502244

GGCTCTATGTTCAACAGCAAGGTGGCCCAGTGCGGTTAT

CGCCGCTGCGGTCCCAGTGGAGTTCTTACCACCAGCTTTGGCAATTCAATCACTTGTTAC

ATCAAGTCCACAGCGGCTGCGAAGGTCGCAGGCCTCCGGGACCCGGACTTTGTTGTCTGC

GGAGACGATCTGGTCGTGGTGGGTGAGAGTGACGGCGTCGATGAGGATAGAGCAGCCCTG

AGAGCCTTCACGGAGGCTATGACCAGGTATTCTGCTCCACCCGGAGATGCTCCACAGCCC

ACCTACGACCTCGAGCTCATTACATCTTGCTCCTCCAACGTCTCTGTGGCATTGGACAAC

AAGGGGAAGAGGTATTATTACCTCACCCGCGATGCTACCACTCCCCTGGCCCGTGCGGCT

TGGGAAACAGCTCGTCACACTCCAGTTAACTCCTGGTTAGGCAATATCATCATGTACGCG

CCTACCATTTGGGTGCGCATGGTGATGATGACACATTTTTTCTCCATACTCCAATCCCAG

GAGATACTTGACCGACCCCTTGACTTTGA
