# Supplementary material for: Prevalence of Naturally-Occurring NS5A and NS5B Resistance-Associated Substitutions in Iranian Patients With Chronic Hepatitis C Infection
Source: Front Microbiol. 2021 Jan 28;11:617375. doi: 10.3389/fmicb.2020.617375 (PMC7876467; doi:10.3389/fmicb.2020.617375)
Supplement: Supplementary file 2 [file Table_1.doc]

| **Primer name** | **HCV genotype** | **HCV gene** | **PCR round** | **Primer Strand** | **Primer sequence (3’-5’)** |
| --- | --- | --- | --- | --- | --- |
| FO1ans5a | 1a | NS5A | First | Forward | GCAGTGCAGTGGATGAACCGG |
| RO1ans5a | 1a | NS5A | First | Reverse | CATGCCCGTTACGTAGTGGAAG |
| FI1ans5a | 1a | NS5A | Nested | Forward | GGTTGATAGCCTTCGCCTCTCG |
| RI1ans5a | 1a | NS5A | Nested | Reverse | CTGCAGACACCCTCCACAACG |
| FO3ans5a | 3a | NS5A | First | Forward | CAGCCCCCTGACAACCAACCAAAC |
| RO3ans5a | 3a | NS5A | First | Reverse | CTCCCCGCCACACACCCTTATATCC |
| FI3ans5a | 3a | NS5A | Nested | Forward | CRAGGGTCACKGCRCTGCTG |
| RI3ans5a | 3a | NS5A | Nested | Reverse | TGCYGGGTCCGGTGGTGTAC |
| FO1ans5b1 | 1a | NS5B-1 | First | Forward | GCCTATCAACGCACTGAGCAACTC |
| RO1ans5b1 | 1a | NS5B-1 | First | Reverse | CACAGACAAGCATGGTGCAGTCC |
| FI1ans5b1 | 1a | NS5B-1 | Nested | Forward | GTGTATTCTACCACCTCACGCAG |
| RI1ans5b1 | 1a | NS5B-1 | Nested | Reverse | GCTGCTTGGGCCTTGATGTAG |
| FO1ans5b2 | 1a | NS5B-2 | First | Forward | TGGCCATTAAGTCCCTCACCG |
| RO1ans5b2 | 1a | NS5B-2 | First | Reverse | TCGGTTGGGAAGGAGGTAGATG |
| FI1ans5b2 | 1a | NS5B-2 | Nested | Forward | GGGGAAAACTGCGGCTATCG |
| RI1ans5b2 | 1a | NS5B-2 | Nested | Reverse | CAAGCAGGAGTAGGCAAAACCAG |
| FO3ans5b1 | 3a | NS5B-1 | First | Forward | ACCGGCGCCTTGATAACACCATG |
| RO3ans5b1 | 3a | NS5B-1 | First | Reverse | CGCAGTAAAGCCGTTCCGTGAGG |
| FI3ans5b1 | 3a | NS5B-1 | Nested | Forward | CATCAGCCCACTCAGCAATTCC |
| RI3ans5b1 | 3a | NS5B-1 | Nested | Reverse | CACTTTCTTGGCCTCCGGTTC |
| FO3ans5b2 | 3a | NS5B-2 | First | Forward | GTTCTCGTATGACACCCGCTGCTTTG |
| RO3ans5b2 | 3a | NS5B-2 | First | Reverse | GATGTCTCCAAGCTCGTAGGGGGG |
| FI3ans5b2 | 3a | NS5B-2 | Nested | Forward | CAGGACATCAGGGTGGAAGAGGAG |
| RI3ans5b2 | 3a | NS5B-2 | Nested | Reverse | CGCGACCCTATTGAGCTCTACCG |

**Table S1.** Sequences of primers used for amplification of HCV-1a and -3a NS5A and NS5B regions.

Nested PCR was used for amplification of each region. NS5B region was divided in two overlapping sequences named as ns5b1 and ns5b2 and amplified using two specific different primer pairs. The size of final PCR products obtained from specific inner primer pairs for NS5A,NS5B (ns5b1,ns5rb2)of each HCV-1a and 3a : HCV-1a: NS5A 510 bp, NS5B1 797,NS5b2 928bp. HCV-3a: NS5A 348bp, NS5B1:691bp,NS5B2: 768bp.
